# Efficacy and safety of pegzilarginase in arginase 1 deficiency (PEACE): a phase 3, randomized, double-blind, placebo-controlled, multi-centre trial

**DOI:** 10.1016/j.eclinm.2023.102405

**Published:** 2024-01-12

**Authors:** Rossana Sanchez Russo, Serena Gasperini, Gillian Bubb, Linda Neuman, Leslie S. Sloan, George A. Diaz, Gregory M. Enns, PEACE Investigators

**Affiliations:** a Department of Human Genetics, Emory University School of Medicine, Atlanta, GA, United States; b Paediatric Department, Fondazione IRCSS San Gerardo dei Tintori, Monza, Italy; c Aeglea BioTherapeutics, Inc., Austin, TX, United States; d Department of Genetics and Genomic Sciences, Icahn School of Medicine at Mount Sinai, New York City, NY, United States; e Division of Medical Genetics, Department of Pediatrics, Stanford University School of Medicine and Lucille Packard Children's Hospital, Stanford, CA, United States

**Keywords:** Arginine, Arginase 1, ARG1-D, Enzyme therapy, Gross motor function measure, Guanidino compounds, Hyperargininaemia, Inherited metabolic disorder, Inborn error of metabolism, Mobility, Novel therapeutics, Rare disease, Spasticity, Timed walk test, Urea cycle disorder

## Abstract

**Background:**

Arginase 1 Deficiency (ARG1-D) is a rare debilitating, progressive, inherited, metabolic disease characterized by marked increases in plasma arginine (pArg) and its metabolites, with increased morbidity, substantial reductions in quality of life, and premature mortality. Effective treatments that can lower arginine and improve clinical outcomes is currently lacking. Pegzilarginase is a novel human arginase 1 enzyme therapy. The present trial aimed to demonstrate efficacy of pegzilarginase on pArg and key mobility outcomes.

**Methods:**

This Phase 3 randomized, double-blind, placebo-controlled, parallel-group clinical trial (clinicaltrials.govNCT03921541, EudraCT 2018-004837-34), randomized patients with ARG1-D 2:1 to intravenously/subcutaneously once-weekly pegzilarginase or placebo in conjunction with their individualized disease management. It was conducted in 7 countries; United States, United Kingdom, Canada, Austria, France, Germany, Italy. Primary endpoint was change from baseline in pArg after 24 weeks; key secondary endpoints were change from baseline at Week 24 in Gross Motor Function Measure part E (GMFM-E) and 2-min walk test (2MWT). Full Analysis Set was used for the analyses.

**Findings:**

From 01 May 2019 to 29 March 2021, 32 patients were enrolled and randomized (pegzilarginase, n = 21; placebo, n = 11). Pegzilarginase lowered geometric mean pArg from 354.0 μmol/L to 86.4 μmol/L at Week 24 vs 464.7 to 426.6 μmol/L for placebo (95% CI: −67.1%, −83.5%; *p* < 0.0001) and normalized levels in 90.5% of patients (vs 0% with placebo). In addition, clinically relevant functional mobility improvements were demonstrated with pegzilarginase treatment. These effects were sustained long-term through additional 24 weeks of subsequent exposure. Pegzilarginase was well-tolerated, with adverse events being mostly transient and mild/moderate in severity.

**Interpretation:**

These results support pegzilarginase as the first potential treatment to normalize pArg in ARG1-D and achieve clinically meaningful improvements in functional mobility.

**Funding:**

Aeglea BioTherapeutics.

**Research in context**

**Evidence before this study**

Patients with arginase 1 deficiency (ARG1-D) show persistent hyperargininemia and continued disease progression despite current individualized disease management. The failure of current available treatment options is due to both the practical challenges of a protein-restricted diet rigorous enough to lower plasma arginine levels and its failure to address endogenous arginine production. Aggregate nonclinical and clinical evidence demonstrate the role of arginine in disease pathophysiology, and support the concept that lowering plasma arginine levels has the potential to slow disease progression in patients with ARG1-D. Pegzilarginase has been shown to produce marked, rapid, and sustained reductions in plasma arginine levels in patients with ARG1-D, allowing substantially improved arginine control relative to what can be achieved with current treatment options.

**Added value of this study**

This Phase 3 trial represents the largest group of prospectively evaluated patients and is the first randomized, blinded, placebo-controlled clinical trial in ARG1-D to our knowledge. Pegzilarginase significantly reduced plasma arginine levels, exceeding guideline-recommended levels, and achieved and maintained normal levels. Further, patients treated with pegzilarginase met or exceeded pre-defined and literature-based thresholds for minimum clinically important differences suggesting clinically meaningful improvements in functional mobility. Importantly, these improvements continued to increase in magnitude with long-term treatment in this progressive disorder. Pegzilarginase was well-tolerated, with a safety profile consistent with previous studies, and no new safety signals identified.

**Implications of all the available evidence**

Pegzilarginase is the first disease modifying treatment to normalize plasma arginine levels in the management of ARG1-D. Our results support pegzilarginase as a potentially transformative therapy to normalize arginine, the pathophysiological driver of ARG1-D, and to improve functional mobility outcomes compared with existing management approaches alone.

## Introduction

Arginase 1 Deficiency (ARG1-D; OMIM 207800) is a rare inherited metabolic disorder caused by biallelic pathogenic variants in the *ARG1* gene (HGNC 663) impairing enzymatic activity of arginase 1 (ARG1, EC 3.5.3.1), the final step in the urea cycle.^1^^,^^2^ Plasma arginine (pArg) levels are persistently elevated,^1^^,^^3^^,^^4^ leading to progressive and debilitating neurologic manifestations.^1^^,^^5^, ^6^, ^7^, ^8^, ^9^, ^10^ ARG1-D presents with a distinct neurologic phenotype compared with other urea cycle disorders.^1^^,^^11^^,^^12^ After uneventful neonatal period, clinical symptoms typically become apparent during first years of life, and worsen with cumulative exposure to high pArg and its metabolites, guanidino compounds (GCs).^1^^,^^9^ The hallmark feature of ARG1-D is prominent and progressive lower-limb spasticity leading to gait abnormalities, difficulty walking and climbing stairs, and need for assistive devices. Most patients ultimately develop impairment of gross motor function and mobility, potentially becoming non-ambulatory or reliant on a wheelchair.^5^^,^^6^^,^^8^^,^^12^^,^^13^ Developmental delay and/or developmental regression, cognitive impairment, and seizures may also occur.

The prevalence is estimated to 1:726,000.^14^ The burden of illness is substantial and increases over time as neurologic manifestations progress. Patients require lifelong treatment and monitoring, with strict multimodal management regimens. Continued decline into adulthood is associated with significant morbidity and potential early mortality. The age at death for most published cases is < 50 years.^7^^,^^13^^,^^15^ A systematic literature review found median age of death, for deceased cases, to be 5.7 years.^16^

There are no approved therapies that address the pathophysiologic mechanisms driving clinical onset and progression, and long-term outcomes are generally poor. Standard-of-care consists of dietary protein restriction, essential amino acid supplementation, and symptomatic treatments.^12^ Current guidelines recommend lowering pArg ≤200 μmol/L^12^ but is rarely achievable with dietary restriction,^3^ in part because a substantial proportion of arginine comes from endogenous sources.^17^^,^^18^ Current management is therefore inadequate to alter the disease course. Thereby there is an urgent need for effective treatment that lowers pArg levels, halt disease progression and improves clinical outcomes.

Pegzilarginase is a recombinant, cobalt-substituted and pegylated human ARG1 enzyme therapy with increased catalytic activity due to the cobalt-substitution and prolonged half-life.^19^^,^^20^ In an open-label, Phase 1/2 study in 16 patients managed with standard-of-care (all on protein-restricted diet), treatment with weekly pegzilarginase resulted in pronounced pArg reduction and clinically meaningful improvements in functional mobility.^21^ After 2 years of treatment in the long-term extension (LTE), 100% of patients had pArg <200 μmol/L and 77% normalized pArg levels.^22^ This was the first study to perform functional mobility assessments in ARG1-D. There are no validated or standardized functional assessments available; therefore, clinically important response thresholds established for cerebral palsy,^23^^,^^24^ a population presenting with neurologic features characteristic of ARG1-D such as spasticity and functional mobility deficits, were applied. Clinically important response criteria were met or exceeded on assessments of functional mobility tasks involving aided and unaided walking, running, jumping, sitting, and standing. The magnitude of improvement increased with longer duration of treatment. Importantly, at individual patient level, 10/12 evaluable patients met or exceeded the threshold of clinically important response on ≥1 mobility assessments after 2 years. The remaining were clinically stable and did not decline in functional mobility. These results demonstrated sustained efficacy of pegzilarginase on both biochemical and clinical outcomes, particularly striking given the progressive deterioration in ARG1-D.

The PEACE (Pegzilarginase Effect on Arginase 1 Clinical Endpoints) trial was designed to investigate efficacy and safety of pegzilarginase in lowering arginine levels and improving clinical outcomes, as compared with placebo, when added to standard-of-care in children and adults with ARG1-D. Here, we report the primary results of the double-blind (DB) treatment period and the first 6 months of the subsequent open-label LTE.

## Methods

The trial was conducted in accordance with International Council for Harmonisation (ICH) Good Clinical Practice Guidelines and applicable laws and regulations. The research protocol was approved by all relevant institutional review boards or ethics committees for all study sites. All procedures were in accordance with ethical standards of the responsible committee on human experimentation (institutional and national).

### Trial design and treatment

PEACE was a Phase 3 randomized, double-blind, placebo-controlled trial conducted at 19 sites in 7 countries (United States, United Kingdom, Canada, Austria, France, Germany, and Italy; clinicaltrials.gov NCT03921541, EudraCT 2018-004837-34). It consisted of a screening period, a 24-week randomized DB period, and a subsequent open-label LTE (150 weeks planned duration) in which all patients received pegzilarginase. Patients continued their current individualized disease management regimen for the duration of the trial. Changes to dietary protein intake were minimized during the blinded portions of the study. Patients were randomized in a 2:1 ratio to pegzilarginase or volume-matched placebo administered via 30-min intravenous (IV) infusion in the DB period. Randomization was stratified by severity of prior history of hyperammonaemia to minimize potential bias from possible treatment group imbalance. Based on findings from previous dose escalation study (median final dose: 0.09 mg/kg), the pegzilarginase starting dose was 0.1 mg/kg/week with dose modifications as necessary (possible dosing range: 0.05–0.2 mg/kg/week) to achieve pArg within 50–150 μmol/L at the end of the dosing interval (168-h post-dose). In the LTE, patients initially randomized to pegzilarginase continued their optimized dose from the DB period (pegzilarginase/pegzilarginase) and those randomized to placebo transitioned to 0.1 mg/kg pegzilarginase with dose modifications permitted as appropriate (placebo/pegzilarginase). Patients could be switched to subcutaneous (SC) injection at any point after the first 8 weeks of the LTE, using the same pegzilarginase formulation and dose level as their last IV dose. After the 4th SC dose, injections could be administered at the study site or at home by a qualified health care professional.

### Patients

Key inclusion criteria were: age ≥2 years; documented ARG1-D diagnosis (through elevated pArg, pathogenic variants in *ARG1*, and/or diminished erythrocyte ARG1 activity); pArg ≥250 μmol/L (mean of all screening values); and impairment on any secondary functional mobility assessment. Key exclusion criteria were: symptomatic hyperammonaemia (ammonia ≥100 μmol/L requiring acute care or hospitalization) within 6 weeks before first pegzilarginase dose; extreme mobility impairment (i.e., unable to complete assessments); medical conditions or comorbidities that would preclude study compliance or data interpretation (e.g., severe intellectual disability); prior liver or hematopoietic transplant; or participation in prior pegzilarginase study. Individuals with ongoing or planned initiation of treatment with botulinum toxin–containing regimens during the blinded portion of the study, or surgical or botulinum toxin treatment for spasticity-related complications within 16 weeks before first pegzilarginase dose were also excluded to avoid confounding effects on mobility assessments. Patients receiving ammonia scavengers, anti-epileptic drugs, and/or medications for spasticity were required to be on stable dose for ≥4 weeks before randomization and remain on a stable dose during the blinded period.

All patients and/or their parent/guardian provided written informed consent before initiation of any screening procedures.

### Assessments and outcomes

Although standardized or routinely implemented functional assessments for ARG1-D were lacking, the Phase 1/2 study demonstrated the utility of several measures of functional mobility in this population. These instruments were therefore applied. The 6-min walk test was utilized in the Phase 1/2 study, but was changed to the 2-min walk test (2MWT) in this study, as it has been determined to be comparable to the 6MWT^25^ and its shorter duration made it easier for young and/or easily distracted patients to complete. For each patient, baseline mobility impairment was characterized using the Gross Motor Function Classification System (GMFCS), which evaluates self-initiated movement with emphasis on sitting, walking, ascending stairs, and wheeled mobility, and classifies impairment from Level 1 to 5.^26^ Level 1 indicates an ability to walk, climb stairs without use of railing, and perform gross motor skills such as running and jumping with limited speed, balance, and coordination; higher levels indicate progressively greater impairment and increasing reliance on assistive devices.^26^ Other mobility assessments included the Gross Motor Function Measure (GMFM) parts E and D and 2MWT. The GMFM evaluates unaided mobility (performed without bracing or assistive devices), with GMFM-E capturing tasks involving walking, running, and jumping and GMFM-D sitting and standing.^27^ Individual tasks are scored as: 0 = does not initiate, 1 = initiates, 2 = partially completes, 3 = completes, or NT = not tested; the total score reflects the sum of all scored tasks. Possible scores range from 0 to 72 for GMFM-E (24 tasks) and 0–39 for GMFM-D (13 tasks). Lower scores indicate greater functional mobility impairment. The 2MWT evaluates distance travelled on a flat surface in 2 min (with bracing or assistive devices).^25^^,^^28^ Clinically important response thresholds for these assessments were defined using criteria established for cerebral palsy, as described previously.^21^^,^^29^ Briefly, applied thresholds for clinically important response were based on score change from baseline stratified by GMFCS level 1 to 3 for GMFM-E/GMFM-D and a 9% change from baseline in distance travelled for all patients for the 2MWT (full description available in Supplementary Information).^23^^,^^24^

The primary efficacy endpoint was change from baseline in pArg at Week 24 (end of the DB period). The key secondary endpoint was functional mobility as assessed through mean change from baseline in GMFM-E score or 2MWT distance at Week 24. Other secondary efficacy endpoints reported here include change from baseline in GMFM-D score, percentage of patients with pArg <200 μmol/L (guideline level)^12^ and percentage of patients with pArg in the normal range (defined in the study as 40–115 μmol/L)^4^; change from baseline in GCs (argininic acid, guanidinoacetic acid, α-keto-δ-guanidinovaleric acid, and alpha-N-acetylarginine); and change from baseline in ornithine. Sampling for biochemical assessments was conducted at 168-h post-dose (end of dosing interval, prior to next dose), and analyses were performed by ultra-high performance liquid chromatography with tandem mass spectrometry (UHPLC-MS/MS).

Safety and tolerability were evaluated through adverse events (AEs), physical examinations, laboratory testing, and assessment of anti-drug antibodies (ADAs) against pegzilarginase and/or polyethylene glycol (PEG). AEs of special interest (AESIs) included hypersensitivity reactions (HSRs) and injection site reactions (ISRs) (as potential occurrences with a biologic/enzyme therapy) and hyperammonaemic episodes (as known disease occurrences). Hyperammonaemic episodes were defined as ammonia >100 μmol/L with related symptoms requiring hospitalization or emergency room treatment. AEs were coded using Medical Dictionary for Regulatory Activities (MedDRA) version 24.0.

An independent Safety Review Committee (SRC) composed of individuals with pertinent medical expertise served in advisory capacity to the Sponsor to provide an additional level of oversight to minimize the chance that clinical study patients were exposed to unreasonable or unnecessary risks. The members of the independent SRC had access to unblinded individual patient-level safety data as needed. SRC data review meetings were to be scheduled every three months after the first patient was dosed during the remaining portion of the study timeline with additional ad hoc meetings as deemed necessary. Prior to the SRC meetings, the SRC members were provided copies of any study safety suspected unexpected serious adverse reactions (SUSAR), alert letters, cumulative SAE listing from the study safety database. In addition, unblinded cumulative data reports were prepared by an unblinded biostatistician. During closed sessions, restricted to the SRC members and the unblinded biostatistician, the SRC members confidentially reviewed the data, deliberated, and determined the SRC recommendations.

### Sample size

In the Phase 1/2 study, pArg levels at 24 weeks post-baseline were reduced to −2.13 on the log_2_ scale, representing a 77% decrease from baseline, and given that it is unlikely that placebo would have any true treatment effect on pArg levels at 24 weeks, the reduction at 24 weeks for placebo was estimated to be 0. Under these assumptions, a total sample size of 30 patients, with 20 patients assigned to pegzilarginase and 10 patients assigned to placebo, was determined adequate to provide >95% power to detect statistically significant between-group differences in the primary endpoint at 2-sided significance level of α = 0.05.

### Randomisation

Central randomization was used to minimize bias, and executed by a Sponsor designee (SDC, Statistics and Data Corporation [Tempe AZ, USA]). The computer-generated randomization schedule was provided to the Interactive Response Technology vendor (Cenduit [Durham NC, USA]) in which the randomization was executed during the study. Randomization was stratified by severity of prior history of hyperammonaemia to avoid potential imbalance in this factor across randomized treatment groups.

### Blinding

All site personnel involved in the study, including patients, families, caregivers, Investigators, expert assessors of relevant endpoints, and all sponsor and contract personnel were blinded to the patient's randomized treatment assignment to minimize potential biases in assessment of safety and clinical outcomes. Each site had an unblinded pharmacist, and an unblinded physician where required, to manage protocol-defined dose adjustments. Laboratory results for arginine, ornithine, and guanidino compounds had the potential to unblind the Investigator to patients' treatment groups. Therefore, results for these laboratory tests performed during the 24-week DB period were not provided to the Investigator or other blinded individuals until all patients had completed the blinded portion of the study and formal unblinding of the 24-week DB period had occurred.

### Data handling

An electronic data capture system (Medidata Rave®, New York NY, USA) was used for the collection of clinical data at the investigational sites. Laboratory data was held within individual laboratories’ databases, and all data (clinical, laboratory, and interactive response system) was merged into the data analysis database held by eClinical Solutions LLC (Mansfield MA, USA).

### Statistical analysis

Five analysis sets were defined for the study: consented set (all patients who signed an ICF. This population was used to report screening data), randomized set (all patients in the consented set who were randomized to a blinded study treatment), full analysis set (all patients who were randomized and received a least 1 dose of blinded study treatment), per-protocol set (all patients in the full analysis set who had adequate exposure without important protocol deviations that directly impacted efficacy analyses), pharmacokinetic set (all patients who received at least 1 dose of pegzilarginase with sufficient pharmacokinetic data to permit meaningful analysis). For efficacy summary and analysis comparing pegzilarginase versus placebo, patients were categorized according to the treatment to which they were randomized. For safety analyses, patients were categorized according to the treatment that they actually received. Safety outputs included patients receiving at least a single dose of pegzilarginase. For this study, every patient actually received the study treatment to which they were randomized. Hence, primary and secondary efficacy and safety analyses included the full analysis dataset (i.e., all patients randomized and received ≥1 dose).

For the primary efficacy endpoint analysis, missing data were imputed as change from baseline = 0. There was no imputation for missing values for other outcomes. Further information on missing data in the study is provided in Supplementary Information.

Efficacy analyses used a mixed model for repeated measures (MMRM) approach with visit, randomized study treatment, and interaction between visit and randomized treatment as effects as fixed factors, and baseline value included as a covariate. Default covariance structure type was unstructured and if model did not converge, compound symmetry was to be used instead. The Kenward-Roger approximation was used to estimate denominator degrees of freedom. For variables analysed on the log scale, results presented are back transformed to original scale. Significance tests was based on least-squares means using a two-sided α = 0.05 and two-sided 95% confidence intervals. The analyses were implemented using the proc mixed procedure in SAS®.

Primary endpoint and other biochemical outcomes analyses used log-transformed geometric means, analyses of GMFM-E, 2MWT, and GMFM-D used least squares mean estimates. Hochberg multiplicity adjustment for analysis of GMFM-E and 2MWT was used to determine the formal statistical significance of one or both key secondary endpoint analysis comparisons. The procedure was as follows: if both *p*-values (2-sided) were ≤0.05 then both endpoints were declared statistically significant. If not, then if the smallest *p*-value (2-sided) was ≤0.025, then that endpoint alone was declared statistically significant. For other secondary endpoints, no adjustment was made for multiple significance testing. Proportions of patients achieving pArg below guideline level and within normal range were compared using 2-sided Fisher's Exact Test. Other study outcomes were summarized using descriptive statistics.

A prespecified patient-level efficacy analysis (DB period) was conducted among patients evaluable for clinical responses on key functional mobility assessments (i.e., GMFCS Level <4 at baseline, given that no published thresholds for clinically important response at GMFCS Level 4 for GMFM exist, and with data at Week 24). Defined response criteria were normalization of pArg and achievement of applicable clinically important difference thresholds for mobility assessments (Supplementary Table S2).

Statistical programming and analyses were performed using SAS® Version 9.4.

### Role of the funding source

The funder of the study was involved throughout the study, including in study design, data collection, data analysis, data interpretation, and writing of the report.

## Results

### Patients

**Fig. 1:**
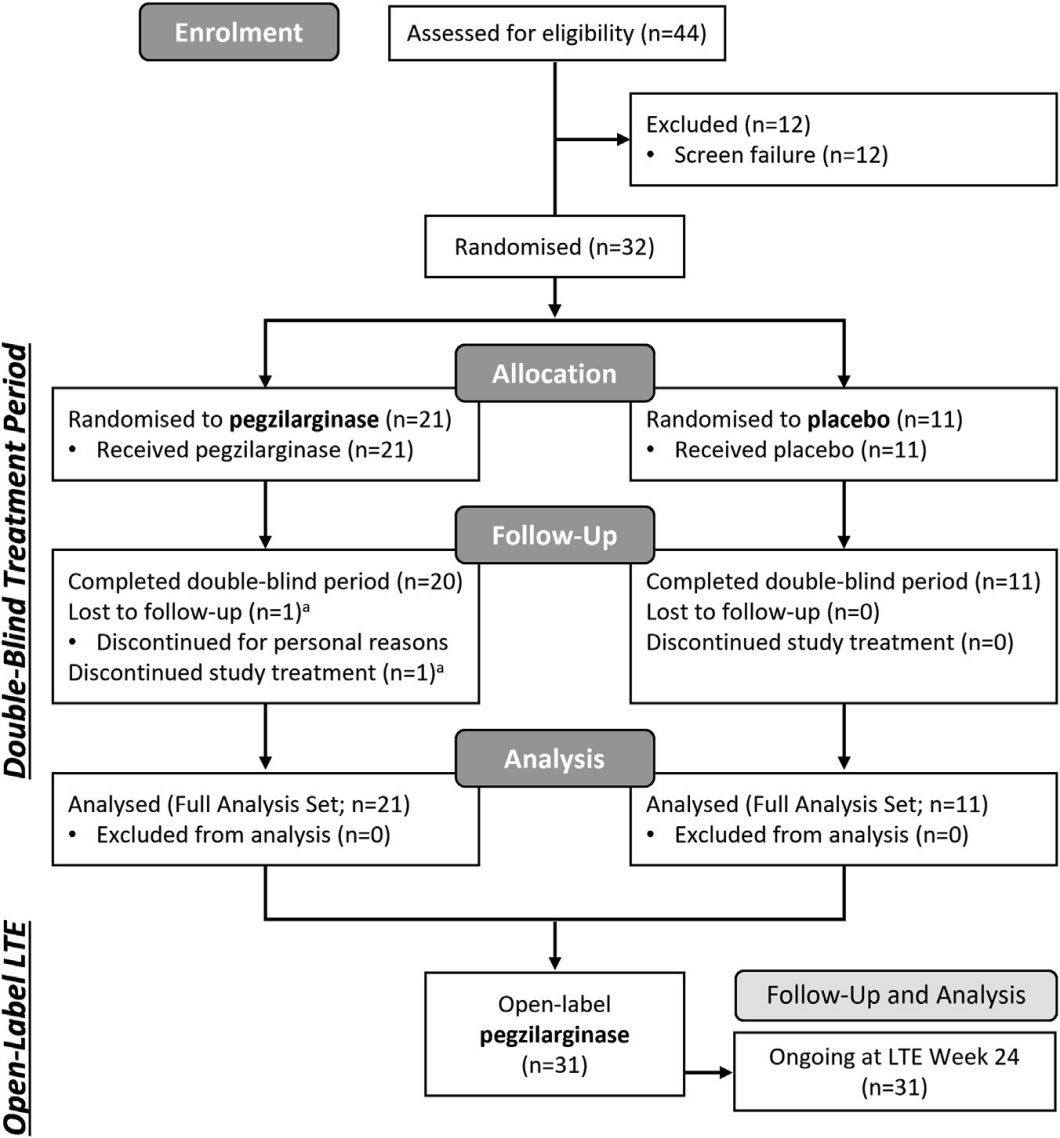
**Patient flow**. One patient randomized to pegzilarginase withdrew consent at Week 6 for personal reasons unrelated to pegzilarginase. All patients who completed the DB period and entered the LTE completed ≥24 weeks in the LTE as of the March 24, 2022 cutoff. Screen failures failed due to meeting the following exclusion criteria: hyperammonaemic history (1), other medical condition judged by investigator to interfere with study assessments (1); or not meeting the following inclusions criteria: provision of informed consent (4), arginine elevation ≥250 μmol/L (2), able to complete the study assessments and had a Baseline deficit in at least 1 component (4). DB, double-blind; LTE, long-term extension. ∗The same patient is reported here as both lost to follow-up and discontinued study treatment.

Of 44 patients screened from 01 May 2019 to 29 March 2021, 32 met study criteria and were randomized to pegzilarginase (n = 21) or placebo (n = 11). One patient (pegzilarginase) discontinued at Week 6 for personal reasons unrelated to treatment. The remaining 31 patients completed the 24-week DB period, continued into the LTE (pegzilarginase/pegzilarginase, n = 20; placebo/pegzilarginase, n = 11), and have completed ≥24 weeks in the LTE as of March 24, 2022 interim data cutoff (Fig. 1).

With a few exceptions (slightly younger age, lower pArg, and less moderate/severe spasticity among patients randomized to pegzilarginase vs placebo), characteristics were generally comparable across groups. Overall, approximately 60% were male; mean (SD) age at enrolment was 10.7 (6.5) years. All patients were managed with dietary protein restriction. Despite standard-of-care treatment, mean (SD) pArg was markedly elevated; 402.0 ± 101.8 μmol/L. Clinical onset occurred at a mean (SD) age of 1.9 ± 2.4 years. Lower-limb spasticity was occurring in 66%, and was moderate or severe in 38%; upper-limb spasticity was present in 12%. More than 50% had gross motor functional impairment of GMFCS Level ≥2 (Table 1).

**Table 1:** Key demographics, clinical characteristics, and baseline assessments (full analysis set).

Patient information	Pegzilarginase (n = 21)	Placebo (n = 11)	Overall (n = 32)
**Age at enrollment, y**			
Mean ± SD	9.6 ± 6.16	12.9 ± 6.77	10.7 ± 6.47
Median (Q1, Q3)	8.0 (6.0, 14.0)	12.0 (8.0, 14.0)	10.5 (6.0, 14.0)
Min, max	2, 28	5, 29	2, 29
**Sex, n (%)**			
Male	12 (57.1)	7 (63.6)	19 (59.4)
Female	9 (42.9)	4 (36.4)	13 (40.6)
**Race, n (%)**			
White/Caucasian descent	10 (47.6)	4 (36.4)	14 (43.8)
Asian	3 (14.3)	3 (27.3)	6 (18.8)
Black/African descent	0	2 (18.2)	2 (6.3)
Multiple race	1 (4.8)	1 (9.1)	2 (6.3)
Other	6 (28.6)	0	6 (18.8)
Missing	1 (4.8)	1 (9.1)	2 (6.3)
**Region, n (%)**			
US	8 (38.1)	6 (54.5)	14 (43.8)
Non-US	13 (61.9)	5 (45.5)	18 (56.3)
**Country, n (%)**			
Austria	2 (9.5)	0 (0.0)	2 (6.3)
Canada	1 (4.8)	0 (0.0)	1 (3.1)
France	4 (19.0)	2 (18.2)	6 (18.8)
Germany	1 (4.8)	0 (0.0)	1 (3.1)
Italy	2 (9.5)	1 (9.1)	3 (9.4)
United Kingdom	3 (14.3)	2 (18.2)	5 (15.6)
United States of America	8 (38.1)	6 (54.5)	14 (43.8)
**Age at onset of manifestations,**^a^**y**			
Mean ± SD	1.6 ± 2.5	2.5 ± 2.0	1.9 ± 2.4
Median (Q1, Q3)	1.0 (0.0, 2.0)	2.0 (1.0, 3.0)	1.0 (0.0, 3.0)
Min, max	1, 10	0, 7	0, 10
**Age at diagnosis,**^a^**y**			
Mean ± SD	2.8 ± 4.1	4.2 ± 3.1	3.3 ± 3.8
Median (Q1, Q3)	0.7 (0.1, 4.2)	4.6 (1.7, 4.9)	2.6 (0.1, 4.8)
Min, max	0, 15	0, 11	0, 15
**Baseline plasma arginine, μmol/L**^b^			
Mean ± SD	365.4 ± 93.7	471.7 ± 79.9	402.0 ± 101.8
Median (Q1, Q3)	368.2 (292.0, 421.0)	483.7 (434.0, 517.0)	398.2 (317.6, 482.6)
Min, max	202, 572	294, 573	202, 573
**Key medical history, n (%)**			
*Spasticity*			
Any	13 (61.9)	8 (72.7)	21 (65.6)
Lower-limb	13 (61.9)	8 (72.7)	21 (65.6)
Upper limb	1 (4.8)	3 (27.3)	4 (12.5)
Moderate to severe	6 (28.6)	6 (54.5)	12 (37.5)
*Seizures*	7 (33.3)	4 (36.4)	11 (34.4)
*Liver test abnormalities*	14 (66.7)	9 (81.8)	23 (71.9)
*Hyperammonemia*	12 (57.1)	6 (54.5)	18 (56.3)
**GMFCS Level,**^c^**n (%)**			
Level 1	9 (42.9)	5 (45.5)	14 (43.8)
Level 2	9 (42.9)	4 (36.4)	13 (40.6)
Level 3	0	0	0
Level 4	3 (14.3)	2 (18.2)	5 (15.6)
**BaselineGMFM-Escore,**^d^**points**			
Mean ± SD	48.3 ± 19.9	46.5 ± 24.6	47.7 ± 21.2
Median (Q1, Q3)	53.0 (41.0, 66.0)	56.0 (35.0, 69.0)	54 (37.5, 66.0)
Min, max	5, 71	0, 72	0, 72
**Baseline 2MWT distance,**^e^**m**			
Mean ± SD	109.0 ± 55.7	99.9 ± 49.0	105.8 ± 52.8
Median (Q1, Q3)	122.0 (59.5, 149.5)	102.0 (70.0, 148.0)	118 (69.0, 148.0)
Min, max	2, 202	0, 171	0, 202
**BaselineGMFM-Dscore, points**^f^			
Mean ± SD	28.0 ± 9.6	29.5 ± 12.4	28.5 ± 10.4
Median (Q1, Q3)	30.0 (24.0, 34.0)	33.0 (30.0, 38.0)	32 (24.0, 37.0)
Min, max	1, 38	0, 39	0, 39

2MWT, 2-min walk test; GMFCS, Gross Motor Function Classification System; GMFM-D, Gross Motor Function Measure part D (possible score range, 0–39); GMFM-E, Gross Motor Function Measure part E (possible score range, 0–72), m, meters; Q, quartile; UK, United Kingdom; US, United States of America; y, years.

a Age at onset of manifestations available for 21 patients (pegzilarginase, n = 11; placebo, n = 10); age at diagnosis available for 26 patients (pegzilarginase, n = 17; placebo, n = 9).

b One patient had plasma arginine <250 μmol/L (screening, 242 μmol/L; baseline, 202 μmol/L) but was considered eligible for the study based on documented historical plasma arginine levels.

c No patients at GMFCS Level 5 were enrolled due to inability to complete functional mobility assessments.

d Baseline GMFM-E was assessed in 10/11 patients in the placebo group; 1 patient was not assessed at baseline because of severe disability and wheelchair dependence.

e Baseline 2MWT was assessed in 20/21 patients in the pegzilarginase group; 1 patient was not assessed at baseline due to young age.

f Excludes one patient (placebo) with missing baseline value.

### Efficacy

#### Double-blind period

**Fig. 2:**
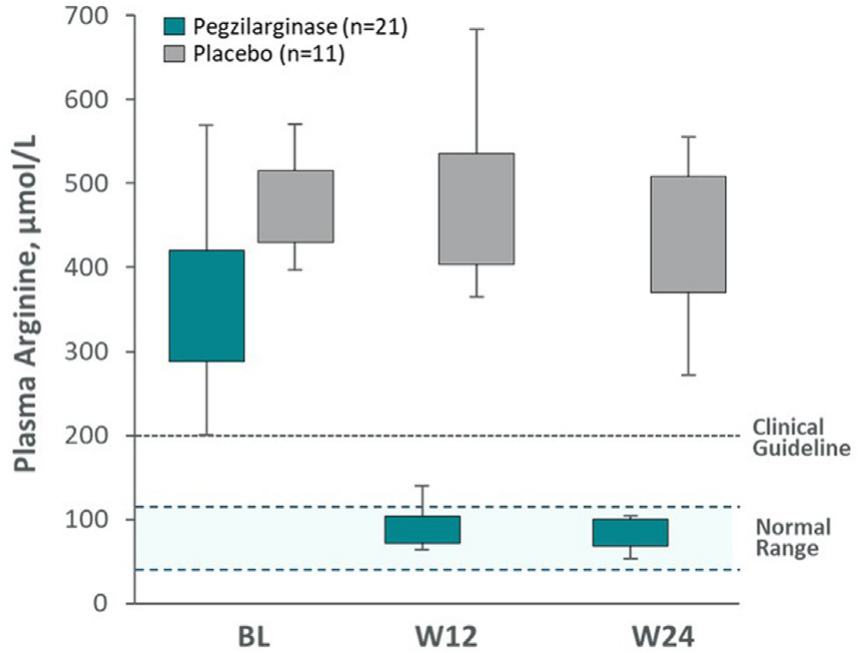
**Effect of pegzilarginase on plasma arginine levels (primary endpoint; full analysis set)**. Pegzilarginase statistically significantly reduced mean plasma arginine levels by 76.7% comparing to placebo at Week 24 (95% CI: −67.1%, −83.5%; *p* < 0.0001). Boxes represent middle 50%; error bars represent 95% CIs. Statistical significance was based on geometric means with any missing post-baseline values imputed as change from baseline = 0. Normal range for plasma arginine is 40–115 μmol/L.^4^ BL, baseline; W, week.

In the DB period (primary analysis), pegzilarginase statistically significantly reduced geometric mean (CV) baseline pArg from 354.0 (0.27) μmol/L to 86.4 (0.50) μmol/L at Week 24; a 76.7% reduction compared to placebo (95% CI: −67.1%, −83.5%; *p* < 0.0001; Fig. 2). There were no meaningful changes in pArg with placebo (baseline, 464.7 (0.19) μmol/L; Week 24, 426.5 (0.27) μmol/L). In the pegzilarginase arm, mean pArg was well below guideline-recommended levels (<200 μmol/L) and within normal range (40–115 μmol/L) by Week 12. No patients receiving placebo achieved these levels. At Week 24, pArg <200 μmol/L was achieved by 90.5% on pegzilarginase, and normalization by 90.5% (all *p* < 0.0001 vs placebo).

Key secondary outcomes showed numeric improvements in functional mobility with pegzilarginase at Week 24. At baseline, mean GMFM-E scores were 48.3 points and 46.5 points (possible maximum: 72 points) in the pegzilarginase and placebo arms, respectively (Table 1). At Week 24, GMFM-E score was increased by 4.2 points with pegzilarginase and decreased by 0.4 point with placebo (LS mean difference 4.6 points; 95% CI: −1.1, 10.2). Changes from baseline in the pegzilarginase arm exceeded the thresholds for clinically important response, demonstrating clinically meaningful improvement. 2MWT distance at baseline was 109.0 m and 99.0 m in the pegzilarginase and placebo arms, respectively (Table 1). At Week 24, distance was increased by 7.3 m (12.8%) with pegzilarginase vs 2.7 m (4.1%) with placebo (LS mean difference 5.5 m; 95% CI: −15.6, 26.7). Also for 2MWT, changes from baseline in the pegzilarginase arm exceeded the thresholds for clinically important response, demonstrating clinically meaningful improvement.

Mobility function improvements were more pronounced in patients at GMFCS Level ≥2 than Level 1 (with fewer baseline deficits and thus scores at or close to the ceiling of assessments, limiting the ability of the tool to measure improvement). However, many of these patients improved to the upper limit or maintained their status without decline. For GMFM-E, GMFCS ≥2 patients demonstrated a mean (SD) change increase from baseline at Week 24 of 5.1 (9.43) on pegzilarginase and decreased 0.8 (8.42) on placebo; LS mean difference (95% CI) between groups favoured pegzilarginase by 5.5 points (−4.7, 15.8) (Supplementary Figure S1). Similarly, for 2MWT, improvement was more pronounced at GMFCS Level ≥2 (9.4 m [35.82]) than GMFCS Level 1 (3.7 m [20.99]; LS mean difference (95% CI) between treatment groups favoured pegzilarginase by 10.6 m [−26.4, 47.7]) (Supplementary Figure S1).

Other secondary outcomes demonstrated additional biochemical and functional mobility improvements (Table 2). Statistically significant and clinically meaningful improvement in GMFM-D with pegzilarginase at Week 24 (Table 2; Fig. 4) was demonstrated. At baseline, GMFM-D score was 28.0 and 29.5 points in pegzilarginase and placebo arms, respectively (possible maximum: 39 points). The increase from baseline with pegzilarginase vs placebo was 2.3 points (LS mean difference; 95% CI: 0.4, 4.2; *p* = 0.0208). Plasma levels of GCs statistically significantly decreased from baseline with pegzilarginase; relative reductions vs placebo in argininic acid, guanidinoacetic acid, α-keto-δ-guanidinovaleric acid, and alpha-N-acetylarginine range from 53.3% (95% CI: −32.2%, −67.8%, *p* = 0.0003) to 69.8% (95% CI: −51.8%, −81.1%, *p* < 0.0001) with demonstrated strong correlations with pArg. Plasma ornithine statistically significantly increased from baseline with pegzilarginase (change from baseline: +106.9% vs placebo [95% CI: 1.567, 2.731; *p* < 0.0001]). Correlation analyses at Week 24 revealed statistically significant direct relationships between levels of pArg and individual GCs (Pearson correlation coefficient: 0.774 [*p* < 0.0001]–0.602 [*p* = 0.0003]), and statistically significant inverse relationship between pArg and ornithine (Pearson correlation coefficient: −0.508 [*p* = 0.0096]) (Supplementary Table S1).

**Table 2:** Secondary efficacy outcomes in the 24-week double-blind period (full analysis set).

Endpoint^a^	Normal biochemical ranges	Pegzilarginase (n = 21)		Placebo (n = 11)		Change from baseline relative to placebo (95% CI)^b^	*p*-value
		Baseline	Week 24	Baseline	Week 24		
**Primary**							
pArg, μmol/L	40–115	354.0 (0.27)	86.4 (0.50)	464.7 (0.19)	426.5 (0.27)	−76.7% (−67.1%, −83.5%)	<0.0001
**Key secondary**							
2MWT, meters	NA	109.0 ± 55.7	115.9 ± 51.8	99.0 ± 49.0	102.3 ± 51.1	+5.5 (−15.6, 26.7)	NA^g^
GMFM-E, points^d^	NA	48.3 ± 19.9	52.0 ± 21.3	46.5 ± 24.6	46.1 ± 25.7	+4.6 (−1.1, 10.2)	NA^g^
**Other secondary**							
GMFM-D, points^e^^,^^f^	NA	28.0 ± 9.6	30.5 ± 10.1	29.5 ± 12.4	28.2 ± 13.3	+2.3 (−0.4, 4.2)	0.0208
ARGA, μmol/L	0.025–0.1	2.4 (0.45)	0.7 (0.65)	3.2 (0.42)	3.2 (0.40)	−69.5% (−57.2%, −78.3%)	<0.0001
GAA, μmol/L	0.4–3.0	3.4 (0.70)	1.7 (0.50)	3.6 (0.52)	3.7 (0.51)	−53.3% (−32.2%, −67.8%)	0.0003
GVA, μmol/L	<0.05	4.5 (0.49)	1.3 (0.67)	5.4 (0.47)	4.8 (0.42)	−68.3% (−54.6%, −77.9%)	<0.0001
NAARG, μmol/L	0.025–0.255	1.0 (0.74)	0.3 (0.87)	1.5 (0.71)	1.3 (0.59)	−69.8% (−51.8%, −81.1%)	<0.0001
ORN, μmol/L	c	38.6 ± 1.6	67.7 ± 1.4	30.6 ± 1.2	32.8 ± 1.2	+106.9% (56.7%, 173.1%)	<0.0001

2MWT, 2-min walk test; ARGA, argininic acid; GAA, guanidinoacetic acid; GMFM-D, Gross Motor Function Measure part D; GMFM-E, Gross Motor Function Measure part E; GVA, α-keto-δ-guanidinovaleric acid; NA, not applicable; NAARG, α-N-acetylarginine; ORN, ornithine; pArg, plasma arginine.

a Arginine, guanidino compounds and ornithine are presented as geometric mean (CV); GMFM-E, GMFM-D and 2MWT are presented as mean ± SD.

b Guanidino compounds and ornithine are presented as estimated percentage change from baseline relative to placebo; GMFM-E, GMFM-D and 2MWT are presented as least squares mean difference vs placebo. Statistical significance is based on mixed model for repeated measures.

c Normal range for ornithine is age-dependent: ages 2–17 years, 22–97 μmol/L; ages ≥18 years, 38–130 μmol/L.^30^

d Possible score range, 0–72 points; lower scores indicate greater functional mobility impairment.

e Possible score range, 0–39 points; lower scores indicate greater functional mobility impairment.

f Excludes one patient (placebo) with missing baseline value.

g Not reported as per hierarchical testing.

**Fig. 3:**
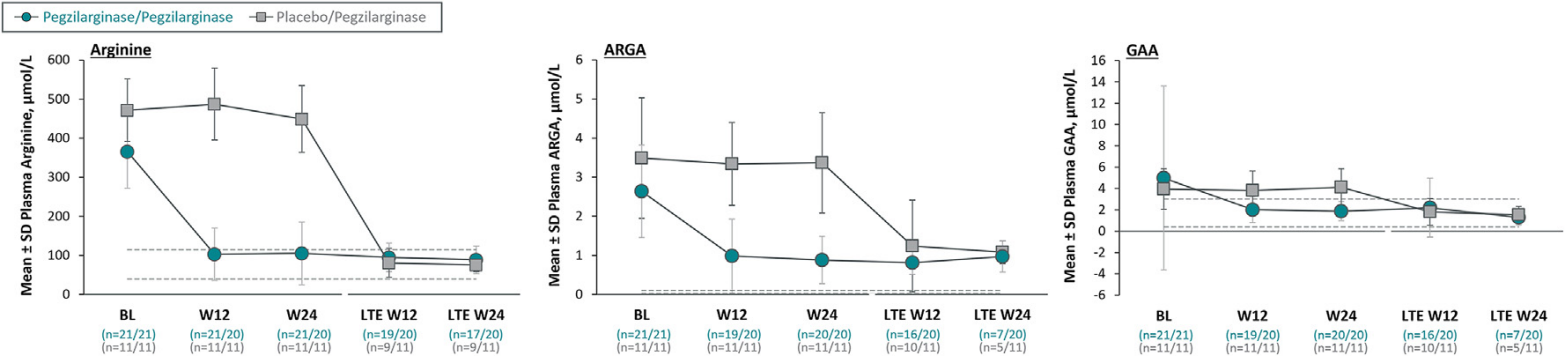

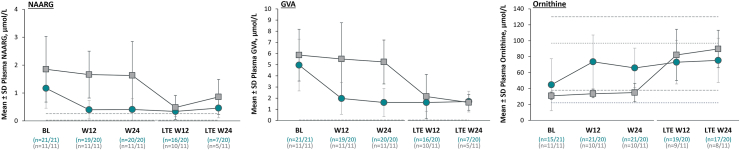
**Sustained efficacy on key biochemical endpoints with long-term pegzilarginase (full analysis set)**. Group sizes reflect all patients with data at each time point; there was no imputation for missing values. Normal range for plasma arginine is 40–115 μmol/L.^4^ Normal ranges for guanidino compounds are: ARGA, 0.025–0.1 μmol/L (dashed lines); GAA, 0.4–3.0 μmol/L (dashed lines); GVA, <0.05 μmol/L (dashed lines); NAARG, 0.025–0.255 μmol/L (dashed lines).^31^ Normal range for ornithine is age-dependent: ages 2–17 years, 22–97 μmol/L (dotted lines); ages ≥18 years, 38–130 μmol/L (dashed lines).^30^ ARGA, argininic acid; BL, baseline; GAA, guanidinoacetic acid; GMFM-D, Gross Motor Function Measure part D; GVA, α-keto-δ-guanidinovaleric acid; LTE, long-term extension; NAARG, α-N-acetylarginine; W, week.

**Fig. 4:**
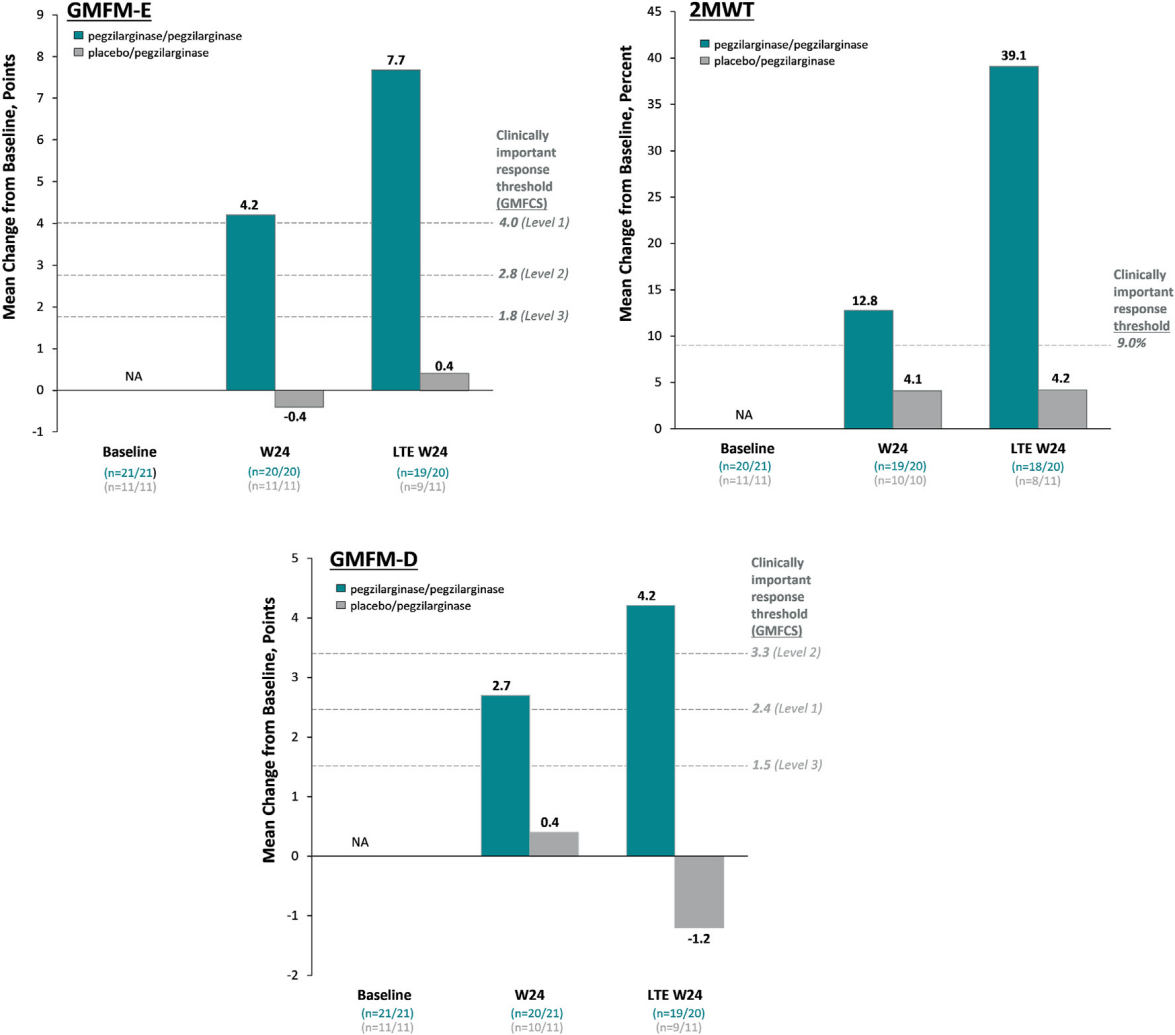
**Sustained effect of pegzilarginase on functional mobility assessments (full analysis set)**. Group sizes reflect all patients with data at each time point; there was no imputation for missing values. 2MWT, 2-min walk test; BL, baseline; GMFCS, Gross Motor Function Classification System; GMFM-D, Gross Motor Function Measure part D; GMFM-E, Gross Motor Function Measure part E; LTE, long-term extension; W, week.

In addition, although sites were instructed to minimize dietary protein prescription changes, a higher proportion of patients on pegzilarginase (38.1%) than placebo (18.2%) consumed >15% of total calories/day at Week 24 vs baseline. Similar was noted for total consumed protein (including natural and EAA protein); 42.9% on pegzilarginase compared to 18.2% on placebo consumed >15% of total protein. Importantly, dietary excursions did not impact ability to maintain pArg within therapeutic range.

**Fig. 5:**
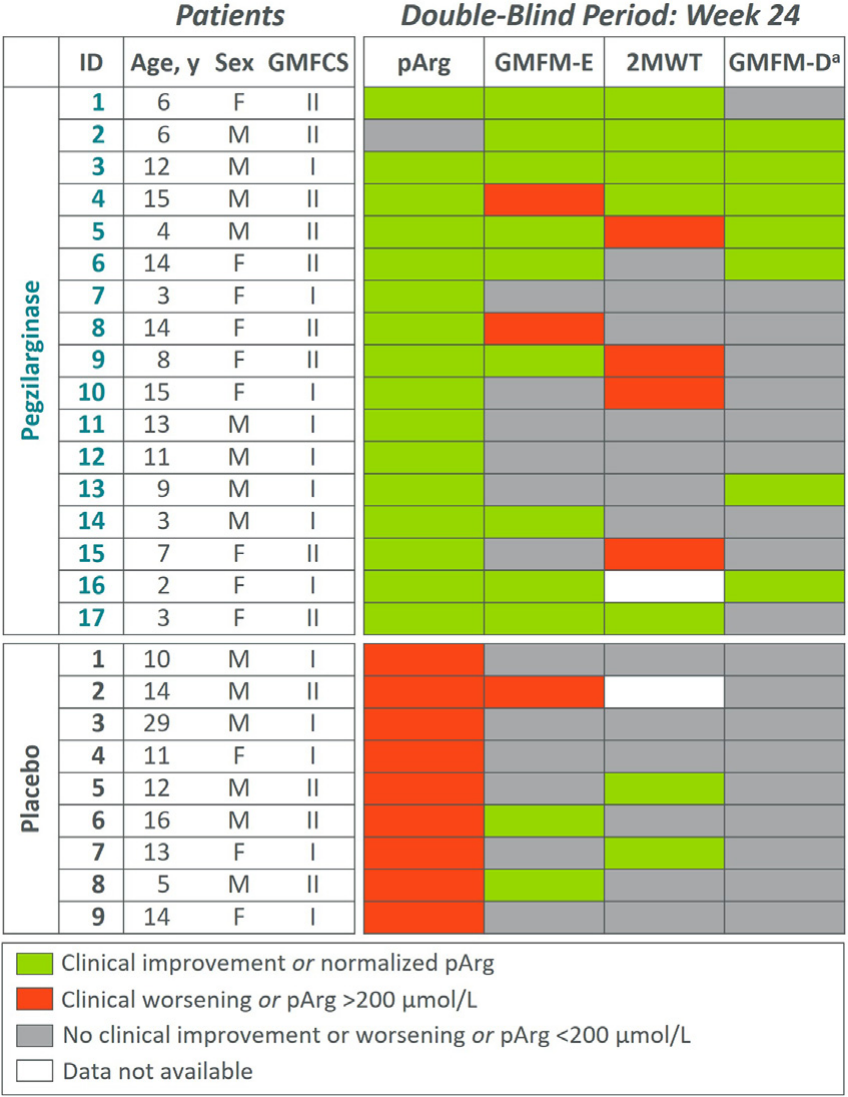
**Patient-level analysis of pegzilarginase effect on plasma arginine and clinical responses**. Analysis includes patients with baseline GMFCS Level <4 and with data at Week 24. Patients with GMFCS Level 4 were excluded due to lack of defined thresholds for clinical response for GMFM-E and GMFM-D. 2MWT, 2-min walk test; GMFCS, Gross Motor Function Classification System; GMFM-D, Gross Motor Function Measure part D; GMFM-E, Gross Motor Function Measure part E; pArg, plasma arginine; LTE, long-term extension.

For patient-level analysis, 26 patients were eligible; 5 were excluded because of baseline GMFCS Level 4 (pegzilarginase, n = 3; placebo, n = 2) and 1 (pegzilarginase) because of withdrawal before Week 24. Among these patients (pegzilarginase, n = 17/21; placebo, n = 9/11), important differences were observed for normalization of pArg and clinical responses on functional mobility assessments at Week 24 (Fig. 5). In the pegzilarginase arm, 94% (n = 16/17) had normalized pArg and all 17 were below guideline-recommended level (<200 μmol/L). In contrast, all 9 placebo patients remained >200 μmol/L. At Week 24, predefined clinical response criteria for ≥2 functional mobility assessments were met by 47% of pegzilarginase treated (n = 8/17) versus none receiving placebo. With pegzilarginase, 35% (n = 6/17) met thresholds for clinically important response for ≥2 mobility assessments with no worsening on other endpoints. The magnitude of change was greater in the pegzilarginase arm vs placebo. Three patients on pegzilarginase achieved or exceeded age- and sex-matched norms^28^ on 2MWT. Several patients achieved maximum possible score for GMFM-E and/or GMFM-D but could not achieve clinical response criteria because scores at baseline were near the maximum score, precluding an improvement to thresholds for clinically important difference.

#### Long-term extension

In the LTE, patients continuing pegzilarginase (pegzilarginase/pegzilarginase, n = 20) maintained mean pArg within normal range through 48 weeks of total treatment (mean level at LTE Week 24: 89.0 ± 35.2 μmol/L) (Fig. 3). Patients initially randomized to placebo and transitioned to pegzilarginase (placebo/pegzilarginase, n = 11) mirrored the pArg reduction in the DB pegzilarginase arm (mean pArg level at LTE Week 24: 75.2 ± 16.2 μmol/L versus 448.8 ± 85.8 μmol/L at end of DB period). Among patients with pArg data at LTE Week 24 (n = 28; both arms), all were <200 μmol/L and nearly all within normal range (pegzilarginase/pegzilarginase, 88% [n = 15/17]; placebo/pegzilarginase, 82% [n = 9/11]). Decreased levels of GCs and increased ornithine were also observed during LTE follow-up in all patients (Fig. 3).

Meaningful improvements in mean GMFM-E, 2MWT, and GMFM-D continued through 48 weeks in the pegzilarginase/pegzilarginase arm (Fig. 4). At LTE Week 24, mean GMFM-E score increased by 7.7 points (vs 4.2 points at Week 24 in the DB period), 2MWT distance increased by 39% (22.8 m) (vs 13% [7.3 m] at Week 24), and GMFM-D increased by 4.2 points (vs 2.7 points at Week 24). For all 3 clinical outcomes, mean improvements in the pegzilarginase/pegzilarginase arm exceeded thresholds for clinically important response. The mean GMFM-E and GMFM-D changes from baseline in the placebo/pegzilarginase arm did not exceed thresholds for response, though there was numeric increase from baseline at LTE Week 24 for GMFM-E.

### Exposure, safety, and tolerability

#### Treatment exposure

All 31 patients who completed the DB period also completed ≥24 weeks in the LTE and switched from IV to SC administration. Exposure and dosing compliance (doses received vs expected) are presented in Table 3 (DB) and Table 4 (LTE). Patients in the pegzilarginase/pegzilarginase arm had a total median exposure of 78 weeks (range 6–121 weeks, reflecting the Week 6 withdrawal). The placebo/pegzilarginase patients received pegzilarginase for a median duration of 53 weeks (range 24–125 weeks). Dosing compliance was high (mean overall compliance rate >93%). No doses were missed because of safety or tolerability.

**Table 3:** Summary of key safety observations in the 24-week double-blind period (full analysis set).

Exposure and AEs	Pegzilarginase (n = 21)	Placebo (n = 11)	Risk difference % (95% CI; *p*-value)
*Exposure and compliance*			
**Treatment exposure, weeks**			
Mean ± SD	23.1 ± 3.9	23.8 ± 0.75	–
Median (range)	24.0 (6–25)^a^	24.0 (22–25)	–
**Dosing compliance,**^b^**%**			
Mean ± SD	94.1 ± 7.1	97.7 ± 2.9	–
Median (range)	95.8 (71–100)	100.0 (92–100)	–
*Summary of AEs, n (%)*			
**Any treatment-emergent AE**	**18 (85.7)**	**11 (100.0)**	**−11.74 (−30.75, 7.27; *p*** = **0.05343)**
Mild	10 (47.6)	5 (45.5)	2.16 (−34.20, 38.53; *p* = 1.0000)
Moderate	7 (33.3)	6 (54.5)	−21.21 (−56.88, 14.46; *p* = 0.2826)
Severe	1 (4.8)	0	2.65 (−12.80, 18.10; *p* = 1.0000)
**AE leading to discontinuation**	**0**	**0**	–
**AE leading to dose reduction**	**0**	**0**	–
**AEs of special interest**			
Hypersensitivity reaction	2 (9.5)	0	2.73 (0.14, 52.30; *p* = 005343)
Injection site reaction	0	0	–
Hyperammonaemic episode^c^	3 (14.3)	4 (36.4)	−22.08 (−54.20, 10.05; *p* = 0.1967)
**Serious AEs**	**4 (19.0)**	**4 (36.4)**	**−17.32 (−50.33, 15.70; *p*** = **0.3970)**
Leading to fatal outcome	0	0	–
Related to pegzilarginase	1 (4.8)	0	2.65 (−12.80, 18.10; *p* = 1.0000)
**AEs with ≥15% incidence in either treatment arm, n (%)**			
Vomiting	6 (28.6)	3 (27.3)	1.30 (−31.35, 33.95; *p* = 1.0000)
Pyrexia	4 (19.0)	0	16.29 (−4.01, 36.58; *p* = 0.2720)
Cough	4 (19.0)	1 (9.1)	9.96 (−13.93, 33.85; *p* = 0.6367)
Ammonia increased	3 (14.3)	2 (18.2)	−3.90 (−31.16, 23.37; *p* = 1.0000)
Hyperammonaemia	2 (9.5)	3 (27.3)	−17.75 (−46.91, 11.41; *p* = 0.3098)
Nausea	1 (4.8)	4 (36.4)	−31.60 (−61.45, −1.75; *p* = 0.0367)
Abdominal pain	1 (4.8)	3 (27.3)	−22.51 (−50.36, 5.34; *p* = 0.1055)
Decreased appetite	0	2 (18.2)	−18.56 (−42.37, 5.25; *p* = 0.1109)

AE, adverse event. For risk difference, Wald confidence intervals were used.

a One patient randomized to pegzilarginase withdrew the study at Week 6 for personal reasons unrelated to study treatment.

b Patients with temporary pauses due to the COVID-19 pandemic were allowed to restart the study. The number of expected doses in compliance calculations includes COVID-19 pauses.

c Hyperammonemic episodes were identified through a targeted search for preferred terms of hyperammonaemia, hyperammonaemic crisis, and hyperammonaemic encephalopathy; no events of hyperammonaemic crisis occurred in the study.

**Table 4:** Summary of key safety observations in the long-term extension (interim analysis; full analysis set)^a^.

Exposure and AEs	Pegzilarginase/pegzilarginase (n = 20)	Placebo/pegzilarginase (n = 11)	Both arms (n = 31)	Risk difference % (95% CI; *p*-value)
*Exposure and Compliance*				
**Treatment exposure, weeks**				
Mean ± SD	51.7 ± 21.2	58.6 ± 34.8	54.1 ± 26.4	–
Median (range)	54.5 (24–97)	53.0 (24–125)	54.0 (24–125)	–
**Dosing compliance,**^b^**%**				
Mean ± SD	94.2 ± 4.8	91.5 ± 10.4	93.2 ± 7.2	–
Median (range)	94.1 (84–100)	91.8 (62–100)	93.9 (62–100)	–
*Summary of AEs, n (%)*				
**Anytreatment-emergentAE**	**19 (95.0)**	**11 (100.0)**	**30 (96.8)**	**−2.98 (−18.76, 12.81;*****p*** **= 1.0000)**
Mild	8 (40.0)	4 (36.4)	12 (38.7)	3.64 (−31.99, 39.26; *p* = 1.0000)
Moderate	11 (55.0)	4 (36.4)	15 (48.4)	18.64 (−17.19, 54.46; *p* = 0.4578)
Severe	0	3 (27.3)	3 (9.7)	−26.79 (−53.32, −0.25; *p* = 0.0367)
**AE leading to discontinuation**	**0**	**0**	**0**	–
**AE leading to dose reduction**	**1 (5.0)**	**0**	**1 (3.2)**	**2.98 (−12.81, 18.76;*****p*** **= 1.0000)**
**AEs of special interest**				
Hypersensitivity reaction	0	0	0	–
Injection site reaction	2 (10.0)	0	2 (6.5)	7.74 (−10.14, 25.62; *p* = 0.5269).
Hyperammonaemic episode^c^	2 (10.0)	5 (45.5)	7 (22.6)	−35.45 (−67.68, −3.23; *p* = 0.0665)
**Serious AEs**	**5 (25.0)**	**7 (63.6)**	**12 (38.7)**	**−38.64 (−72.82, −4.46;*****p*** **= 0.0564)**
Leading to fatal outcome	0	0	0	–
Related to pegzilarginase	0	1 (9.1)	1 (3.2)	−10.12 (−29.93, 9.70; *p* = 0.3548)
**AEs with ≥15% incidence in either treatment arm, n (%)**				
Vomiting	7 (35.0)	6 (54.5)	13 (41.9)	−21.21 (−56.88, 14.46; *p* = 0.2826)
Pyrexia	6 (30.0)	3 (27.3)	9 (29.0)	1.30 (−31.35, 33.95; *p* = 1.0000)
Fatigue	6 (30.0)	2 (18.2)	8 (25.8)	10.39 (−19.49, 40.27; *p* = 0.6808)
Alanine aminotransferase increased	5 (25.0)	2 (18.2)	7 (22.6)	5.63 (−23.55, 34.81; *p* = 1.0000)
Aspartate aminotransferase increased	5 (25.0)	2 (18.2)	7 (22.6)	5.63 (−23.55, 34.81; *p* = 1.0000)
SARS-CoV-2 test positive^d^	4 (20.0)	3 (27.3)	7 (22.6)	−8.23 (−39.45, 23.00; *p* = 0.6675)
Nausea	4 (20.0)	2 (18.2)	6 (19.4)	0.84 (−27.45, 29.18; *p* = 1.0000)
Ammonia increased	3 (15.0)	4 (36.4)	7 (22.6)	−22.08 (−54.20, 10.05; *p* = 0.1967)
COVID-19	3 (15.0)	2 (18.2)	5 (16.1)	−3.90 (−31.16, 23.37; *p* = 1.0000)
Amino acid level increased	3 (15.0)	2 (18.2)	5 (16.1)	−3.90 (−31.16, 23.37; *p* = 1.0000)
Gastroenteritis	3 (15.0)	0	3 (9.7)	11.74 (−7.27, 30.75; *p* = 0.5343)
Upper respiratory tract infection	3 (15.0)	0	3 (9.7)	11.74 (−7.27, 30.75; *p* = 0.5343)
Rhinorrhoea	3 (15.0)	0	3 (9.7)	11.74 (−7.27, 30.75; *p* = 0.5343)
Hyperammonaemia	2 (10.0)	5 (45.5)	7 (22.6)	−35.93 (−67.92, −3.94; *p* = 0.0318)
Abdominal pain	2 (10.0)	3 (27.3)	5 (16.1)	−17.75 (−46.91, 11.41; *p* = 0.3098)
Headache	2 (10.0)	2 (18.2)	4 (12.9)	−8.66 (−34.68, 17.36; *p* = 0.5932)
Oropharyngeal pain	2 (10.0)	2 (18.2)	4 (12.9)	−8.66 (−34.68, 17.36; *p* = 0.5932)

AE, adverse event. For risk difference, Wald confidence intervals were used.

a All patients received pegzilarginase for ≥24 weeks in the LTE, in addition to the 24-week double-blind period, as of the interim cutoff date of March 24, 2022.

b Patients with temporary pauses due to the COVID-19 pandemic were allowed to restart the study. The number of expected doses in compliance calculations includes COVID-19 pauses.

c Hyperammonaemic episodes were identified through a targeted search for preferred terms of hyperammonaemia, hyperammonemic crisis, and hyperammonaemic encephalopathy; no events of hyperammonaemic crisis occurred in the study.

d Patients with a positive SARS-CoV-2 test are distinct from those with COVID-19 illness.

#### Adverse events

Pegzilarginase was well-tolerated, with AEs being mostly transient, mild/moderate in severity, and either self-limiting or manageable with standard medical care. Common AEs (≥15% of patients in either arm) are summarized in Tables 3 and 4 for the DB and LTE periods, respectively.

In the DB period, AEs were reported for 86% of patients on pegzilarginase and 100% on placebo; none led to discontinuation or dose reduction (Table 3). Most commonly reported AEs on pegzilarginase were vomiting (29% [n = 6/21]), cough (19% [n = 4/21]), and pyrexia (19% [n = 4/21]) and on placebo, nausea (36% [n = 4/11]), hyperammonaemia, vomiting, and abdominal pain (each 27% [n = 3/11]). Serious AEs were reported for 19% on pegzilarginase (n = 4/21) and 36% on placebo (n = 4/11), and consisted of hyperammonaemia (pegzilarginase, 10% [n = 2/21] vs placebo, 27% [n = 3/11]), hyperammonaemic encephalopathy (5% [n = 1/21] vs 9% [n = 1/11]), and vomiting (5% [n = 1/21], pegzilarginase only). The event of hyperammonaemic encephalopathy in the pegzilarginase arm occurred during concurrent urinary tract infection and constipation, was moderate in severity, not life-threatening, and resolved in one week. There were no notable adverse trends in liver function tests or other laboratory assessments. A similar safety profile was observed during LTE compared with the DB period. During LTE, AEs were reported for 97% overall (n = 30/31), none leading to discontinuation (Table 4).

AESIs reported during the DB and LTE periods are summarized in Tables 3 and 4. During DB period, 10% (n = 2/21) on pegzilarginase (IV infusion) experienced nonserious, mild/moderate HSRs that were managed with antihistamines. These occurred within first 8 weeks of dosing. No additional HSRs occurred thereafter. ISRs were reported for 6% (n = 2/31) during LTE (SC injection). All were mild and resolved without dose change. Hyperammonaemic episodes were reported for 14% (n = 3/21) on pegzilarginase and 36% (n = 4/11) on placebo in the DB period, and for 23% (n = 7/31) during LTE.

#### Anti-drug antibodies

Transient, generally low-titre ADAs were detected in both treatment arms during DB period (pegzilarginase, 19% [n = 4/21]; placebo, 27% [n = 3/11]), including 1 patient in each arm with pre-existing ADAs at baseline. One patient per arm was positive for both anti-PEG and anti-pegzilarginase ADAs; others only for anti-pegzilarginase ADAs. No patients on pegzilarginase in the DB period developed ADAs during the LTE. The 3 ADA-positive patients in the placebo arm were positive at the end of the DB period before first dose of pegzilarginase, and a single placebo/pegzilarginase patient developed ADAs after first pegzilarginase dose. There was no sustained biochemical or meaningful clinical impact of ADAs, and irrespective of DB period treatment, no previously ADA-negative patient developed ADAs after switching from IV to SC dosing.

## Discussion

ARG1-D is a progressive and debilitating disorder with an urgent unmet need for effective treatment that maintains adequate reduction of pArg levels and prevent clinical deterioration. In this study, all patients were managed with dietary protein restriction at baseline, but their pArg levels were markedly elevated, at an average 3.5-fold the upper limit of normal (and twice the current management guidelines level). Progression despite standard-of-care management was evident at baseline, with majority of patients having documented lower-limb spasticity and all having established deficits of gross motor function. Pegzilarginase statistically significantly reduced pArg levels, surpassing guideline-recommended levels, and achieved and maintained normal levels through 48 weeks of treatment. Further, increases from baseline in multiple assessments of functional mobility with pegzilarginase met or exceeded the applied thresholds for clinically important improvements, suggesting clinically meaningful improvements. Importantly, these improvements continued to increase in magnitude with long-term treatment in this progressive disorder. Pegzilarginase was well-tolerated, with a safety profile consistent with previous studies,^21^^,^^22^ and no new safety signals identified.

Management of ARG1-D is focused on achieving and maintaining pArg sufficiently low to prevent or decrease progression.^12^ Pegzilarginase demonstrated rapid, statistically significant, and sustained efficacy on pArg levels. In the DB period, pegzilarginase significantly decreased pArg providing normalized levels. This reduction was maintained through 48 weeks of treatment irrespective of route of administration. Normalization of pArg was previously unachievable with current standard-of-care, as reflected by the multiple-fold elevation of pArg in the trial cohort at baseline and extensive documentation in the literature.^1^^,^^3^^,^^6^^,^^9^^,^^10^^,^^15^

In addition to normalizing pArg levels, pegzilarginase statistically significantly decreased plasma levels of GCs, known to be elevated with arginine in plasma and cerebrospinal fluid in ARG1-D.^31^, ^32^, ^33^, ^34^, ^35^, ^36^ In parallel, ornithine (the natural urea cycle product of arginine hydrolysis) was statistically significantly increased, reflecting the enzymatic action of pegzilarginase on pArg. As with pArg levels, decreases in GCs and increases in ornithine were sustained through 48 weeks. It should be noted that since GCs and arginine levels have similar trajectory, the arginine profile is a good predictor of the metabolite profiles. In our study, change from Baseline at Week 24 for GCs geometric mean was calculated using the 168hr timepoint (pre-dose), i.e. at the end of the dosing interval. However, collectively, these results support pegzilarginase as a potentially transformative novel therapy with unprecedented efficacy for ARG1-D and further build the body of evidence implicating arginine as key driver of development and progression of neurologic manifestations, both as proximal toxin and upstream source of neurotoxic GCs.^10^^,^^32^^,^^33^^,^^36^^,^^37^

The normalisation of pArg is considered a relevant surrogate parameter for the entire pool of arginine in patients with ARG1-D. Intracellularly accumulated arginine and its metabolites are transported into the plasma through stimulation of cationic amino acid transport (CAT) systems.^38^, ^39^, ^40^ All CAT proteins are capable of bidirectional transport mediating influx as well as efflux.^39^ The transport of arginine from the liver to plasma, and ultimately to other tissues, in patients with ARG1-D thereby leads to elevated arginine levels in the muscle, kidneys, and cerebrospinal fluid documented in preclinical models as well as patients.^31^^,^^41^, ^42^, ^43^ Reduction of pArg levels results in transport of arginine out of tissues, lowering tissue concentrations, through efflux and back into the plasma, utilizing the same set of transporters. Hence, pArg is representative of tissue arginine levels given this equilibrium flux.^39^^,^^40^^,^^44^

Given the progressive nature of this life-long disorder, it was originally hypothesized that the arginine-lowering effect of pegzilarginase would support clinical stabilization. However, outcomes of the previous study suggested pegzilarginase produced meaningful clinical improvements in functional mobility.^21^^,^^45^ Clinically meaningful improvements, based on the same criteria applied in previous study, were observed in PEACE already after 24 weeks of treatment for all 3 functional assessments.^23^^,^^24^ With long-term treatment, the magnitude of improvements increased and surpassed the thresholds for clinically important response (substantially for some patients). Mean improvements were approximately 2-fold the defined thresholds for GMFM-E and >4-fold the criterion for 2MWT. Analysis of individual patients demonstrated clinically meaningful responses on ≥2 assessments for nearly half of patients on pegzilarginase after 24 weeks.^28^

A number of factors may have contributed to the observed functional mobility outcomes, including the lack of statistical significance in the key secondary endpoints and the differing degree of clinical responses at patient-level. Formal power calculations focused on determining the sample size necessary to show treatment effect on the primary endpoint (pArg). Whereas the pArg response to pegzilarginase was highly consistent across patients, functional mobility outcomes were more variable, likely reflecting differences in disease severity/duration of disease, establishment of functional impairment, and baseline performance. For example, farther distance travelled and GMFM scores near the top of the scale at baseline would influence the observed clinical responses to treatment. This was seen for several patients who demonstrated improvement on 2MWT, GMFM-E, and/or GMFM-D. Many achieved normative distance or neared the maximum possible score, but could not meet thresholds for clinical response because their baseline limited the magnitude of possible effect size. Further, duration of treatment and follow-up in the DB period was relatively short for a life-long, progressive disease. Nonetheless, improvements were evident at 24 weeks and continued treatment during LTE increased the magnitude of improvements. The effect of patient variability may have been compounded by the inclusion of all patients in the treatment arm–level analysis regardless of baseline GMFCS level and the limited sample size. For the placebo/pegzilarginase group it should be noted that pegzilarginase treatment was initiated 6 months later in these patients allowing for further deterioration during the DB period while the change from baseline is calculated in this study. Placebo/pegzilarginase treated patients showed stabilization or numerical improvements. Although statistical significance criteria were not met, improvements were clinically meaningful, meeting and exceeding pre-defined thresholds in a disorder wherein clinical stabilization would typically be viewed as a positive outcome and clinical deterioration is predicted based on historical observations.^46^ The increasing improvement with longer-term treatment suggests potential to halt progression of manifestations, reduce impact of prior disease progression, and improve functional mobility for patients with this debilitating disorder.

A significant burden for patients and their families is the severely restricted diet. Our data proposes potential for liberalization of diet with pegzilarginase treatment, although data is limited given the study procedures to maintain a stable diet. Further real-world data could demonstrate the role of pegzilarginase in diet management of ARG1-D.

Pegzilarginase was well-tolerated and demonstrated a favourable safety profile consistent with the observations in previous studies.^21^^,^^22^ The overall safety profile observed with long-term treatment (up to 125 weeks) was comparable with that previously described for a median of 131 weeks of treatment.^22^ Several reported AEs are known occurrences in ARG1-D (e.g., hyperammonaemia, nausea, vomiting).^1^^,^^15^ HSRs and ISRs were, unlike for other enzyme therapies, infrequent. Hyperammonaemic events were reported at a higher proportion in the placebo arm vs pegzilarginase (36% vs 14%, respectively). Many patients had a documented history of hyperammonaemia, and many of the reported events occurred in the context of potential precipitating factors, i.e., infection. ADAs were uncommon, transient, and without sustained or meaningful impact on arginine reduction or clinical outcomes. More AEs were reported during LTE than DB period, as expected with the longer observation. However, there was no evidence of increasing hypersensitivity, injection site reactions, or immunogenicity with increasing exposure or with transition from IV to SC administration. The favourable and predictable long-term tolerability profile of pegzilarginase is particularly important given the need for life-long therapy and allow for at-home administration to reduce treatment burden on patients, caregivers, and healthcare systems.

This trial represents the largest group of prospectively evaluated patients and the first randomized, blinded, placebo-controlled trial in ARG1-D. With the diverse patient population results are generalizable to the broader population. Pegzilarginase is the first potential disease modifying treatment to normalize pArg levels in ARG1-D, and produced stable, sustained pArg reduction and clinically meaningful functional improvements that continued to increase with longer-term treatment. Together with previous long-term outcomes observed,^21^^,^^22^ our results support pegzilarginase as an effective therapy to normalize arginine, the pathophysiological driver of ARG1-D, and to improve functional mobility outcomes compared with existing management approaches.

## Contributors

Gregory M. Enns contributed to study design, data collection, data interpretation, data verification, and writing and review of the manuscript.

Rossana Sanchez Russo, Serena Gasperini, George A. Diaz contributed to data collection, data interpretation, and writing and review of the manuscript.

Gillian Bubb, Linda Neuman, Leslie S. Sloan contributed to the literature search, study design, data analysis, data interpretation, writing and review of the manuscript, and accessed and verified the data.

All authors approved the final manuscript. All authors had full access to all the data in the study and had final responsibility for the decision to submit for publication.

## Data sharing statement

Data access will be granted in response to qualified collaborative research requests. Aggregate study data can be made available to researchers. Data will be shared on the basis of the scientific merit of the proposal (i.e., the proposal should be scientifically sound, ethical, and have the potential to contribute to the advancement of public health) and the feasibility of the collaborative research proposal (i.e., the requesting research team must be scientifically qualified and have the resources for the proposed project). The data files would exclude data dictionaries that require user licenses. Data could be made available following finalised regulatory authority reviews and at the end of any data exclusivity periods and ending 36 months after the regulatory authority review decision has been received or until the corresponding author is able to fulfil this obligation, whichever is earlier. Furthermore, the study protocol and statistical analysis plan can at this time be made available. Proposals should be directed to the study sponsor. Data requestors will need to sign a data access agreement.

## Declaration of interests

Dr Sanchez Russo has received travel support from Aeglea BioTherapeutics. Dr Gasperini has served on advisory boards for Aeglea BioTherapeutics and Immedica Pharma AB. Dr Neuman and Dr Bubb are former employees of Aeglea BioTherapeutics and owns stocks or equity in the company. Dr Sloan is former employee of Aeglea BioTherapeutics. Dr Diaz has received travel support from and served on advisory boards and speakers bureaus for Aeglea BioTherapeutics. Dr Enns has served on advisory boards for Aeglea BioTherapeutics, and performed consulting services for Horizon Therapeutics.

Aeglea BioTherapeutics funded the trial and provided medical writing support. Immedica Pharma provided scientific editing and medical writing support.

## Appendix A Supplementary data

Supplementary Information

CAEB1102-300A Protocol
