# Supplementary material for: Efficacy and safety of pegzilarginase in arginase 1 deficiency (PEACE): a phase 3, randomized, double-blind, placebo-controlled, multi-centre trial
Source: eClinicalMedicine. 2024 Jan 12;68:102405. doi: 10.1016/j.eclinm.2023.102405 (PMC10825663; doi:10.1016/j.eclinm.2023.102405)

**Supplementary Figure 1. Pre-specified Subgroup Analysis on Severity Demonstrates Treatment Effect on Mobility Function Improvements at Week 24 (Full Analysis Set)**

Group sizes reflect all patients with data; there was no imputation for missing values.

2MWT, 2-minute walk test; GMFCS, Gross Motor Function Classification System; GMFM-E, Gross Motor Function Measure part E


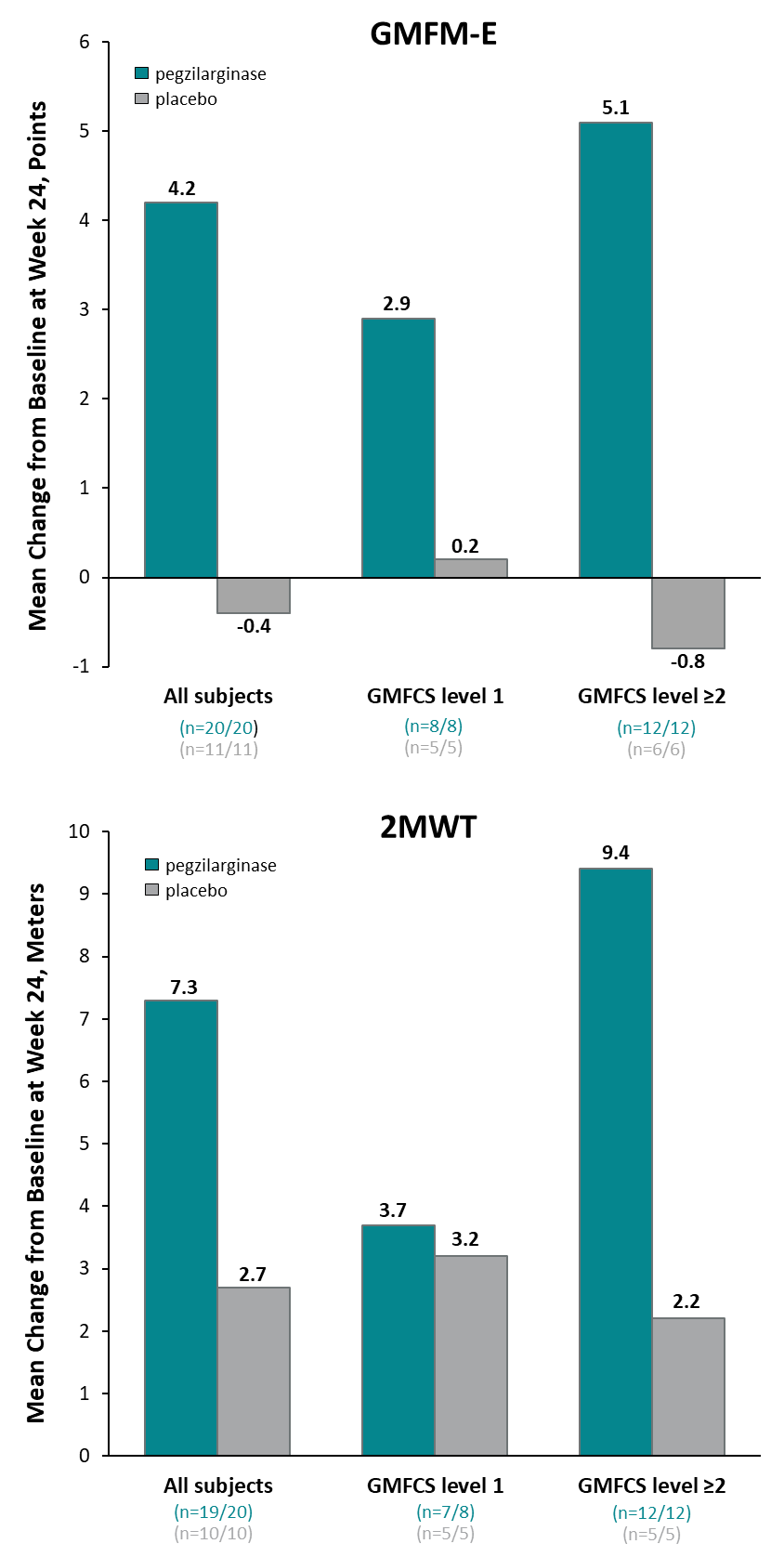

Supplement: Supplementary Figure [file mmc1.docx]
