# Supplementary material for: Efficacy and safety of pegzilarginase in arginase 1 deficiency (PEACE): a phase 3, randomized, double-blind, placebo-controlled, multi-centre trial
Source: eClinicalMedicine. 2024 Jan 12;68:102405. doi: 10.1016/j.eclinm.2023.102405 (PMC10825663; doi:10.1016/j.eclinm.2023.102405)
Supplement: Supplementary Information [file mmc2.pdf]

Efficacy and safety of pegzilarginase in arginase 1 deficiency (PEACE): a Phase 3, Randomized, Double-blind, Placebo-Controlled, Multi-center Trial

Supplementary information

Table of contents

1. Study schema 3

2. Patient recruitment per centre 3

3. Changes to the conduct of the study 4

4. Changes to the planned analyses 6

5. Missing data 7

6. Drug dose, drug concentration, and relationships to response 8

  6.1 Pharmacokinetic analysis 8

  6.2 Pharmacodynamic analysis 8

7. Primary and key secondary analyses by stratification (severity of prior history of hyperammonemia; FAS) 9

1. Study schema

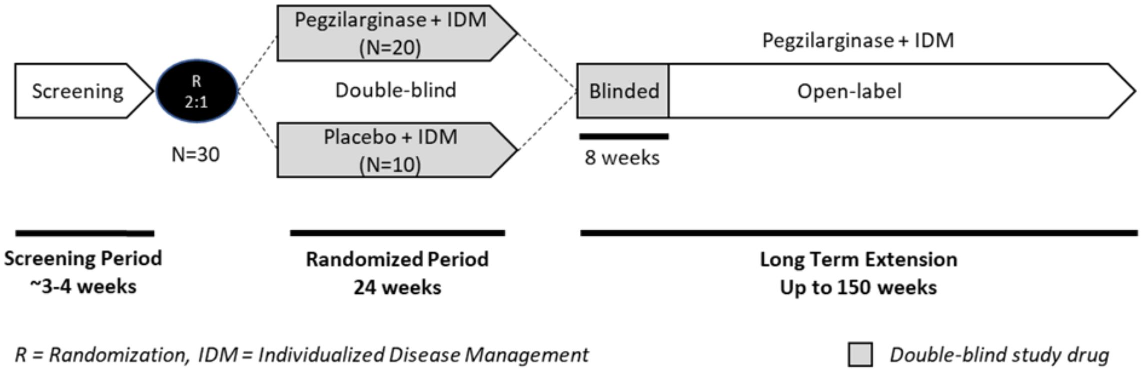

2. Patient recruitment per centre

	Pegzilarginase (n=21)	Placebo (n=11)	Overall (n=32)
**Centre, n (%)**			
Austria 1	2 (9.5)	0 (0.0)	2 (6.3)
Canada 1	1 (4.8)	0 (0.0)	1 (3.1)
France 1	3 (14.3)	1 (9.1)	4 (12.5)
France 2	1 (4.8)	1 (9.1)	2 (6.3)
Germany 1	1 (4.8)	0 (0.0)	1 (3.1)
Italy 1	2 (9.5)	1 (9.1)	3 (9.4)
United Kingdom 1	1 (4.8)	0 (0.0)	1 (3.1)
United Kingdom 2	1 (4.8)	0 (0.0)	1 (3.1)
United Kingdom 3	1 (4.8)	1 (9.1)	2 (6.3)
United Kingdom 4	0 (0.0)	1 (9.1)	1 (3.1)
United States of America 1	1 (4.8)	0 (0.0)	1 (3.1)
United States of America 2	0 (0.0)	1 (9.1)	1 (3.1)
United States of America 3	0 (0.0)	2 (18.2)	2 (6.3)
United States of America 4	1 (4.8)	0 (0.0)	1 (3.1)
United States of America 5	2 (9.5)	1 (9.1)	3 (9.4)
United States of America 6	2 (9.5)	0 (0.0)	2 (6.3)
United States of America 7	0 (0.0)	1 (9.1)	1 (3.1)
United States of America 8	1 (4.8)	0 (0.0)	1 (3.1)
United States of America 9	1 (4.8)	1 (9.1)	2 (6.3)

3. Changes to the conduct of the study

The original protocol was issued on 11 December 2018 and was subsequently amended 6 times. Important changes in each amendment are described below.

Important changes in the conduct of the study

Version Number (Global/Country)	Description of Important Changes
Original Protocol 1.0 (Global)	Not applicable.
2.0 (Global)	• The Schedule of Assessments was updated to clarify timing, add height assessments in the LTE period, specify that mutation analysis and arginase activity in RBCs were required for all subjects, and make other clarifications.<br>• IXRS was clarified that it will evaluate arginine data and send the dose adjustments to the unblinded pharmacist and/or physician; and defining formal unblinding.<br>• Clarification was made to the questions for caregivers.<br>• Hypersensitivity reactions and hyperammonemic episodes were specified as AESIs, and guidance was given regarding corticosteroids.<br>• Additional definition of hyperammonemic episodes was included.
2.1 (UK)	Included all the above changes in addition to clarifying AE reporting from first dose to "from signing of informed consent continuing through the last study follow-up visit."
3.0 (US and Austria)	Note that Version 3.0 included changes made in both Version 2.0 and Version 2.1.<br>• Two additional objectives were added: compare pegzilarginase with placebo re: other aspects of mobility, and compare pegzilarginase with placebo re: VABS-II.<br>• Corresponding endpoints were added for these objectives.<br>• Definitions were clarified; Schedule of Assessments footnotes were clarified.<br>• Definitions of clinical response were added.<br>• Added requirement for a stable, consistent diet through the first 8 weeks of the open-label period.<br>• Clarification was added on measures to minimize bias.<br>• The time period for botulinum toxin use that would exclude a subject from participation was shortened as BT use is standard treatment for spasticity and the decision was made not to have the subjects be without this treatment for a long period.<br>• Storage temperature was corrected, and description of study treatment was updated and clarified.<br>• Initial calculation of dose was clarified to be based on subject's weight at Baseline.<br>• Potential for transition to SC treatment was added.<br>• Limitation of GMFM to Parts D and E were added along with justification for the change.<br>• Subject requirement to maintain dietary protein intake levels consistent with baseline was added.<br>• AESI definitions were added, and terms were defined. Specification of measures to be taken if a hypersensitivity reaction occurs were added.<br>• MCID for Parts D and E were added.<br>• Responder definitions were added.

4.0<br>(Global)<br>Not submitted	Note that Version 4.0 was not submitted as Version 5.0 was developed before Version 4.0 was submitted; thus, Version 4.0 was withdrawn.<br>This version included all changes from Version 3.0.
5.0<br>(Global)	Note that Version 5.0 incorporated changes that were made from Version 2.0 to Version 2.1, Version 2.0 to Version 4.0, Version 3.0 to Version 5.0, and Version 4.0 to Version 5.0. Unique changes in Version 5.0 from Version 3.0 are the following:<br>• Objective was added to compare pegzilarginase with placebo re: objective measures of neurological/neuromotor manifestations; corresponding endpoint was added.<br>• A more comprehensive description of statistical analyses was added.<br>• The option of an interim analysis and the reasoning for an interim analysis were added. Analyses were completed at an overall 2-sided alpha=0.05 without adjustment for multiplicity for the interim analysis.<br>• Blinding/unblinding was clarified regarding personnel and level of unblinding/access.<br>• Timing of assessments for Table 5, Table 6, Table 10, Table 12, Table 13, and Table 15 were revised and adjusted.<br>• SC dosing was added to Section 6.1.<br>• Preparation/Handling/Storage/Accountability section was updated with current data and more specific instructions.<br>• Requirement was added that the first 4 SC doses be given at the investigational site.<br>• SRC having access to full subject data/treatment assignment was added if the SRC has safety concerns.<br>• Sample Size determination was updated with new power calculations.
6.0<br>(Global)	• After review and feedback, objectives, endpoints, and analyses were clarified and reprioritized based on clinical and statistical considerations. The statistical approach was changed to specify the use of continuous rather than categorical variables, and multiple comparison procedures were specified for global control of Type 1 error.<br>• Additional changes were made to ensure continuity and safety of study subjects despite the impact of the global pandemic.<br>• The key secondary objective was revised to be based on key mobility and/or motor function outcome measures, and the accompanying endpoint was revised accordingly.<br>• Clarifications were made to the secondary objectives and endpoints and the tertiary objectives and endpoints.<br>• For the primary endpoint analysis was specified that the values would be log transformed prior to analysis.<br>• Sensitivity analyses were added.<br>• Contingency for action if the primary analysis is or is not statistically significant were added.<br>• Clarifications were made to the Schedule of Assessments footnotes.<br>• A section was added addressing the impact of COVID-19.<br>• Preparation/Handling/Storage/Accountability was revised to reflect new data.<br>• Clarification was made re: when the IXRS would be discontinued.<br>• Specification for reporting additional follow-up SAE information was added.<br>• Injection site reactions were added to the AESIs and a definition of Injection site reactions was added.<br>• Revisions were made to the section on hyperammonemia for accuracy and clarity.<br>• Sample size determination was adjusted to reflect inclusion of Study 102A preliminary data and specification of the number of units corresponding to the log scale.<br>• Sample size was adjusted to align with the changes in statistical analyses/endpoints/objectives.<br>• Additional detail was included for the statistical analyses consistent with the change in the statistical approach.

Abbreviations: AE=adverse event; AESI=adverse event of special interest; BT=botulinum toxin; COVID-19=coronavirus disease of 2019; IXRS=interactive web/voice response system; GMFM=Gross Motor

Function Measure; LTE=long-term extension; MCID=minimum clinically important difference; RBC=red blood cells; SAE=serious adverse event; SC=subcutaneous; SRC=Safety Review Committee; UK=United Kingdom; US=United States; VABS-II=Vineland Adaptive Behavior Scale, Second edition.

4. Changes to the planned analyses

The original SAP was issued on 11 December 2020 and was revised twice during the study. Important changes in each amendment are described below.

Important changes to the planned analyses

Version Number	Description of Important Changes
Original SAP 1.0	Not applicable.
2.0	The primary purpose of SAP Version 2.0 was to better define analyses of the study endpoints and to update the methods based upon interactions with regulatory authorities, as well as to better define the analysis periods and timepoints.<br><br>Updates are summarized below.<br><br>• **Description of change:** Power calculation was updated to reflect the updated primary endpoint of change from baseline in logged plasma arginine.<br><br>**Rationale for change:** Following correspondence with authority it was agreed to define primary endpoint and key secondary endpoints using change from baseline.<br><br>• **Description of change:** Analysis periods and timepoints were added, and corresponding text was revised as appropriate.<br><br>**Rationale for change:** To clarify.<br><br>• **Description of change:** Subject disposition analyses were clarified. History of hyperammonemic episodes was added to demographic variables, and baseline disease characteristics were added and defined. Prior and concomitant medications were clarified. Dosing compliance and treatment exposure were clarified.<br><br>**Rationale for change:** To clarify.<br><br>• **Description of change:** A new section regarding protocol deviations was added.<br><br>**Rationale for change:** To define protocol deviations and corresponding outputs.<br><br>• **Description of change:** Text was revised to more accurately reflect the endpoints being measured and their corresponding derivations, analyses, and outputs, as applicable. Sub-sections were added, revised, or moved accordingly.<br><br>**Rationale for change:** To clarify and to address feedback from regulatory authorities.<br><br>• **Description of change:** Changes to protocol-stated analyses were updated, and changes to the SAP were added.<br><br>**Rationale for change:** To accurately depict the development of the analysis plan.<br><br>• **Incorporation of administrative changes.** Other minor, administrative changes have been incorporated throughout the SAP and are noted in the redline version.

3.0	The primary purpose for SAP Version 3.0 was to update some analyses depending on data availability and the need to provide particular summaries, as well as to update the analysis periods defined in the outputs.<br><br>Updates are summarized below.<br><br>• **Description of change:** Some planned tables were removed. Listings will still be generated.<br><br>**Rationale for change:** Following a blinded dry run of the data it was determined that some of the planned outputs do not have enough subjects for the purpose of summarizing in a table, or that the tables were deemed unnecessary and that listings will suffice.<br><br>• **Description of change:** The definitions of analysis periods were updated.<br><br>**Rationale for change:** To better align with the intent of the study and to better clarify the descriptions of the DB and LTE periods, both in the text and in the outputs.<br><br>• **Description of change:** Composite Clinical Outcome: 2MWT, GMFM Part D, and GMFM Part E: The frequencies of subjects achieving response was updated to reflect ≥1, ≥2, or 3 of the categories (from 1, 2, 3). Additionally, and additional definition of responder was included (defined as having an improvement in at least 1 component assessment regardless of worsening in any other component).<br><br>**Rationale for change:** To remove ambiguity in the definition and to provide additional information.<br><br>• **Description of change:** A summary of Adverse event during the LTE period were added, as well as a summary of exposure-adjusted incidence rates (EAIRs).<br><br>**Rationale for change:** To better evaluate and compare AEs and AE rates between the DB and LTE periods.<br><br>• **Description of change:** The section defining Liver Function Test abnormalities was updated and moved.<br><br>**Rationale for change:** For clarity as it was not considered an AESI.

5. Missing data

Primary Endpoint Arginine: Among the 32 enrolled subjects, 1 subject in the pegzilarginase arm dropped out early (Week 6). As a result, the subject doesn't have arginine data at Week 24 (primary endpoint). This data was the only missing data for the primary endpoint. Hence, since the amount of missing data is negligible, the investigation of missing data pattern associated with the MMRM model was not conducted. In the actual execution, the one missing data point was inputted using the last observation carried forward (LOCF) method as per the SAP.

Secondary Endpoint 2MWT: For the 2-minute walk test endpoint, 3 subjects (2 in the pegzilarginase arm, and 1 in the placebo arm) had missing data. In the pegzilarginase group 1 subject [2 years old, female] had no BL 2MWT distance as they were considered too young to be able to perform the assessment; the second subject had no week 24 2MWT assessment as they withdrew from the study at Week 6. The placebo subject with missing 2MWT data at Week 24 was unable to complete the Week 24 assessment as they had developed an adverse event and could not walk. The subject

developed severe right knee pain at Week 11 and was subsequently diagnosed with Osgood Schlatter disease and Sinding Larsen and Johansson disease.

Secondary Endpoint GMFM-E: For the GMFM-E endpoint, 1 subject (the Week 6 dropout subject; pegzilarginase arm) had missing data.

Since none of the key secondary endpoints reached statistical significance, there was no merit in pursuing the investigation of missing data pattern and conducting the SAP specified sensitivity analysis.

6. Drug dose, drug concentration, and relationships to response

6.1 Pharmacokinetic analysis

- PK concentration data following QW IV administration are available for all 21 subjects who were enrolled into Study 300A and received treatment with pegzilarginase. Nineteen of these subjects had reportable PK parameters at Week 1, Week 12, and Week 24.
- Adequate PK exposure was obtained with IV administration within the dose range of 0.05 to 0.2 mg/kg, which was consistent with the dose-response relationship observed in previous Studies 101A and 102A.
- The median time to maximum observed pegzilarginase concentration (t_{max}) generally occurred rapidly (≤4.7 hours after the start of infusion), as expected following a nominal 0.5 hours QW IV infusion of pegzilarginase.
- The pegzilarginase PK exposures (C_{max} and AUC_{0-168}) after IV administration increased in an approximately dose-proportional manner across the dose range of 0.05 to 0.2 mg/kg at steady state after repeat QW dosing (Week 12 and Week 24).
- The mean $t_{1/2}$ was approximately 40 hours (range: 37.3 to 43 hours), which was similar across doses and whether a single dose or at steady state.
- Steady state was achieved on or before Week 12 based on the available data and sampling time; however, it is theoretically expected to be reached after 2 weeks of consistent QW dosing based on $t_{1/2}$.
- Exposures were relatively constant for Week 12 and Week 24 versus Week 1, with negligible accumulation after weekly dosing.

6.2 Pharmacodynamic analysis

- The rapid onset of action $t_{1/2}$ of pegzilarginase resulted in early, consistent, and sustained reductions in plasma arginine.
- At all timepoints evaluated post dosing (Week 1, Week 12, and Week 24), arginine remained below the upper limit of normal for the majority of the QW dosing interval. At Weeks 12 and 24, plasma arginine levels at post-dose were generally lower than baseline values for all subjects and all doses, and below the guideline recommended level (<200 µM) for all but 1 subject.

- Individualized dose adjustments to reduce arginine to medical guidance (<200 µM) precluded a definitive demonstration of dose response following repeated dosing.
- After LTE09, subjects could switch to QW SC administration of pegzilarginase. Doses from IV to SC were to remain the same unless the dosing algorithm dictated otherwise. Subcutaneous dosing demonstrated continued maintenance of 168-hour post-dose arginine levels to below the recommended guideline of <200 µM for most of the plasma arginine levels, similar to those achieved after IV administration (consistent with what was observed in Study 102A).

7. Primary and key secondary analyses by stratification (severity of prior history of hyperammonemia; FAS)

	History of hyperammonemia		No history of hyperammonemia	
**Visit**	**Pegzilarginase**	**Placebo**	**Pegzilarginase**	**Placebo**
**pArg (µmol/L), Mean (CV)**				
Baseline	340.3 (0.30)	448.8 (0.23)	373.1 (0.21)	484.6 (0.14)
Week 24	71.4 (0.25)	472.8 (0.13)	111.4 (0.64)	376.9 (0.36)
**GMFM-E (points), Mean (SD)**				
Baseline	50.7 (21.8)	41.3 (30.7)	45.2 (17.9)	52.6 (15.7)
Week 24	52.8 (23.6)	43.3 (30.9)	50.6 (18.7)	49.4 (20.8)
**2MWT (meters), Mean (SD)**				
Baseline	114.0 (57.7)	94.2 (63.2)	101.5 (55.7)	106.8 (30.0)
Week 24	122.1 (58.1)	87.3 (62.8)	106.5 (42.5)	124.8 (11.9)

Abbreviations: 2MWT: 2-minute walk test; CV: coefficient of variation; FAS: full analysis set; GMFM-E: gross motor function measure part E; pArg: plasma arginine; SD: standard deviation
