# Supplementary material for: Efficacy and safety of pegzilarginase in arginase 1 deficiency (PEACE): a phase 3, randomized, double-blind, placebo-controlled, multi-centre trial
Source: eClinicalMedicine. 2024 Jan 12;68:102405. doi: 10.1016/j.eclinm.2023.102405 (PMC10825663; doi:10.1016/j.eclinm.2023.102405)
Supplement: CAEB1102-300A Protocol [file mmc3.pdf]

TITLE PAGE

Protocol Title: PEACE (Pegzilarginase Effect on Arginase 1 Deficiency Clinical Endpoints): A Randomized, Double-Blind, Placebo-Controlled Phase 3 Study of the Efficacy and Safety of Pegzilarginase in Children and Adults with Arginase 1 Deficiency

Protocol Number:	CAEB1102-300A
Compound Name/Number:	Pegzilarginase/AEB1102
Study Phase:	Phase 3
Acronym:	PEACE
Sponsor Name:	Aeglea BioTherapeutics, Inc.
Legal Registered Address:	805 Las Cimas Pkwy, Suite 100 Building III<br>Austin, TX 78746
Regulatory Agency Identifier Number(s):	
IND Number:	127774
EudraCT Number:	2018-004837-34
Protocol Date:	Version 6.0 11 December 2020<br>Version 5.0: 08 June 2020<br>Version 4.0: 02 March 2020 (not submitted)<br>Version 3.0: 25 July 2019 (submitted in US and Austria only)<br>(replaces both Version 2.0: 15 January 2019<br>and Version 2.1: May 17, 2019,<br>UK country-specific amendment)<br>Version 1.0: December 11, 2018

TABLE OF CONTENTS

TITLE PAGE 1

TABLE OF CONTENTS 2

SIGNATURE PAGE 9

INVESTIGATOR'S AGREEMENT 10

1. SYNOPSIS 11

1.1. Schema 18

1.2. Schedules of Assessments 19

2. INTRODUCTION 63

2.1. Study Rationale 63

2.2. Background 64

2.3. Benefit-Risk Assessment 65

3. OBJECTIVES AND ENDPOINTS 67

4. STUDY DESIGN 69

4.1. Overall Design 69

4.2. Impact of Coronavirus Disease (COVID-19) 69

4.3. Scientific Rationale for Study Design 70

4.3.1. Use of Placebo 70

4.3.2. Study Population 71

4.4. Measures to Minimize Bias: Randomization and Blinding 72

4.4.1. Description of Blinding Levels and Access 72

4.4.2. Breaking the Blind 73

4.5. Justification for Dose 76

4.6. Definitions of End of Randomized Period and End of Study 76

4.6.1. End of Randomized Period 76

4.6.2. End of Study 76

5. STUDY POPULATION 77

5.1. Inclusion Criteria 77

5.2. Exclusion Criteria 78

5.3. Screen Failures and Re-Screening 79

6. STUDY MEDICATION 80

6.1. Study Medications Administered 80

6.2. Preparation/Handling/Storage/Accountability 81
6.3. Study Medication Compliance 82
6.4. Concomitant Therapy 82
6.5. Dose Modification 83
6.6. Treatment Options 83
7. DISCONTINUATION 85
7.1. Discontinuation of Study Medication 85
7.2. Lost to Follow Up 85
8. STUDY ASSESSMENTS AND PROCEDURES 86
8.1. Informed Consent/Assent 86
8.2. Demographics and Medical History 86
8.3. Physical Examination (Including Neurological Examination) 88
8.4. Neuromotor Assessments 89
8.4.1. 2-Minute Walk Test 89
8.4.2. Functional Mobility Assessment 90
8.4.3. 9-Hole Pegboard Test 90
8.4.4. Gross Motor Function Measures 90
8.4.5. Modified Ashworth Scale 91
8.5. Adaptive Behavior 91
8.6. Assessments of Caregiver and Clinician Global Impressions 92
8.6.1. Caregiver and Clinician Global Impressions of Change 92
8.6.1.1. Questions Regarding Change to Be Independently Administered to Caregivers and Clinicians 92
8.6.1.2. Possible Responses 93
8.6.2. Caregiver and Clinician Global Impressions of Severity 93
8.6.2.1. Questions Regarding Severity to Be Independently Administered to Caregivers and Clinicians 93
8.6.2.2. Possible Responses 94
8.6.3. Use of Caregiver and Clinician Global Impressions as Anchors 94
8.7. Seizure Frequency 94
8.8. Neurocognitive and Quality-of-Life Assessments 95
8.8.1. Neurocognitive Assessments 96
8.8.1.1. Wechsler Adult Intelligence Scale IV (WAIS-IV) 96

8.8.1.2. Wechsler Intelligence Scale for Children V (WISC-V) 96
8.8.1.3. Wechsler Preschool and Primary Scale of Intelligence IV (WPPSI-IV)....96
8.8.1.4. Bayley Scales of Infant and Toddler Development III (BSID-III)....97
8.8.2. Quality-of-Life Assessments 97
8.8.2.1. Measurement Model for the Pediatric Quality of Life Inventory (PedsQL) 97
8.8.2.2. 36-Item Short Form Health Survey (SF-36) 97
8.8.2.3. Short Form Burden Interview (ZBI-12) 98
8.9. Assessment of Growth....98
8.10. Exit Interview 98
8.11. Clinical Laboratory Tests 98
8.12. DNA Sample....100
8.13. Home Health Care 100
8.14. Disease Management Plan and 3-Day Diary Diet Record....101
8.15. Arginine, Ornithine, and Guanidino Compounds....101
8.16. Plasma Amino Acids and Ammonia....102
8.17. Vital Signs and Height and Weight 102
8.18. Electrocardiogram....102
8.19. Electroencephalogram 103
8.20. Pharmacokinetics....103
8.21. Anti-Drug Antibody Testing....104
8.22. Follow-Up....104
9. SAFETY ASSESSMENTS 105
9.1. Adverse Events, Serious Adverse Events, and Laboratory Test Abnormalities 105
9.1.1. Serious Adverse Events 107
9.2. Treatment of Overdose 108
9.3. Adverse Events of Special Interest 108
9.3.1. Hypersensitivity Reactions 108
9.3.2. Injection Site Reactions 109
9.3.3. Hyperammonemic Episodes 109
9.3.4. Reporting an Adverse Event of Special Interest....110
9.4. Laboratory Test Abnormality 110
9.5. Independent Safety Review Committee 110

10. STATISTICAL CONSIDERATIONS 112
10.1. Sample Size Determination 112
10.2. Populations for Analyses/Analysis Sets 112
10.3. Statistical Analyses 113
10.3.1. Efficacy Analyses 113
10.3.1.1. Details for Analysis of the Primary Endpoint 115
10.3.1.2. Details for Analysis of the Key Secondary Mobility Endpoints 116
10.3.1.3. Details for Analysis of the Secondary Efficacy Endpoints 116
10.3.2. Safety Analyses 118
10.3.3. Other Analyses 118
10.3.4. Stratification of Randomization 118
10.4. Interim Analyses 118
11. SUPPORTING DOCUMENTATION AND OPERATIONAL CONSIDERATIONS 119
11.1. Regulatory and Ethical Considerations 119
11.2. Privacy and Confidentiality 119
11.3. Data Quality Assurance 120
11.4. Management of Deviations 120
11.5. Electronic Case Report Forms and Source Documents 120
11.5.1. Study and Site Closure 120
11.5.2. Use of Information and Publication Policy 121
11.5.3. Retention of Records 121
11.5.4. Study Monitoring, Auditing, and Inspections 121
12. ABBREVIATIONS 123
13. REFERENCES 126

LIST OF TABLES

Table 1: Schedule of Assessments – Screening Period 19

Table 2: Schedule of Assessments – Randomized Double-Blind Period (Baseline through Study Day 85)......21

Table 3: Schedule of Assessments – Randomized Double-Blind Period (Days 92 to 162/EORP)......24

Table 4: Schedule of Assessments – LTE01-LTE13 27

Table 5: Schedule of Assessments – Open-Label LTE14-LTE26......30

Table 6: Schedule of Assessments – Open-Label LTE27-LTE39......33

Table 7: Schedule of Assessments – Open-Label LTE40-LTE52......36

Table 8: Schedule of Assessments – Open-Label LTE53-LTE65......39

Table 9: Schedule of Assessments – Open-Label LTE66-LTE78......42

Table 10: Schedule of Assessments – Open-Label LTE79-LTE91......45

Table 11: Schedule of Assessments – Open-Label LTE92-LTE104......48

Table 12: Schedule of Assessments – Open-Label LTE105-LTE117......51

Table 13: Schedule of Assessments – Open-Label LTE118-LTE130......54

Table 14: Schedule of Assessments – Open-Label LTE131-LTE143......57

Table 15: Schedule of Assessments – Open-Label LTE144-LTE150/EOS 60

Table 16: Objectives and Endpoints 67

Table 17: Definition of Baseline Deficits for Key Secondary/Other Secondary Endpoints 71

Table 18: Study Personnel and Levels of Data Access and/or Unblinding 74

Table 19: Information to Be Collected for Subjects Who Are Screen Failures......79

Table 20: Study Medications Administered 80

Table 21: Dose Adjustments for Pegzilarginase 83

Table 22: Elements of Complete Medical and Surgical History 87

Table 23: Neuromotor Function Clinical Outcomes Assessments 89

Table 24: Adaptive Behavior 91

Table 25: Neurocognitive and Quality of Life (QoL) Assessments 95

Table 26: Clinical Laboratory Assessments 99

Table 27: Pharmacokinetic Sampling Times 103

Table 28: Adverse Event Relationship to Study Medication......106

Table 29: Analysis Sets......112

Table 30: Detailed Overview of Statistical Methodology for Efficacy Endpoints 114
Table 31: Definition of Baseline Deficits for Other Secondary Clinical Endpoints* 115
Table 32: Additional Clinical Response Definitions 117
Table 33: Brief Overview of Statistical Methodology for Safety Endpoints 118

LIST OF FIGURES

Figure 1: Study Schema for CAEB1102-300A 18

SIGNATURE PAGE

Sponsor's Approval

The protocol has been electronically approved by Aeglea BioTherapeutics, Inc. December 2020.

Responsible Medical Officer:
Eric Bradford, MD, MSc
Chief Development Officer

Sponsor's Medical Monitor:

Rick A Martin, MD
Aeglea BioTherapeutics, Inc.
805 Las Cimas Pkwy, Suite 100 Building III
Austin, TX 78746
United States
Tel: 512-942-2935

INVESTIGATOR'S AGREEMENT

Protocol Title: PEACE (Pegzilarginase Effect on Arginase 1 Deficiency Clinical Endpoints): A Randomized, Double-Blind, Placebo-Controlled Phase 3 Study of the Efficacy and Safety of Pegzilarginase in Children and Adults with Arginase 1 Deficiency

Protocol Number: CAEB1102-300A Version 6.0

I have read the Study CAEB1102-300A Protocol Version 6.0 and agree to conduct the study as outlined. I agree to maintain the confidentiality of all information received or developed in connection with this protocol.

____

Printed Name of Investigator

____

Signature of Investigator

____

Date

1. SYNOPSIS

Protocol Title:

PEACE (Pegzilarginase Effect on Arginase 1 Deficiency Clinical Endpoints): A Randomized, Double-Blind, Placebo-Controlled Phase 3 Study of the Efficacy and Safety of Pegzilarginase in Children and Adults with Arginase 1 Deficiency

Rationale:

Subjects with arginase 1 deficiency (ARG1-D) show persistent hyperargininemia and continued disease progression despite current individualized disease management (IDM) consisting of severe protein restriction and essential amino acid (EAA) supplementation and/or the use of ammonia scavengers. The failure to adequately lower plasma arginine levels with IDM into the normal range (40 to 115 µM), or even below the current guideline-recommended level of 200 µM in most cases is believed to be due to both the practical challenges of adhering to a protein-restricted diet rigorous enough to lower plasma arginine levels and the important contribution of whole-body protein turnover to plasma arginine flux.

Two lines of evidence support the concept that lowering plasma arginine levels has the potential to slow disease progression in subjects with ARG1-D. First, in subjects exposed to sustained high levels of arginine due to a delayed disease diagnosis, the subsequent lowering of plasma arginine with severe dietary protein restriction has been demonstrated to lead to some modest improvements in disease manifestations. Second, in a severely affected pediatric case unresponsive to IDM approaches, lowering of plasma arginine using an exchange blood transfusion to enhance extra-hepatic arginase activity resulted in an improvement in clinical status, including a reduction in spasticity.

Pegzilarginase has been shown in previous studies to produce marked, rapid, and sustained reductions in plasma arginine levels in subjects with ARG1-D, allowing substantially improved arginine control relative to what can be achieved with IDM approaches. The improved control of plasma arginine levels was accompanied by clinical improvements in 1 or more instruments of neuromotor function or adaptive behavior, consistent with the hypothesis that improved plasma arginine control has the potential to slow disease progression in affected subjects. The goal of the current study is to confirm these preliminary findings in a blinded, placebo-controlled design and to gain further understanding of the impact of pegzilarginase on a broader range of disease manifestations.

Objectives and Endpoints:

Objectives	Endpoints
PRIMARY	
• To demonstrate the efficacy of pegzilarginase relative to placebo based on a statistically significant decrease in plasma arginine concentrations	• Change from Baseline in plasma arginine after 24 weeks of study treatment
KEY SECONDARY	
• To demonstrate the efficacy of pegzilarginase relative to placebo based on key mobility outcome measures	• Mean change from Baseline at Week 24 in the 2-Minute Walk Test (2MWT)<br>• Mean change from Baseline at Week 24 in the Gross Motor Function Measure-88 (GMFM) Part E
SECONDARY	
To further demonstrate the clinical and biochemical efficacy of pegzilarginase relative to placebo	
• To compare pegzilarginase with placebo with respect to the proportion of subjects whose endpoint arginine value falls below target guidance of 200 µM	• A subject is considered a responder if their endpoint arginine value is <200 µM after 24 weeks of study treatment, and a non-responder otherwise
• To compare pegzilarginase with placebo with respect to the proportions of subjects whose endpoint arginine value falls within the normal range of 40 to 115 µM	• A subject is considered a responder if their endpoint arginine value is in the normal range (≥40 µM and ≤115 µM) at Week 24, and a non-responder otherwise
• To compare pegzilarginase with placebo for changes in ornithine and guanidino compounds (GCs)	• Change from Baseline in ornithine and GCs after 24 weeks of study treatment
• To compare pegzilarginase with placebo with respect to other aspects of mobility	• Mean change from Baseline at Week 24 in the GMFM Part D<br>• Mean change from Baseline at Week 24 in Functional Mobility Scale (FMS) and Gillette Functional Assessment Questionnaire (GFAQ)
• To compare pegzilarginase with placebo with respect to adaptive behavior	• Mean change from Baseline at Week 24 in Vineland Adaptive Behavior Scales (VABS)-II
• To evaluate the safety and immunogenicity of pegzilarginase	• Adverse events (AEs) and anti-drug antibodies (ADAs) will be collected
• To further characterize the pharmacokinetic (PK) profile of pegzilarginase	• PK and PD data will be analyzed

Objectives	Endpoints
TERTIARY	
• To compare the proportions of subjects with response in composite clinical outcome, on pegzilarginase and placebo	• Clinical response is defined as a subject exhibiting a mobility response in the 2MWT or GMFM Part D, or GMFM Part E as defined in the body of the protocol
• To compare response on pegzilarginase with placebo with respect to other aspects of mobility	• Response for FMS and GFAQ is defined as a 1-level change at any distance and a 2-level change at any distance, respectively
• To compare response on pegzilarginase with placebo with respect to adaptive behavior	• Response for VABS-II is defined as an improvement by ≥7.5 points
• To compare pegzilarginase with placebo with respect to improvement of spasticity and objective measures of neurological/neuromotor manifestations	• Change from Baseline for functional and spasticity measurements by Modified Ashworth Scale (MAS)
• To further describe caregiver and clinician global impression of the impact of pegzilarginase on motor function and/or adaptive behavior	• Caregiver/Clinician Global Impression of Change, Global Impression of Severity
• To describe the impact of pegzilarginase on fine motor function	• 9-Hole Pegboard
• To describe the impact of pegzilarginase on quality of life (QoL)	• Pediatric Quality of Life (PedsQL),<br>• 36-Item Short Form Health Survey (SF-36)<br>• Zarit Burden Interview (12-item) (ZBI-12)
• To describe the impact of pegzilarginase on neurocognition and memory	• Neurocognitive endpoints:<br>– Bayley Scales of Infant Development (BSID)-III (as age appropriate)<br>– Wechsler Intelligence Batteries
• To describe the diets maintained by subjects in the study	• 3-day diet records will be collected
• To describe the effect of pegzilarginase on plasma ammonia	• Plasma ammonia data will be collected
• To evaluate the effects of pegzilarginase on growth in pediatric subjects	• Z-scores for height, weight, and body mass index (BMI) will be computed using the Centers for Disease Control and Prevention (CDC) growth curves for subjects younger than 18 years of age
• To characterize the long-term (>24 to approximately 178 weeks) treatment effects of pegzilarginase administration	• Endpoints from long-term extension period data will be used, in particular to evaluate the extent to which efficacy is sustained over the period from Week 24 to Week 48

Overall Design:

CAEB1102-300A is a multi-center, randomized, double-blind, placebo-controlled study to evaluate the safety and efficacy of pegzilarginase in subjects with ARG1-D. This study will consist of:

1. A screening period of 3 to 4 weeks duration to collect all necessary information to ensure the subjects meet study eligibility criteria and to establish Baseline plasma arginine data, collect prescribed diet data, and determine adherence to prescribed diet using a diet diary.
2. A randomized, double-blind treatment period of 24 weeks.
3. An open-label long-term extension (LTE) period of up to approximately 150 weeks in which all subjects receive active pegzilarginase. The first 8 weeks of treatment will remain blinded to ensure that study data relating to the randomized period is collected prior to unblinding.

Subjects will be randomized to treatment following completion of all screening assessments and confirmation of study eligibility in a 2:1 ratio to receive weekly intravenous (IV) infusions of pegzilarginase plus IDM or placebo plus IDM during the 24-week double-blind treatment period. After completion of the 24-week double-blind treatment period, each subject will enter the LTE period, subjects will have the option to receive pegzilarginase by SC, with Investigator and Sponsor approval. The first 4 SC doses will be given at the investigational site. The initial mg/kg SC dose may be the same as the IV dose. Subsequent doses may be administered outside of the investigational site by appropriately trained home healthcare personnel if considered safe and appropriate in the opinion of the Investigator in consultation with the Sponsor. During the LTE period, all subjects will receive pegzilarginase plus IDM.

Number of Subjects:

A sufficient number of subjects will be screened so that at least 30 subjects in the Full Analysis Set have at least 1 follow-up measurement on which the key secondary endpoint can be assessed (2MWT and GMFM-E).

Subjects who discontinue after their first dose but before completing the double-blind portion (“dropouts”) will not be replaced; however, all subjects, including dropouts, are included in the analyses of efficacy and safety as specified in the body of the protocol.

Intervention Groups and Duration:

The total duration of the study is expected to be approximately 178 weeks per subject, including the LTE period. Subjects will receive weekly IV infusions (approximately 30 minutes) of pegzilarginase or volume-adjusted placebo once weekly. Dose modifications of pegzilarginase based solely on plasma arginine values will be implemented by the unblinded pharmacist and/or physician, according to an algorithm. Following completion of the assessments in this study, subjects may continue to receive pegzilarginase through other means.

Subjects assigned to pegzilarginase begin at Dose Level 2, 0.10 mg/kg. Beginning with Visit 5, dose modifications, if required, based on plasma arginine values will be implemented by the unblinded pharmacist and/or physician, according to the following algorithm that will be implemented in the interactive voice response system (IXRS) up to LTE24:

- If the plasma arginine level is >150 µM, a single 168-hour sample will be used to increase the dose by 2 dose levels (not to exceed 0.20 mg/kg) if the 2 doses prior to this sample were a) the same dose level in mg/kg, and b) consecutive (with no missed doses).
- If the plasma arginine levels from *2* sequential 168-hour samples (regardless of missed doses) are both <50 µM, the dose is decreased by 1 dose level, not to decrease below 0.05 mg/kg.

Full details are provided in the Pharmacy Manual.

Infusion or dosing of study medication will be interrupted in any subject developing signs or symptoms of a hypersensitivity reaction. Hypersensitivity reactions will be treated according to standard of care at the clinical site. The infusion or dosing may be resumed if, in the opinion of the Investigator, and after consultation with the Sponsor, the symptoms can be safely managed. Hypersensitivity reactions observed in Study CAEB1102-101A (Study 101A) were typically managed with antihistamines and a slower infusion rate. The benefits and risks of corticosteroid treatment will be carefully considered given their potential to cause hyperammonemia in this patient population.

A subject may withdraw from the study at any time at his/her own request or may be withdrawn at any time at the discretion of the Investigator or Sponsor at any time for any reason, without prejudice to further treatment.

Study Population:

Participating subjects, aged ≥2 years, must provide written informed consent/assent, have a diagnosis of ARG1-D as documented in medical records, which must include 1 of the following: elevated plasma arginine levels, a mutation analysis that results in a pathogenic variant, or reduced red blood cell (RBC) arginase activity. Subjects must also be assessable for clinically meaningful within-subject change on specific protocol endpoints, be currently on and willing to maintain a stable diet with respect to protein consumption, be currently on and willing to maintain stable doses of certain medications that address the symptoms of ARG1-D, and agree to take measures to avoid pregnancy.

Subjects may not participate in the study if they have had a hyperammonemic episode within 6 weeks of the first dose, have known active infections, have extreme mobility deficit, have any medical conditions that would interfere with study compliance or data interpretation, have participated in a previous pegzilarginase study or have hypersensitivity to polyethylene glycol (PEG), are currently receiving or plan to receive therapy containing botulinum toxin for spasticity-related complications during the double-blind or blinded follow-up portions of the study or received surgical or botulinum-toxin treatment for spasticity-related complications within the 16 weeks prior to the first dose of study treatment in this study, or have had previous liver or hematopoietic transplant.

Statistical Analyses:

The statistical analysis plan (SAP) will be developed and finalized before any unblinding of the double-blinded period and will describe the subject populations to be included in the analyses, and procedures for accounting for missing, unused, and spurious data. This section is a summary of the planned statistical analyses of the primary and key secondary endpoints.

For efficacy analyses of pegzilarginase versus placebo, subjects will be categorized according to the treatment to which they were randomized.

For safety analyses, subjects will be categorized according to the treatment that they actually received. Subjects receiving a single dose of pegzilarginase will be categorized in the pegzilarginase treatment arm for all safety outputs.

The analysis set that will be used for both efficacy and safety analysis is the Full Analysis Set, (FAS), defined as all subjects who consented, were randomized, and received at least 1 dose of Investigational Medicinal Product (IMP).

For the primary endpoint, change from Baseline in plasma arginine, the plasma arginine values will be log transformed prior to analysis.

The Baseline value will be defined as the mean of all logged plasma arginine values (analyzed at the designated central laboratory) obtained during the screening/Baseline period and prior to the first dose of blinded study treatment. If a subject was re-screened, only those values taken during the final screening period are used in the computation of Baseline arginine.

The final follow-up arginine level will be defined as the mean of the last 4 prior-to-dosing logged values obtained during the double-blind period that meet the following criteria: (1) the sample date occurred after the scheduled date for the 20th dose, and (2) the prior 2 doses were administered as planned, ie, at approximately 7 days and 14 days prior to collection of the arginine sample. If at least 1, but fewer than 4, values meet the criteria, then only those values will be included in the mean. If none of the values meet the criteria, then the last single post-Baseline arginine value will be included.

If no post-Baseline arginine values were obtained, then the change from Baseline will be imputed as 0. Otherwise, change from Baseline will be equal to (Final follow-up arginine level logged value – Baseline plasma arginine logged value).

Change from Baseline to final follow-up arginine level as defined in this section will be compared between randomized groups using an ANCOVA model using 2-sided α=0.05. The baseline value will be included as a covariate in the model. Details of any other covariates and class effects to be included in the ANCOVA will be detailed in the SAP.

Once exponentiated, the change from baseline in logged values will represent a ratio. The difference in change from baseline values will represent a relative ratio in plasma arginine for subjects treated with pegzilarginase vs subjects treated with placebo.

As a sensitivity analysis, change from Baseline to final follow-up arginine level as defined in this section will be compared between pegzilarginase and placebo with a Wilcoxon rank sum test.

To assess the impact of dropouts, a second sensitivity analysis using a Wilcoxon rank sum test will be performed where those subjects who drop out for reasons deemed related to IMP will receive lower rank than those who complete all 24 weeks or who drop out for reasons clearly not related to IMP. Details will be provided in the SAP.

Supportive subgroup analyses may be performed if there are ≥4 subjects in each subgroup. The process for determining subgroups of interest will be defined in the SAP.

If the primary analysis is not statistically significant, formal hypothesis testing will stop and p-values for key secondary endpoints will be descriptive only. If the primary analysis is statistically significant, then formal hypothesis testing will proceed with the key secondary mobility endpoints.

The global Type 1 error level with respect to the testing of the 2 key secondary endpoints will be controlled using the Hochberg adjustment for multiplicity.

Change from Baseline at Week 24 in 2MWT and GMFM-E will be compared between randomized groups using separate ANCOVA models. The respective baseline values will be included as a covariate in each model. Details of any other covariates and class effects the models to be included in the ANCOVA models will be detailed in the SAP.

Technical details of the statistical methodology for the primary and key secondary endpoints are provided in the body of the protocol.

Interim Analyses

No interim analyses are planned during the blinded portion of the study. When all subjects have completed the blinded portion of the study to LTE01 and its assessments, and the database has been frozen, the data will be formally unblinded and analyzed.

Safety Review Committee:

Primary oversight for subject safety will be the responsibility of the Investigators and Sponsor. Additional advisory oversight of subject safety will be provided by an independent Safety Review Committee (SRC) composed of individuals with pertinent medical expertise, who will serve in advisory capacity to the Sponsor to provide an additional level of oversight to minimize the chance that clinical study subjects are exposed to unreasonable or unnecessary risks. To enable the assessment of the benefit:risk of pegzilarginase during the study, members of the independent SRC will have access to unblinded individual subject-level safety data as needed. If the SRC has any safety concerns, they may obtain full access to subjects' treatment assignment.

1.1. Schema

Figure 1: Study Schema for CAEB1102-300A

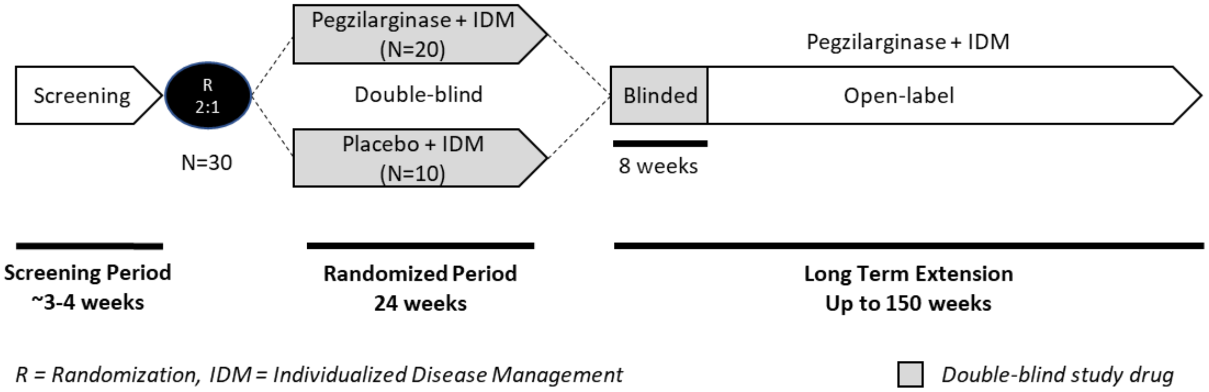

1.2. Schedules of Assessments

Table 1: Schedule of Assessments – Screening Period

<table>
<tr><th rowspan="2">Visit ID</th><th colspan="3">Screening</th></tr>
<tr><th>Scr1</th><th>Scr2</th><th>Scr3</th></tr>
<tr><td>Study Day (±1)</td><td>-28 to -21</td><td>-14</td><td>-7</td></tr>
<tr><td>Informed Consent and Subject History</td><td></td><td></td><td></td></tr>
<tr><td>Informed consent (or informed consent for rescreening if required by IRB/EC)</td><td>X</td><td></td><td></td></tr>
<tr><td>Inclusion / Exclusion Criteria</td><td>X</td><td></td><td></td></tr>
<tr><td>Demographics</td><td>X</td><td></td><td></td></tr>
<tr><td>Disease characteristics[a]</td><td>X</td><td></td><td></td></tr>
<tr><td>General medical history, prior medications</td><td>X</td><td></td><td></td></tr>
<tr><td>Mutation analysis of ARG1 gene[b]</td><td colspan="3">X</td></tr>
<tr><td>Arginase Activity in RBCs[c]</td><td colspan="3">X</td></tr>
<tr><td>Clinical Assessments</td><td></td><td></td><td></td></tr>
<tr><td>Height, head circumference</td><td>X</td><td></td><td></td></tr>
<tr><td>Weight</td><td>X</td><td></td><td></td></tr>
<tr><td>Vital signs (prior-to-dosing on dosing days)[d]</td><td>X</td><td></td><td>X</td></tr>
<tr><td>EEG[e]</td><td></td><td colspan="2">X</td></tr>
<tr><td>ECG</td><td>X</td><td></td><td></td></tr>
<tr><td>Physical examination[f]</td><td>X</td><td></td><td></td></tr>
<tr><td>Neurological examination[g]</td><td>X</td><td></td><td></td></tr>
<tr><td>Laboratory</td><td></td><td></td><td></td></tr>
<tr><td>Pregnancy Test[h]</td><td>X</td><td></td><td></td></tr>
<tr><td>Arginine and ornithine</td><td>X</td><td>X</td><td>X</td></tr>
<tr><td>Guanidino compounds</td><td>X</td><td colspan="2">X</td></tr>
<tr><td>Plasma amino acids and ammonia[i]</td><td>X</td><td></td><td></td></tr>
<tr><td>Hematology, coagulation[j]</td><td>X</td><td></td><td></td></tr>
<tr><td>Blood chemistryj</td><td>X</td><td></td><td></td></tr>
<tr><td>Urinalysisj</td><td>X</td><td></td><td></td></tr>
<tr><td>Anti-drug antibodies (ADAs)</td><td></td><td>X</td><td></td></tr>
<tr><td>Outcomes</td><td></td><td></td><td></td></tr>
<tr><td>Individualized Diet Management questionnaire[k]</td><td>X</td><td>X</td><td>X</td></tr>
<tr><td>Adaptive behavior, QoL</td><td colspan="3">X</td></tr>
<tr><td>Neuromotor function assessments[l]</td><td colspan="3">X</td></tr>
<tr><td>Neurocognitive development[m]</td><td colspan="3">X</td></tr>
<tr><td>Safety</td><td></td><td></td><td></td></tr>
<tr><td>Adverse Events</td><td>X</td><td>X</td><td>X</td></tr>
<tr><td>Concomitant Medications</td><td>X</td><td>X</td><td>X</td></tr>
</table>

EC = Ethics Committee; EEG = electroencephalogram; IRB = Institutional Review Board; ECG = electrocardiogram; QoL = quality of life; RBC = red blood cell; Scr = Screening.
NOTE: Study visits will be conducted approximately every 7 days during the study. A window of 1 day has been specified but visits that occur outside of this window due to subject or site schedule or other reasons will not be considered a protocol deviation.

[a] Includes historical values of arginine, as available, for the past 1 year, history of protein restriction, amino acid supplementation, and use of ammonia scavengers. Also includes history of newborn screening, cognitive and language delays, motor function delays, diet, hyperammonemic episodes (Section 9.3.2), family and sibling history of ARG1-D, hepatic disorders, and non-hyperammonemic hospitalizations.
[b] This assessment may be performed at any of the 3 Screening visits. Required for all subjects in accordance with local or country laws or regulations. If subject's medical record contains the results of a previous mutation analysis, that is sufficient. Some countries may require a separate, optional consent/assent; if a subject does not consent/assent, they may still be enrolled in the study.
[c] This assessment may be performed at any of the 3 Screening visits. Required for all subjects.
[d] Vital signs include systolic and diastolic blood pressure, pulse, temperature, and respirations prior to dosing and as clinically indicated.
[e] Normal, outpatient EEG
[f] Full physical examination (PE) at Screening. Subsequent PEs will be brief examinations to document clinically significant changes from Baseline.
[g] Neurological examination is described in Section 8.3.
[h] Serum pregnancy test will be collected at screening and end of treatment; all other timepoints can be urine or serum tests (females of childbearing potential only).
[i] All amino acid and ammonia samples will be taken after a 4-hour fast when clinically and logistically feasible. The time of the last meal and administration of EAAs must be recorded.
[j] See Section 8.11.
[k] Individualized diet management questionnaire is a record of protein prescribed and consumed. During the screening period, the protein consumption information will be distributed at Day -21 and collected on the 3 days prior to the -14 day, -7 day, and Baseline visits.
[l] Neuromotor assessments are detailed in Section 8.4. Post-Baseline assessments will be accompanied by "anchor" questions to assess subject, caregiver, and/or clinician impression of clinical changes in specific domains. At visits where neuromotor assessments are conducted, the 2MWT will be the first neuromotor assessment performed. If neuromotor tests cannot be scheduled during the 28-day screening period, the Baseline visit may be delayed by up to 2 weeks to accommodate these assessments.
[m] Required neurocognitive assessments by age are detailed in Section 8.8. If neurocognitive tests cannot be scheduled during the 28-day screening period, the Baseline visit may be delayed by up to 2 weeks to accommodate these assessments.

Table 2: Schedule of Assessments – Randomized Double-Blind Period (Baseline through Study Day 85)

**Special Visit Designation**	**BL**												
**Visit ID**	**1**	**2**	**3**	**4**	**5**	**6**	**7**	**8**	**9**	**10**	**11**	**12**	**13**
**Study Day**	**1**	**8**	**15**	**22**	**29**	**36**	**43**	**50**	**57**	**64**	**71**	**78**	**85**
Inclusion/Exclusion Criteria	X												
Drug Administration													
Randomization	X												
Double-blind IMP administration	X	X	X	X	X	X	X	X	X	X	X	X	X
Clinical Assessments													
Height, head circumference													X
Weight	X			X				X				X	
Vital signs (prior to dosing on dosing days)[a]	X	X	X	X	X	X	X	X	X	X	X	X	X
EEG[b]													
12-Lead ECG	X												X
Physical examination[c]	X												X
Neurological examination[d]	X												X
Laboratory													
Pregnancy Test[e]	X					X						X	
Prior to dosing arginine and ornithine	X	X	X	X	X	X	X	X	X	X	X	X	X
Arginine post-dose profile[f]	X											X	
Guanidino compounds	X	X		X				X				X	
Plasma amino acids and ammonia[g]	X					X				X			
Hematology, coagulation[h]	X					X				X			
Blood chemistry[h]	X			X		X				X			
Urinalysis[h]	X					X						X	
Growth hormone and IGF-1	X												
Tryptase, complement C3[i]	X												

**Special Visit Designation**	**BL**												
**Visit ID**	**1**	**2**	**3**	**4**	**5**	**6**	**7**	**8**	**9**	**10**	**11**	**12**	**13**
**Study Day**	**1**	**8**	**15**	**22**	**29**	**36**	**43**	**50**	**57**	**64**	**71**	**78**	**85**
ADAs[j]	X	X					X					X	
Phenylbutyrate Metabolite Analysis[k]	X												
Hypersensitivity reaction: additional tryptase, complement C3, and ADA samples[l]													
Hyperammonemic episode: additional phenylbutyrate metabolite samples[m]													
PK Samples[n]	X											X	
Outcomes													
Individualized Diet Management questionnaire[o]	X					X						X	
Seizure frequency	X	X	X	X	X	X	X	X	X	X	X	X	X
Adaptive behavior, QoL												X	
Neuromotor function assessments[p]												X	
Neurocognitive development[q]													
C/C Global Impressions of Severity[r]	X											X	
C/C Global Impressions of Change												X	
Exit interviews[s]													
Safety													
Adverse events	X	X	X	X	X	X	X	X	X	X	X	X	X
Concomitant medications	X	X	X	X	X	X	X	X	X	X	X	X	X
4 weeks after last dose telephone follow up (for subjects who discontinue from study)[t]													

ADAs = anti-drug antibodies; BL = Baseline; C/C = Caregiver and Clinician EEG = electroencephalogram; ECG = electrocardiogram; EORP = End of Randomized Period; FU = Follow Up; GC = guanidino compound; IGF-1 = insulin-like growth factor-1; IMP = investigational medicinal product; PE = physical examination; PK = pharmacokinetic(s); QoL = quality of life; RBC = red blood cell; Scr = Screening.
All assessments on a dosing day are to be obtained prior to dose administration unless otherwise indicated.
NOTE: Study visits will be conducted every 7 days ±1 day during the study.

[a] Vital signs include systolic and diastolic blood pressure, pulse, temperature, and respirations prior to dosing and as clinically indicated.

[b] Normal, outpatient EEG
[c] Brief physical examination to document clinically significant changes from Baseline.
[d] Neurological examination is described in Section 8.3.
[e] Serum pregnancy test will be collected at screening and end of treatment; all other time points can be urine or serum tests (females of childbearing potential only)
[f] Arginine post-dose profiles will be collected when feasible at approximately 1 hour (±30 minutes), 2 hours (±30 minutes), 4 hours (±1 hour), 24 hours (±4 hours), and 96 hours (±8 hours) after completion of the infusion. The 24-hour and 96-hour samples may be collected using Home Health Care services where appropriate. Efforts will be made to collect the samples within the specified windows where feasible, but samples collected outside the windows will not be considered protocol deviations, but the time and date must be recorded.
[g] Samples for determination of amino acids and ammonia will be collected prior to dosing. All amino acid and ammonia samples will be drawn after a 4-hour fast when clinically and logistically feasible. The time of the last dose of EAAs must be recorded.
[h] See Section 8.11.
[i] Samples for tryptase and complement C3 will be collected at Baseline. Samples will be shipped to the central laboratory.
[j] Samples for ADAs will be collected at Baseline and pre-dose at regular intervals throughout the study according to the Schedules of Assessments. Samples will be shipped to the central laboratory
[k] Samples for phenylbutyrate metabolite analysis will be collected at Baseline. Samples will be shipped to the central laboratory.
[l] In the event of a hypersensitivity reaction, additional tryptase, complement C3, and ADA samples will be taken at 3 and 24 hours after the start of the reaction. collected. Samples will be shipped to the central laboratory.
[m] In the event of a hyperammonemic episode, additional phenylbutyrate metabolite samples will be taken at the same time as 1 of the ammonia samples (preferably the first) is collected. Samples will be shipped to the central laboratory. Any additional blood samples collected to monitor the subject during a hyperammonemic episode will be shipped to the central laboratory for analysis, if possible.
[n] PK samples will be collected where feasible within approximately 1 hour prior to initiation of the infusion and at approximately 1 hour (±30 minutes), 2 hours (±30 minutes), 4 hours (±1 hour), 24 hours (±4 hours), 96 hours (±8 hours), and 168 hours (±24 hours, but prior to next dose) after completion of the infusion. The 24-hour and 96-hour samples may be collected using Home Health Care services where appropriate. Efforts will be made to collect the samples within the specified windows where feasible, but samples collected outside the windows will not be considered protocol deviations, but the time and date must be recorded.
[o] Individualized diet management questionnaire is a record of protein prescribed and consumed. During the double-blind period, protein prescribed and consumed will be collected on the 3 days prior to Visits 6, 12, 18, and 24.
[p] Neuromotor assessments are detailed in Section 8.4. Post-Baseline assessments will be accompanied by “anchor” questions to assess subject, caregiver, and/or clinician impression of clinical changes in specific domains. At visits where neuromotor assessments are conducted, the 2MWT will be the first neuromotor assessment performed. Every effort will be made to schedule neuromotor assessments on their assigned weeks; if this is not possible, these assessments may be delayed by up to 2 weeks.
[q] Required neurocognitive assessments by age are detailed in Section 8.8. Post-Baseline assessments will be accompanied by “anchor” questions to assess subject, caregiver, and/or clinician impression of clinical changes in specific domains. Every effort will be made to schedule neurocognitive assessments on their assigned weeks; if this is not possible, these assessments may be delayed by up to 2 weeks.
[r] This assessment may be performed any time before initiation of treatment.
[s] Exit is from double-blind portion of study.
[t] The final dose telephone follow-up will occur 4 weeks after the subject receives the final dose of pegzilarginase, regardless of when that final dose is administered.

Table 3: Schedule of Assessments – Randomized Double-Blind Period (Days 92 to 162/EORP)

**Special Visit Designation**											**EORP**
**Visit ID**	**14**	**15**	**16**	**17**	**18**	**19**	**20**	**21**	**22**	**23**	**24**
**Study Day**	**92**	**99**	**106**	**113**	**120**	**127**	**134**	**141**	**148**	**155**	**162**
Drug Administration											
Double-Blind IMP Administration	X	X	X	X	X	X	X	X	X	X	X
Clinical Assessments											
Height, head circumference											X
Weight			X				X				X
Vital signs (prior to dosing on dosing days)[a]	X	X	X	X	X	X	X	X	X	X	X
EEG[b]											X
ECG											X
Physical examination[c]											X
Neurological examination[d]											X
Laboratory											
Pregnancy Test[e]					X						X
Prior-to-dosing arginine and ornithine	X	X	X	X	X	X	X	X	X	X	X
Arginine post-dose profile[f]											X
Guanidino compounds			X				X				X
Plasma amino acids and ammonia[g]		X					X				X
Hematology, coagulation[h]		X					X				X
Blood chemistry[h]		X					X				X
Urinalysis[h]							X				X
Tryptase, complement C3[i]											
ADAs[j]				X							X
Hypersensitivity reaction: additional tryptase, complement C3, and ADA samples[k]											

Special Visit Designation											EORP
**Visit ID**	**14**	**15**	**16**	**17**	**18**	**19**	**20**	**21**	**22**	**23**	**24**
**Study Day**	**92**	**99**	**106**	**113**	**120**	**127**	**134**	**141**	**148**	**155**	**162**
Hyperammonemic episode: additional phenylbutyrate metabolite samples [l]											
PK Samples[m]											X
Outcomes											
Individualized Diet Management questionnaire[n]					X						X
Seizure frequency	X	X	X	X	X	X	X	X	X	X	X
Adaptive behavior, QoL											X
Neuromotor function assessments[o]											X
Neurocognitive development[p]											X
C/C Global Impressions of Severity											X
C/C Global Impressions of Change											X
Exit interview[q]											X
Safety											
Adverse events	X	X	X	X	X	X	X	X	X	X	X
Concomitant medications	X	X	X	X	X	X	X	X	X	X	X
4 weeks after last dose telephone follow-up (for subjects who discontinue from study)[r]											X

ADAs = anti-drug antibodies; C/C = Caregiver and Clinician; EEG = electroencephalogram; ECG = electrocardiogram; EORP = End of Randomized Period Visit; FU = Follow-Up Visit; GC = guanidino compound; IGF-1 = insulin-like growth factor-1; IMP = investigational medicinal product; PE = physical examination; PK = pharmacokinetic(s); QoL = quality of life; RBC = red blood cell; Scr = Screening.
All assessments on a dosing day are to be obtained prior to dose administration unless otherwise indicated.
NOTE: Study visits will be conducted every 7 days +/- 1 day during the study.

[a] Vital signs include systolic and diastolic blood pressure, pulse, temperature, and respirations prior to dosing and as clinically indicated.
[b] Normal, outpatient EEG.
[c] Brief physical examination to document clinically significant changes from Baseline.
[d] Neurological examination is described in Section 8.3.
[e] Serum pregnancy test will be collected at screening and end of treatment; all other timepoints can be urine or serum tests (females of childbearing potential only).

[f] Arginine post-dose profiles will be collected when feasible at approximately 1 hour (±30 minutes), 2 hours (±30 minutes), 4 hours (±1 hour), 24 hours (±4 hours), and 96 hours (±8 hours) after completion of the infusion. The 24-hour and 96-hour samples may be collected using Home Health Care services where appropriate. Efforts will be made to collect the samples within the specified windows where feasible, but samples collected outside the windows will not be considered protocol deviations, but the time and date must be recorded.

[g] Samples for determination of amino acids and ammonia will be collected prior-to-dosing. All amino acid and ammonia samples will be drawn after a 4-hour fast when clinically and logistically feasible. The time of the last dose of EAAs must be recorded.

[h] See Section 8.11.

[i] Samples for tryptase and complement C3 will be collected at Baseline. Samples will be shipped to the central laboratory.

[j] Samples for ADAs will be collected at Baseline and pre-dose at regular intervals throughout the study per the Schedules of Assessments. Samples will be shipped to the central laboratory.

[k] In the event of a hypersensitivity reaction, additional tryptase, complement C3, and ADA samples will be taken at 3 and 24 hours after the start of the reaction. Samples will be shipped to the central laboratory.

[l] In the event of a hyperammonemic episode, additional phenylbutyrate metabolite samples will be taken at the same time as the ammonia samples are collected. Samples will be shipped to the central laboratory.

[m] PK samples will be collected where feasible within approximately 1 hour prior to initiation of the infusion and at approximately 1 hour (± 30 minutes), 2 hours (± 30 minutes), 4 hours (± 1 hour), 24 hours (±4 hours), 96 hours (±8 hours), and 168 hours (±24 hours, but prior to next dose) after completion of the infusion. The 24-hour and 96-hour samples may be collected using Home Health Care services where appropriate. Efforts will be made to collect the samples within the specified windows where feasible, but samples collected outside the windows will not be considered protocol deviations, but the time and date must be recorded.

[n] Individualized diet management questionnaire is a record of protein prescribed and consumed. During the double-blind period, protein prescribed and consumed will be collected on the 3 days prior to Visits 6, 12, 18, and 24.

[o] Neuromotor assessments are detailed in Section 8.4. Baseline assessments must be performed within 14 days prior to first dose. Post-Baseline assessments will be accompanied by "anchor" questions to assess subject, caregiver, and/or clinician impression of clinical changes in specific domains. At visits where neuromotor assessments are conducted, the 2MWT will be the first neuromotor assessment performed. Every effort will be made to schedule neuromotor assessments on their assigned weeks; if this is not possible, these assessments may be delayed by up to 2 weeks.

[p] Required neurocognitive assessments by age are detailed in Section 8.8. Baseline assessments must be performed within 14 days prior to the first dose. Post-Baseline assessments will be accompanied by "anchor" questions to assess subject, caregiver, and/or clinician impression of clinical changes in specific domains. Every effort will be made to schedule neurocognitive assessments on their assigned weeks; if this is not possible, these assessments may be delayed by up to 2 weeks.

[q] Exit is from the randomized, double-blind portion of the study.

[r] The final dose telephone follow-up will occur 4 weeks after the subject receives the final dose of pegzilarginase, regardless of when that final dose is administered.

Table 4: Schedule of Assessments – LTE01-LTE13

**Blinding**	**Double-Blind (8 weeks)**									**Open-Label (142 weeks)**			
**Visit ID**	**LTE01**	**LTE02**	**LTE03**	**LTE04**	**LTE05**	**LTE06**	**LTE07**	**LTE08**	**LTE09**[a]	**LTE10**	**LTE11**	**LTE12**	**LTE13**
**LTE Study Day**	**1**	**8**	**15**	**22**	**29**	**36**	**43**	**50**	**57**	**64**	**71**	**78**	**85**
Drug Administration													
Double-blind IMP administration	X	X	X	X	X	X	X	X	X				
Open-label IMP administration										X	X	X	X
Clinical Assessments													
Height and weight				X					X				X
Vital signs (prior to dosing on dosing days)[b]	X	X	X	X	X	X	X	X	X	X	X	X	X
ECG									X				
Physical examination[c]									X				
Neurological examination[d]									X				
Laboratory													
Pregnancy Test[e]				X					X				
Prior to dosing arginine and ornithine	X	X	X	X	X	X	X	X	X	X	X	X	X
Guanidino compounds	X				X		X		X				X
Plasma amino acids and ammonia[f]									X				
Hematology, coagulation[g]									X				
Blood chemistry[g]									X				
Urinalysis[g]									X				
Growth hormone and IGF-1									X				
Tryptase, complement C3[h]													
ADAs[i]	X	X					X					X	

Blinding	Double-Blind (8 weeks)									Open-Label (142 weeks)			
**Visit ID**	**LTE01**	**LTE02**	**LTE03**	**LTE04**	**LTE05**	**LTE06**	**LTE07**	**LTE08**	**LTE09[a]**	**LTE10**	**LTE11**	**LTE12**	**LTE13**
**LTE Study Day**	**1**	**8**	**15**	**22**	**29**	**36**	**43**	**50**	**57**	**64**	**71**	**78**	**85**
Phenylbutyrate Metabolite Analysis[j]	X												
Hypersensitivity reaction – additional tryptase, complement C3, ADA samples[k]													
Hyperammonemic episode – additional phenylbutyrate metabolite samples[l]													
Outcomes													
Individualized Diet Management questionnaire[m]					X				X				
Seizure frequency	X	X	X	X	X	X	X	X	X	X	X	X	X
Adaptive behavior, QoL												X	
Neuromotor function assessments[n]												X	
Neurocognitive development[o]													
Safety													
Adverse events	X	X	X	X	X	X	X	X	X	X	X	X	X
Concomitant medications	X	X	X	X	X	X	X	X	X	X	X	X	X
4 weeks after final dose telephone follow-up[p]													

ADAs = anti-drug antibodies; ECG = electrocardiogram; EOS = End-of-Study Visit; FU = Follow-Up Visit; GC = guanidino compound; IGF-1 = Insulin-like Growth Factor-1; IMP = investigational medicinal product; LTE = long-term extension; PE = physical examination; PK = pharmacokinetic(s); QoL = quality of life.

Subjects may continue treatment in the open-label portion of the study until the study drug becomes available through another means or the Investigator, Sponsor or subject determine it is no longer in the subject's interest to receive the study medication. If any subject is participating in the Blinded Follow-up when the data from the Randomized Period are locked and unblinded, the remaining visits for that subject in the Blinded Follow-up can be unblinded.

All assessments on a dosing day are to be obtained prior to dose administration unless otherwise indicated.

NOTE: Study visits will be conducted every 7 days ±1 day during the study.

[a] After Week 8 (LTE08) of the LTE period and with agreement of the Investigator and Sponsor, subjects may switch to SC administration. It is recommended that if subjects are switched to SC dosing, at least the first 4 SC doses be administered in the clinic. After the 4th SC dose in the clinic, if appropriate in the opinion of the Investigator, home health care visits may be utilized. Subjects will be required to return to the site thereafter as clinically indicated in the opinion of the Investigator dependent on the assessments that need to be conducted.

[b] Vital signs include systolic and diastolic blood pressure, pulse, temperature, and respirations prior to dosing and as clinically indicated.

[c] Brief physical examination to document clinically significant changes from Baseline.

[d] Neurological examination is described in Section 8.3.

[e] Serum pregnancy test will be collected at screening and end of treatment; all other timepoints can be urine or serum tests (females of childbearing potential only).

[f] Samples for determination of amino acids and ammonia will be collected prior to dosing. All amino acid and ammonia samples will be drawn after a 4-hour fast when clinically and logistically feasible. The time of the last dose of EAAs must be recorded.

[g] See Section 8.11

[h] Samples for tryptase and complement C3 will be collected at LTE01. Samples will be shipped to the central laboratory.

[i] Samples for ADAs will be collected at LTE01 and pre-dose at regular intervals throughout the study according to the Schedules of Assessments. Samples will be shipped to the central laboratory.

[j] Samples for phenylbutyrate metabolite analysis will be collected at LTE01 and a sample should be taken upon the occurrence of any hyperammonemic episode at the same time as 1 of the ammonia samples is collected. Phenylbutyrate metabolite analysis samples will be shipped to the central laboratory.

[k] In the event of a hypersensitivity reaction additional tryptase, complement C3, and ADA samples will be taken at 3 and 24 hours after the start of the reaction. Samples will be shipped to the central laboratory.

[l] In the event of a hyperammonemic episode, additional phenylbutyrate metabolite samples will be taken at the same time as ammonia samples are collected. Samples will be shipped to the central laboratory.

[m] Individualized Diet Management questionnaire is a record of protein prescribed and consumed. During the LTE period, protein prescribed and consumed will be collected on the 3 days prior to Visits LTE05, LTE09, and then every 12 weeks.

[n] Neuromotor assessments are detailed in Section 8.4. At visits where neuromotor assessments are conducted, the 2MWT will be the first neuromotor assessment performed. Every effort will be made to schedule neuromotor assessments on their assigned weeks; if this is not possible, these assessments may be delayed by up to 2 weeks.

[o] Required neurocognitive assessments by age are detailed in Section 8.8. Post-Baseline assessments will be accompanied by “anchor” questions to assess subject, caregiver, and/or clinician impression of clinical changes in specific domains. Required neurocognitive assessments are to be conducted approximately every 6 months during the follow-up period beginning from the first blinded dose in this period. Every effort will be made to schedule neurocognitive assessments on their assigned weeks; if this is not possible, these assessments may be delayed by up to 2 weeks.

[p] The final dose telephone follow-up will occur 4 weeks after the subject receives the final dose of pegzilarginase, regardless of when that final dose is administered.

Table 5: Schedule of Assessments – Open-Label LTE14-LTE26

**Blinding**	**Open-Label (142 weeks)**												
**Visit ID**	**LTE14**	**LTE15**	**LTE16**	**LTE17**	**LTE18**	**LTE19**	**LTE20**	**LTE21**	**LTE22**	**LTE23**	**LTE24**	**LTE25**	**LTE26**
**LTE Study Day**	**92**	**99**	**106**	**113**	**120**	**127**	**134**	**141**	**148**	**155**	**162**	**169**	**176**
Drug Administration													
Open-label IMP administration	X	X	X	X	X	X	X	X	X	X	X	X	X
Clinical Assessments													
Height and weight				X				X				X	
Vital signs (prior to dosing on dosing days)[a]	X	X	X	X	X	X	X	X	X	X	X	X	X
ECG								X					
Physical examination[b]								X					
Neurological examination[c]								X					
Laboratory													
Pregnancy Test[d]				X				X				X	
Prior to dosing arginine and ornithine[e]	X	X	X	X	X	X	X	X	X	X	X[f]		
Guanidino compounds				X				X			X		
Plasma amino acids and ammonia[g]								X					
Hematology, coagulation[h]								X					
Blood chemistry[h]								X					
Urinalysis[h]								X					
Growth hormone and IGF-1								X					
Tryptase, complement C3[i]													
ADAs[j]				X							X		
Phenylbutyrate Metabolite Analysis[k]													

Blinding	Open-Label (142 weeks)												
**Visit ID**	**LTE14**	**LTE15**	**LTE16**	**LTE17**	**LTE18**	**LTE19**	**LTE20**	**LTE21**	**LTE22**	**LTE23**	**LTE24**	**LTE25**	**LTE26**
**LTE Study Day**	**92**	**99**	**106**	**113**	**120**	**127**	**134**	**141**	**148**	**155**	**162**	**169**	**176**
Hypersensitivity reaction – additional tryptase, complement C3, ADA samples[l]													
Hyperammonemic episode – additional phenylbutyrate metabolite samples[m]													
Outcomes													
Individualized Diet Management questionnaire[n]								X					
Seizure frequency	X	X	X	X	X	X	X	X	X	X	X	X	X
Adaptive behavior, QoL											X		
Neuromotor function assessments[o]											X		
Neurocognitive development[p]											X		
Safety													
Adverse events	X	X	X	X	X	X	X	X	X	X	X	X	X
Concomitant medications	X	X	X	X	X	X	X	X	X	X	X	X	X
4 weeks after final dose telephone follow-up[q]													

ADAs = anti-drug antibodies; ECG = electrocardiogram; EOS = End-of-Study Visit; FU = Follow-Up Visit; GC = guanidino compound; IGF-1 = Insulin-like Growth Factor-1; IMP = investigational medicinal product; LTE = long-term extension; PE = physical examination; PK = pharmacokinetic(s); QoL = quality of life.
Subjects may continue treatment in the open-label portion of the study until the study drug becomes available through another means or the Investigator, Sponsor or subject determine it is no longer in the subject's interest to receive the study medication. If any subject is participating in the Blinded Follow-up when the data from the Randomized Period are locked and unblinded, the remaining visits for that subject in the Blinded Follow-up can be unblinded.
All assessments on a dosing day are to be obtained prior to dose administration unless otherwise indicated.
NOTE: Study visits will be conducted every 7 days ±1 day during the study.

[a] Vital signs include systolic and diastolic blood pressure, pulse, temperature, and respirations prior to dosing and as clinically indicated.
[b] Brief physical examination to document clinically significant changes from Baseline.
[c] Neurological examination is described in Section 8.3.

[d] Serum pregnancy test will be collected at screening and end of treatment; all other timepoints can be urine or serum tests (females of childbearing potential only).
[e] After LTE24, prior to dosing arginine and ornithine samples are required every 4 weeks but may be performed more frequently as clinically indicated at the discretion of the Investigator.
[f] Pre-dose samples are required every 4 weeks but may be performed more frequently at the discretion of the Investigator if clinically indicated.
[g] Samples for determination of amino acids and ammonia will be collected prior to dosing. All amino acid and ammonia samples will be drawn after a 4-hour fast when clinically and logistically feasible. The time of the last dose of EAAs must be recorded.
[h] See Section 8.11.
[i] Samples for tryptase and complement C3 will be collected at LTE01. Samples will be shipped to the central laboratory.
[j] Samples for ADAs will be collected at LTE01 and pre-dose at regular intervals throughout the study according to the Schedules of Assessments. Samples will be shipped to the central laboratory.
[k] Samples for phenylbutyrate metabolite analysis will be collected at LTE01 and upon the occurrence of any hyperammonemic episode at the same time as ammonia samples are collected. Phenylbutyrate metabolite analysis samples will be shipped to the central laboratory.
[l] In the event of a hypersensitivity reaction, additional tryptase, complement C3, and ADA samples will be taken at 3 and 24 hours after the start of the reaction. Samples will be shipped to the central laboratory.
[m] In the event of a hyperammonemic episode, additional phenylbutyrate metabolite samples will be taken at the same time as ammonia samples are collected. Samples will be shipped to the central laboratory.
[n] Individualized Diet Management questionnaire is a record of protein prescribed and consumed. During the LTE period, protein prescribed and consumed will be collected on the 3 days prior to Visits LTE18 and LTE24 and then every 12 weeks.
[o] Neuromotor assessments are detailed in Section 8.4. At visits where neuromotor assessments are conducted, the 2MWT will be the first neuromotor assessment performed. Every effort will be made to schedule neuromotor assessments on their assigned weeks; if this is not possible, these assessments may be delayed by up to 2 weeks.
[p] Required neurocognitive assessments by age are detailed in Section 8.8. Required neurocognitive assessments are to be conducted approximately every 6 months during the follow-up period beginning from the first blinded dose in this period. Every effort will be made to schedule neurocognitive assessments on their assigned weeks; if this is not possible, these assessments may be delayed by up to 2 weeks.
[q] The final dose telephone follow-up will occur 4 weeks after the subject receives the final dose of pegzilarginase, regardless of when that final dose is administered.

Table 6: Schedule of Assessments – Open-Label LTE27-LTE39

**Blinding**	**Open-Label (142 weeks)**												
**Visit ID**	**LTE27**	**LTE28**	**LTE29**	**LTE30**	**LTE31**	**LTE32**	**LTE33**	**LTE34**	**LTE35**	**LTE36**	**LTE37**	**LTE38**	**LTE39**
**LTE Study Day**	**183**	**190**	**197**	**204**	**211**	**218**	**225**	**232**	**239**	**246**	**253**	**260**	**267**
Drug Administration													
Open-label IMP administration	X	X	X	X	X	X	X	X	X	X	X	X	X
Clinical Assessments													
Height and weight			X				X				X		
Vital signs (prior to dosing on dosing days)[a]	X	X	X	X	X	X	X	X	X	X	X	X	X
ECG							X						
Physical examination[b]							X						
Neurological examination[c]							X						
Laboratory													
Pregnancy Test[d]			X				X				X		
Prior to dosing arginine and ornithine[e]		X				X				X			
Guanidino compounds		X				X				X			
Plasma amino acids and ammonia[f]							X						
Hematology, coagulation[g]							X						
Blood chemistry[g]							X						
Urinalysis[g]							X						
Growth hormone and IGF-1							X						
Tryptase, complement C3[h]													

**Blinding**	**Open-Label (142 weeks)**												
**Visit ID**	**LTE27**	**LTE28**	**LTE29**	**LTE30**	**LTE31**	**LTE32**	**LTE33**	**LTE34**	**LTE35**	**LTE36**	**LTE37**	**LTE38**	**LTE39**
**LTE Study Day**	**183**	**190**	**197**	**204**	**211**	**218**	**225**	**232**	**239**	**246**	**253**	**260**	**267**
ADAs[i]							X						
Phenylbutyrate Metabolite Analysis[j]													
Hypersensitivity reaction – additional tryptase, complement C3, ADA samples[k]													
Hyperammonemic episode – additional phenylbutyrate metabolite samples[l]													
Outcomes													
Individualized Diet Management questionnaire[m]							X						
Seizure frequency	X	X	X	X	X	X	X	X	X	X	X	X	X
Adaptive behavior, QoL													
Neuromotor function assessments[n]										X			
Neurocognitive development[o]													
Safety													
Adverse events	X	X	X	X	X	X	X	X	X	X	X	X	X
Concomitant medications	X	X	X	X	X	X	X	X	X	X	X	X	X
4 weeks after final dose telephone follow-up[p]													

ADAs = anti-drug antibodies; ECG = electrocardiogram; EOS = End-of-Study Visit; FU = Follow-Up Visit; GC = guanidino compound; IGF-1 = Insulin-like Growth Factor-1; IMP = investigational medicinal product; PE = physical examination; PK = pharmacokinetic(s); QoL = quality of life.
Subjects may continue treatment in the open-label portion of the study until the study drug becomes available through another means or the Investigator, Sponsor or subject determine it is no longer in the subject's interest to receive the study medication. If any subject is participating in the Blinded Follow-up when the data from the Randomized Period are locked and unblinded, the remaining visits for that subject in the Blinded Follow-up can be unblinded.
All assessments on a dosing day are to be obtained prior to dose administration unless otherwise indicated.
NOTE: Study visits will be conducted every 7 days ±1 day during the study.

[a] Vital signs include systolic and diastolic blood pressure, pulse, temperature, and respirations prior to dosing and as clinically indicated.
[b] Brief physical examination to document clinically significant changes from Baseline.
[c] Neurological examination is described in Section 8.3.
[d] Serum pregnancy test will be collected at screening and end of treatment; all other timepoints can be urine or serum tests (females of childbearing potential only)
[e] After LTE24, prior to dosing arginine and ornithine samples are required every 4 weeks but may be performed more frequently as clinically indicated at the discretion of the Investigator.
[f] Samples for determination of amino acids and ammonia will be collected prior to dosing. All amino acid and ammonia samples will be drawn after a 4-hour fast when clinically and logistically feasible. The time of the last dose of EAAs must be recorded.
[g] See Section 8.11.
[h] Samples for tryptase and complement C3 will be collected at Baseline. Samples will be shipped to the central laboratory
[i] Samples for ADAs will be collected at Baseline and pre-dose at regular intervals throughout the study according to the Schedules of Assessments. Samples will be shipped to the central laboratory.
[j] Samples for phenylbutyrate metabolite analysis will be collected at LTE01 and upon the occurrence of any hyperammonemic episode at the same time as ammonia samples are collected. Phenylbutyrate metabolite analysis samples will be shipped to the central laboratory.
[k] In the event of a hypersensitivity reaction additional tryptase, complement C3, and ADA samples will be taken at 3 and 24 hours after the start of the reaction. Samples will be shipped to the central laboratory.
[l] In the event of a hyperammonemic episode, additional phenylbutyrate metabolite samples will be taken at the same time as ammonia samples are collected. Samples will be shipped to the central laboratory
[m] Individualized Diet Management questionnaire is a record of protein prescribed and consumed. During the LTE period, protein prescribed and consumed will be collected on the 3 days prior to Visits LTE05, LTE09, and then every 12 weeks.
[n] Neuromotor assessments are detailed in Section 8.4. At visits where neuromotor assessments are conducted, the 2MWT will be the first neuromotor assessment performed. Every effort will be made to schedule neuromotor assessments on their assigned weeks; if this is not possible, these assessments may be delayed by up to 2 weeks.
[o] Required neurocognitive assessments by age are detailed in Section 8.8. Required neurocognitive assessments are to be conducted approximately every 6 months during the Follow-Up period beginning from the first blinded dose in this period. Every effort will be made to schedule neurocognitive assessments on their assigned weeks; if this is not possible, these assessments may be delayed by up to 2 weeks.
[p] The final dose telephone follow-up will occur 4 weeks after the subject receives the final dose of pegzilarginase, regardless of when that final dose is administered.

Table 7: Schedule of Assessments – Open-Label LTE40-LTE52

**Blinding**	**Open-Label (142 weeks)**												
**Visit ID**	**LTE40**	**LTE41**	**LTE42**	**LTE43**	**LTE44**	**LTE45**	**LTE46**	**LTE47**	**LTE48**	**LTE49**	**LTE50**	**LTE51**	**LTE52**
**LTE Study Day**	**274**	**281**	**288**	**295**	**302**	**309**	**316**	**323**	**330**	**337**	**344**	**351**	**358**
Drug Administration													
Open-label IMP administration	X	X	X	X	X	X	X	X	X	X	X	X	X
Clinical Assessments													
Height and weight		X				X				X			
Vital signs (prior to dosing on dosing days)[a]	X	X	X	X	X	X	X	X	X	X	X	X	X
ECG						X							
Physical examination[b]						X							
Neurological examination[c]						X							
Laboratory													
Pregnancy Test[d]		X				X				X			
Prior to dosing arginine and ornithine[e]	X				X				X				X
Guanidino compounds	X				X				X				X
Plasma amino acids and ammonia[f]						X							
Hematology, coagulation[g]						X							
Blood chemistry[g]						X							
Urinalysis[g]						X							
Growth hormone and IGF-1						X							
Tryptase, complement C3[h]													
ADAs[i]						X							

Blinding	Open-Label (142 weeks)												
**Visit ID**	**LTE40**	**LTE41**	**LTE42**	**LTE43**	**LTE44**	**LTE45**	**LTE46**	**LTE47**	**LTE48**	**LTE49**	**LTE50**	**LTE51**	**LTE52**
**LTE Study Day**	**274**	**281**	**288**	**295**	**302**	**309**	**316**	**323**	**330**	**337**	**344**	**351**	**358**
Phenylbutyrate Metabolite Analysis[j]													
Hypersensitivity reaction – additional tryptase, complement C3, ADA samples[k]													
Hyperammonemic episode – additional phenylbutyrate metabolite samples[l]													
Outcomes													
Individualized Diet Management questionnaire[m]						X							
Seizure frequency	X	X	X	X	X	X	X	X	X	X	X	X	X
Adaptive behavior, QoL									X				
Neuromotor function assessments[n]									X				
Neurocognitive development[o]									X				
Safety													
Adverse events	X	X	X	X	X	X	X	X	X	X	X	X	X
Concomitant medications	X	X	X	X	X	X	X	X	X	X	X	X	X
4 weeks after final dose telephone follow-up[p]													

ADAs = anti-drug antibodies; ECG = electrocardiogram; EOS = End-of-Study Visit; FU = Follow-Up Visit; GC = guanidino compound; IGF-1 = Insulin-like Growth Factor-1; IMP = investigational medicinal product; PE = physical examination; PK = pharmacokinetic(s); QoL = quality of life.
Subjects may continue treatment in the open-label portion of the study until the study drug becomes available through another means or the Investigator, Sponsor or subject determine it is no longer in the subject’s interest to receive the study medication. If any subject is participating in the Blinded Follow-up when the data from the Randomized Period are locked and unblinded, the remaining visits for that subject in the Blinded Follow-up can be unblinded.
All assessments on a dosing day are to be obtained prior to dose administration unless otherwise indicated.

NOTE: Study visits will be conducted every 7 days ±1 day during the study.

[a] Vital signs include systolic and diastolic blood pressure, pulse, temperature, and respirations prior to dosing and as clinically indicated.
[b] Brief physical examination to document clinically significant changes from Baseline.
[c] Neurological examination is described in Section 8.3.
[d] Serum pregnancy test will be collected at screening and end of treatment; all other timepoints can be urine or serum tests (females of childbearing potential only)
[e] After LTE24, prior to dosing arginine and ornithine samples are required every 4 weeks but may be performed more frequently as clinically indicated at the discretion of the Investigator.
[f] Samples for determination of amino acids and ammonia will be collected prior to dosing. All amino acid and ammonia samples will be drawn after a 4-hour fast when clinically and logistically feasible. The time of the last dose of EAAs must be recorded.
[g] See Section 8.11.
[h] Samples for tryptase and complement C3 will be collected at Baseline. Samples will be shipped to the central laboratory
[i] Samples for ADAs will be collected at Baseline and pre-dose at regular intervals throughout the study according to the Schedules of Assessments. Samples will be shipped to the central laboratory.
[j] Samples for phenylbutyrate metabolite analysis will be collected at LTE01 and upon the occurrence of any hyperammonemic episode at the same time as ammonia samples are collected. Phenylbutyrate metabolite analysis samples will be shipped to the central laboratory.
[k] In the event of a hypersensitivity reaction additional tryptase, complement C3, and ADA samples will be taken at 3 and 24 hours after the start of the reaction. Samples will be shipped to the central laboratory.
[l] In the event of a hyperammonemic episode, additional phenylbutyrate metabolite samples will be taken at the same time as ammonia samples are collected. Samples will be shipped to the central laboratory
[m] Individualized Diet Management questionnaire is a record of protein prescribed and consumed. During the LTE period, protein prescribed and consumed will be collected on the 3 days prior to Visits LTE05, LTE09, and then every 12 weeks.
[n] Neuromotor assessments are detailed in Section 8.4. At visits where neuromotor assessments are conducted, the 2MWT will be the first neuromotor assessment performed. Every effort will be made to schedule neuromotor assessments on their assigned weeks; if this is not possible, these assessments may be delayed by up to 2 weeks.
[o] Required neurocognitive assessments by age are detailed in Section 8.8. Required neurocognitive assessments are to be conducted approximately every 6 months during the Follow-Up period beginning from the first blinded dose in this period. Every effort will be made to schedule neurocognitive assessments on their assigned weeks; if this is not possible, these assessments may be delayed by up to 2 weeks.
[p] The final dose telephone follow-up will occur 4 weeks after the subject receives the final dose of pegzilarginase, regardless of when that final dose is administered.

Table 8: Schedule of Assessments – Open-Label LTE53-LTE65

**Blinding**	**Open-Label (142 weeks)**												
**Visit ID**	**LTE53**	**LTE54**	**LTE55**	**LTE56**	**LTE57**	**LTE58**	**LTE59**	**LTE60**	**LTE61**	**LTE62**	**LTE63**	**LTE64**	**LTE65**
**LTE Study Day**	**365**	**372**	**379**	**386**	**393**	**400**	**407**	**414**	**421**	**428**	**435**	**442**	**449**
Drug Administration													
Open-label IMP administration	X	X	X	X	X	X	X	X	X	X	X	X	X
Clinical Assessments													
Height and weight	X				X				X				X
Vital signs (prior to dosing on dosing days)[a]	X	X	X	X	X	X	X	X	X	X	X	X	X
ECG					X								
Physical examination[b]					X								
Neurological examination[c]					X								
Laboratory													
Pregnancy Test[d]	X				X				X				X
Prior to dosing arginine and ornithine[e]				X				X				X	
Guanidino compounds				X				X				X	
Plasma amino acids and ammonia[f]					X								
Hematology, coagulation[g]					X								
Blood chemistry[g]					X								
Urinalysis[g]					X								
Growth hormone and IGF-1					X								
Tryptase, complement C3[h]													
ADAs[i]					X								

Blinding	Open-Label (142 weeks)												
**Visit ID**	**LTE53**	**LTE54**	**LTE55**	**LTE56**	**LTE57**	**LTE58**	**LTE59**	**LTE60**	**LTE61**	**LTE62**	**LTE63**	**LTE64**	**LTE65**
**LTE Study Day**	**365**	**372**	**379**	**386**	**393**	**400**	**407**	**414**	**421**	**428**	**435**	**442**	**449**
Phenylbutyrate Metabolite Analysis[j]													
Hypersensitivity reaction – additional tryptase, complement C3, ADA samples[k]													
Hyperammonemic episode – additional phenylbutyrate metabolite samples[l]													
Outcomes													
Individualized Diet Management questionnaire[m]					X								
Seizure frequency	X	X	X	X	X	X	X	X	X	X	X	X	X
Adaptive behavior, QoL													
Neuromotor function assessments[n]								X					
Neurocognitive development[o]													
Safety													
Adverse events	X	X	X	X	X	X	X	X	X	X	X	X	X
Concomitant medications	X	X	X	X	X	X	X	X	X	X	X	X	X
4 weeks after final dose telephone follow-up[p]													

ADAs = anti-drug antibodies; ECG = electrocardiogram; EOS = End-of-Study Visit; FU = Follow-Up Visit; GC = guanidino compound; IGF-1 = Insulin-like Growth Factor-1; IMP = investigational medicinal product; PE = physical examination; LTE = long-term extension; PK = pharmacokinetic(s); QoL = quality of life.
Subjects may continue treatment in the open-label portion of the study until the study drug becomes available through another means or the Investigator, Sponsor or subject determine it is no longer in the subject's interest to receive the study medication. If any subject is participating in the Blinded Follow-up when the data from the Randomized Period are locked and unblinded, the remaining visits for that subject in the Blinded Follow-up can be unblinded.
All assessments on a dosing day are to be obtained prior to dose administration unless otherwise indicated.
NOTE: Study visits will be conducted every 7 days ±1 day during the study.

[a] Vital signs include systolic and diastolic blood pressure, pulse, temperature, and respirations prior to dosing and as clinically indicated.
[b] Brief physical examination to document clinically significant changes from Baseline.
[c] Neurological examination is described in Section 8.3.
[d] Serum pregnancy test will be collected at screening and end of treatment; all other timepoints can be urine or serum tests (females of childbearing potential only)
[e] After LTE24, prior to dosing arginine and ornithine samples are required every 4 weeks but may be performed more frequently as clinically indicated at the discretion of the Investigator.
[f] Samples for determination of amino acids and ammonia will be collected prior to dosing. All amino acid and ammonia samples will be drawn after a 4-hour fast when clinically and logistically feasible. The time of the last dose of EAAs must be recorded.
[g] See Section 8.11
[h] Samples for tryptase and complement C3 will be collected at Baseline. Samples will be shipped to the central laboratory
[i] Samples for ADAs will be collected at Baseline and pre-dose at regular intervals throughout the study according to the Schedules of Assessments. Samples will be shipped to the central laboratory.
[j] Samples for phenylbutyrate metabolite analysis will be collected at LTE01 and upon the occurrence of any hyperammonemic episode at the same time as ammonia samples are collected. Phenylbutyrate metabolite analysis samples will be shipped to the central laboratory.
[k] In the event of a hypersensitivity reaction additional tryptase, complement C3, and ADA samples will be taken at 3 and 24 hours after the start of the reaction. Samples will be shipped to the central laboratory.
[l] In the event of a hyperammonemic episode, additional phenylbutyrate metabolite samples will be taken at the same time as ammonia samples are collected. Samples will be shipped to the central laboratory
[m] Individualized Diet Management questionnaire is a record of protein prescribed and consumed. During the LTE period, protein prescribed and consumed will be collected on the 3 days prior to Visits LTE05, LTE09, and then every 12 weeks.
[n] Neuromotor assessments are detailed in Section 8.4. At visits where neuromotor assessments are conducted, the 2MWT will be the first neuromotor assessment performed. Every effort will be made to schedule neuromotor assessments on their assigned weeks; if this is not possible, these assessments may be delayed by up to 2 weeks.
[o] Required neurocognitive assessments by age are detailed in Section 8.8. Required neurocognitive assessments are to be conducted approximately every 6 months during the Follow-Up period beginning from the first blinded dose in this period. Every effort will be made to schedule neurocognitive assessments on their assigned weeks; if this is not possible, these assessments may be delayed by up to 2 weeks.
[p] The final dose telephone follow-up will occur 4 weeks after the subject receives the final dose of pegzilarginase, regardless of when that final dose is administered

Table 9: Schedule of Assessments – Open-Label LTE66-LTE78

**Blinding**	**Open-Label (142 weeks)**												
**Visit ID**	**LTE66**	**LTE67**	**LTE68**	**LTE69**	**LTE70**	**LTE71**	**LTE72**	**LTE73**	**LTE74**	**LTE75**	**LTE76**	**LTE77**	**LTE78**
**LTE Study Day**	**456**	**463**	**470**	**477**	**484**	**491**	**498**	**505**	**512**	**519**	**526**	**533**	**540**
Drug Administration													
Open-label IMP administration	X	X	X	X	X	X	X	X	X	X	X	X	X
Clinical Assessments													
Height and weight				X				X				X	
Vital signs (prior to dosing on dosing days)[a]	X	X	X	X	X	X	X	X	X	X	X	X	X
ECG				X									
Physical examination[b]				X									
Neurological examination[c]				X									
Laboratory													
Pregnancy Test[d]				X				X				X	
Prior to dosing arginine and ornithine[e]			X				X				X		
Guanidino compounds			X				X				X		
Plasma amino acids and ammonia[f]				X									
Hematology, coagulation[g]				X									
Blood chemistry[g]				X									
Urinalysis[g]				X									
Growth hormone and IGF-1				X									
Tryptase, complement C3[h]													
ADAs[i]				X									

Blinding	Open-Label (142 weeks)												
**Visit ID**	**LTE66**	**LTE67**	**LTE68**	**LTE69**	**LTE70**	**LTE71**	**LTE72**	**LTE73**	**LTE74**	**LTE75**	**LTE76**	**LTE77**	**LTE78**
**LTE Study Day**	**456**	**463**	**470**	**477**	**484**	**491**	**498**	**505**	**512**	**519**	**526**	**533**	**540**
Phenylbutyrate Metabolite Analysis[j]													
Hypersensitivity reaction – additional tryptase, complement C3, ADA samples[k]													
Hyperammonemic episode – additional phenylbutyrate metabolite samples[l]													
Outcomes													
Individualized Diet Management questionnaire[m]				X									
Seizure frequency	X	X	X	X	X	X	X	X	X	X	X	X	X
Adaptive behavior, QoL													
Neuromotor function assessments[n]							X						
Neurocognitive development[o]							X						
Safety													
Adverse events	X	X	X	X	X	X	X	X	X	X	X	X	X
Concomitant medications	X	X	X	X	X	X	X	X	X	X	X	X	X
4 weeks after final dose telephone follow-up[p]													

ADAs = anti-drug antibodies; ECG = electrocardiogram; EOS = End-of-Study Visit; FU = Follow-Up Visit; GC = guanidino compound; IGF 1 = Insulin-like Growth Factor-1; IMP = investigational medicinal product; PE = physical examination; PK = pharmacokinetic(s); QoL = quality of life.

Subjects may continue treatment in the open-label portion of the study until the study drug becomes available through another means or the Investigator, Sponsor or subject determine it is no longer in the subject's interest to receive the study medication. If any subject is participating in the Blinded Follow-up when the data from the Randomized Period are locked and unblinded, the remaining visits for that subject in the Blinded Follow-up can be unblinded.

All assessments on a dosing day are to be obtained prior to dose administration unless otherwise indicated.

NOTE: Study visits will be conducted every 7 days ±1 day during the study.

[a] Vital signs include systolic and diastolic blood pressure, pulse, temperature, and respirations prior to dosing and as clinically indicated.
[b] Brief physical examination to document clinically significant changes from Baseline.
[c] Neurological examination is described in Section 8.3.
[d] Serum pregnancy test will be collected at screening and end of treatment; all other timepoints can be urine or serum tests (females of childbearing potential only)
[e] After LTE24, prior to dosing arginine and ornithine samples are required every 4 weeks but may be performed more frequently as clinically indicated at the discretion of the Investigator.
[f] Samples for determination of amino acids and ammonia will be collected prior to dosing. All amino acid and ammonia samples will be drawn after a 4-hour fast when clinically and logistically feasible. The time of the last dose of EAAs must be recorded.
[g] See Section 8.11.
[h] Samples for tryptase and complement C3 will be collected at Baseline. Samples will be shipped to the central laboratory.
[i] Samples for ADAs will be collected at Baseline and pre-dose at regular intervals throughout the study according to the Schedules of Assessments. Samples will be shipped to the central laboratory.
[j] Samples for phenylbutyrate metabolite analysis will be collected at LTE01 and upon the occurrence of any hyperammonemic episode at the same time as ammonia samples are collected. Phenylbutyrate metabolite analysis samples will be shipped to the central laboratory.
[k] In the event of a hypersensitivity reaction additional tryptase, complement C3, and ADA samples will be taken at 3 and 24 hours after the start of the reaction. Samples will be shipped to the central laboratory.
[l] In the event of a hyperammonemic episode, additional phenylbutyrate metabolite samples will be taken at the same time as ammonia samples are collected. Samples will be shipped to the central laboratory
[m] Individualized Diet Management questionnaire is a record of protein prescribed and consumed. During the LTE period, protein prescribed and consumed will be collected on the 3 days prior to Visits LTE05, LTE09, and then every 12 weeks.
[n] Neuromotor assessments are detailed in Section 8.4. At visits where neuromotor assessments are conducted, the 2MWT will be the first neuromotor assessment performed. Every effort will be made to schedule neuromotor assessments on their assigned weeks; if this is not possible, these assessments may be delayed by up to 2 weeks.
[o] Required neurocognitive assessments by age are detailed in Section 8.8. Required neurocognitive assessments are to be conducted approximately every 6 months during the Follow-Up period beginning from the first blinded dose in this period. Every effort will be made to schedule neurocognitive assessments on their assigned weeks; if this is not possible, these assessments may be delayed by up to 2 weeks.
[p] The final dose telephone follow-up will occur 4 weeks after the subject receives the final dose of pegzilarginase, regardless of when that final dose is administered.

Table 10: Schedule of Assessments – Open-Label LTE79-LTE91

Blinding	Open-Label (142 weeks)												
**Visit ID**	**LTE79**	**LTE80**	**LTE81**	**LTE82**	**LTE83**	**LTE84**	**LTE85**	**LTE86**	**LTE87**	**LTE88**	**LTE89**	**LTE90**	**LTE91**
**LTE Study Day**	**547**	**554**	**561**	**568**	**575**	**582**	**589**	**596**	**603**	**610**	**617**	**624**	**631**
Drug Administration													
Open-label IMP administration	X	X	X	X	X	X	X	X	X	X	X	X	X
Clinical Assessments													
Height and weight			X				X				X		
Vital signs (prior to dosing on dosing days)[a]	X	X	X	X	X	X	X	X	X	X	X	X	X
ECG			X										
Physical examination[b]			X										
Neurological examination[c]			X										
Laboratory													
Pregnancy Test[d]			X				X				X		
Prior to dosing arginine and ornithine[e]		X				X				X			
Guanidino compounds		X				X				X			
Plasma amino acids and ammonia[f]			X										
Hematology, coagulation[g]			X										
Blood chemistry[g]			X										
Urinalysis[g]			X										
Growth hormone and IGF-1			X										
Tryptase, complement C3[h]													
ADAs[i]			X										

Blinding	Open-Label (142 weeks)												
**Visit ID**	**LTE79**	**LTE80**	**LTE81**	**LTE82**	**LTE83**	**LTE84**	**LTE85**	**LTE86**	**LTE87**	**LTE88**	**LTE89**	**LTE90**	**LTE91**
**LTE Study Day**	**547**	**554**	**561**	**568**	**575**	**582**	**589**	**596**	**603**	**610**	**617**	**624**	**631**
Phenylbutyrate Metabolite Analysis[j]													
Hypersensitivity reaction – additional tryptase, complement C3, ADA samples[k]													
Hyperammonemic episode – additional phenylbutyrate metabolite samples[l]													
Outcomes													
Individualized Diet Management questionnaire[m]			X										
Seizure frequency	X	X	X	X	X	X	X	X	X	X	X	X	X
Adaptive behavior, QoL													
Neuromotor function assessments[n]						X							
Neurocognitive development[o]													
Safety													
Adverse events	X	X	X	X	X	X	X	X	X	X	X	X	X
Concomitant medications	X	X	X	X	X	X	X	X	X	X	X	X	X
4 weeks after final dose telephone follow-up[p]													

ADAs = anti-drug antibodies; ECG = electrocardiogram; EOS = End-of-Study Visit; FU = Follow-Up Visit; GC = guanidino compound; IGF-1 = Insulin-like Growth Factor-1; IMP = investigational medicinal product; PE = physical examination; LTE = long-term extension; PK = pharmacokinetic(s); QoL = quality of life.

Subjects may continue treatment in the open-label portion of the study until the study drug becomes available through another means or the Investigator, Sponsor or subject determine it is no longer in the subject's interest to receive the study medication. If any subject is participating in the Blinded Follow-up when the data from the Randomized Period are locked and unblinded, the remaining visits for that subject in the Blinded Follow-up can be unblinded.

All assessments on a dosing day are to be obtained prior to dose administration unless otherwise indicated.

NOTE: Study visits will be conducted every 7 days ±1 day during the study.

[a] Vital signs include systolic and diastolic blood pressure, pulse, temperature, and respirations prior to dosing and as clinically indicated.
[b] Brief physical examination to document clinically significant changes from Baseline.
[c] Neurological examination is described in Section 8.3.
[d] Serum pregnancy test will be collected at screening and end of treatment; all other timepoints can be urine or serum tests (females of childbearing potential only)
[e] After LTE24, prior to dosing arginine and ornithine samples are required every 4 weeks but may be performed more frequently as clinically indicated at the discretion of the Investigator.
[f] Samples for determination of amino acids and ammonia will be collected prior to dosing. All amino acid and ammonia samples will be drawn after a 4-hour fast when clinically and logistically feasible. The time of the last dose of EAAs must be recorded.
[g] See Section 8.11.
[h] Samples for tryptase and complement C3 will be collected at Baseline. Samples will be shipped to the central laboratory.
[i] Samples for ADAs will be collected at Baseline and pre-dose at regular intervals throughout the study according to the Schedules of Assessments. Samples will be shipped to the central laboratory.
[j] Samples for phenylbutyrate metabolite analysis will be collected at LTE01 and upon the occurrence of any hyperammonemic episode at the same time as ammonia samples are collected. Phenylbutyrate metabolite analysis samples will be shipped to the central laboratory.
[k] In the event of a hypersensitivity reaction additional tryptase, complement C3, and ADA samples will be taken at 3 and 24 hours after the start of the reaction. Samples will be shipped to the central laboratory.
[l] In the event of a hyperammonemic episode, additional phenylbutyrate metabolite samples will be taken at the same time as ammonia samples are collected. Samples will be shipped to the central laboratory
[m] Individualized Diet Management questionnaire is a record of protein prescribed and consumed. During the LTE period, protein prescribed and consumed will be collected on the 3 days prior to Visits LTE05, LTE09, and then every 12 weeks.
[n] Neuromotor assessments are detailed in Section 8.4. At visits where neuromotor assessments are conducted, the 2MWT will be the first neuromotor assessment performed. Every effort will be made to schedule neuromotor assessments on their assigned weeks; if this is not possible, these assessments may be delayed by up to 2 weeks.
[o] Required neurocognitive assessments by age are detailed in Section 8.8. Required neurocognitive assessments are to be conducted approximately every 6 months during the Follow-Up period beginning from the first blinded dose in this period. Every effort will be made to schedule neurocognitive assessments on their assigned weeks; if this is not possible, these assessments may be delayed by up to 2 weeks.
[p] The final dose telephone follow-up will occur 4 weeks after the subject receives the final dose of pegzilarginase, regardless of when that final dose is administered.

Table 11: Schedule of Assessments – Open-Label LTE92-LTE104

**Blinding**	**Open-Label (142 weeks)**												
**Visit ID**	**LTE92**	**LTE93**	**LTE94**	**LTE95**	**LTE96**	**LTE97**	**LTE98**	**LTE99**	**LTE100**	**LTE101**	**LTE102**	**LTE103**	**LTE104**
**LTE Study Day**	**638**	**645**	**652**	**659**	**666**	**673**	**680**	**687**	**694**	**701**	**708**	**715**	**722**
Drug Administration													
Open-label IMP administration	X	X	X	X	X	X	X	X	X	X	X	X	X
Clinical Assessments													
Height and weight		X				X				X			
Vital signs (prior to dosing on dosing days)[a]	X	X	X	X	X	X	X	X	X	X	X	X	X
ECG		X											
Physical examination[b]		X											
Neurological examination[c]		X											
Laboratory													
Pregnancy Test[d]		X				X				X			
Prior to dosing arginine and ornithine[e]	X				X				X				X
Guanidino compounds	X				X				X				X
Plasma amino acids and ammonia[f]		X											
Hematology, coagulation[g]		X											
Blood chemistry[g]		X											
Urinalysis[g]		X											
Growth hormone and IGF-1		X											
Tryptase, complement C3[h]													
ADAs[i]		X											

Blinding	Open-Label (142 weeks)												
**Visit ID**	**LTE92**	**LTE93**	**LTE94**	**LTE95**	**LTE96**	**LTE97**	**LTE98**	**LTE99**	**LTE100**	**LTE101**	**LTE102**	**LTE103**	**LTE104**
**LTE Study Day**	**638**	**645**	**652**	**659**	**666**	**673**	**680**	**687**	**694**	**701**	**708**	**715**	**722**
Phenylbutyrate Metabolite Analysis[j]													
Hypersensitivity reaction – additional tryptase, complement C3, ADA samples[k]													
Hyperammonemic episode – additional phenylbutyrate metabolite samples[l]													
Outcomes													
Individualized Diet Management questionnaire[m]		X											
Seizure frequency	X	X	X	X	X	X	X	X	X	X	X	X	X
Adaptive behavior, QoL					X								
Neuromotor function assessments[n]					X								
Neurocognitive development[o]					X								
Safety													
Adverse events	X	X	X	X	X	X	X	X	X	X	X	X	X
Concomitant medications	X	X	X	X	X	X	X	X	X	X	X	X	X
4 weeks after final dose telephone follow-up[p]													

ADAs = anti-drug antibodies; ECG = electrocardiogram; EOS = End-of-Study Visit; FU = Follow-Up Visit; GC = guanidino compound; IGF-1 = Insulin-like Growth Factor-1; IMP = investigational medicinal product; PE = physical examination; LTE = long-term extension; PK = pharmacokinetic(s); QoL = quality of life.

Subjects may continue treatment in the open-label portion of the study until the study drug becomes available through another means or the Investigator, Sponsor or subject determine it is no longer in the subject's interest to receive the study medication. If any subject is participating in the Blinded Follow-up when the data from the Randomized Period are locked and unblinded, the remaining visits for that subject in the Blinded Follow-up can be unblinded.

All assessments on a dosing day are to be obtained prior to dose administration unless otherwise indicated.

NOTE: Study visits will be conducted every 7 days ±1 day during the study.

[a] Vital signs include systolic and diastolic blood pressure, pulse, temperature, and respirations prior to dosing and as clinically indicated.
[b] Brief physical examination to document clinically significant changes from Baseline.
[c] Neurological examination is described in Section 8.3.
[d] Serum pregnancy test will be collected at screening and end of treatment; all other timepoints can be urine or serum tests (females of childbearing potential only)
[e] After LTE24, prior to dosing arginine and ornithine samples are required every 4 weeks but may be performed more frequently as clinically indicated at the discretion of the Investigator.
[f] Samples for determination of amino acids and ammonia will be collected prior to dosing. All amino acid and ammonia samples will be drawn after a 4-hour fast when clinically and logistically feasible. The time of the last dose of EAAs must be recorded.
[g] See Section 8.11.
[h] Samples for tryptase and complement C3 will be collected at Baseline. Samples will be shipped to the central laboratory.
[i] Samples for ADAs will be collected at Baseline and pre-dose at regular intervals throughout the study according to the Schedules of Assessments. Samples will be shipped to the central laboratory.
[j] Samples for phenylbutyrate metabolite analysis will be collected at LTE01 and upon the occurrence of any hyperammonemic episode at the same time as ammonia samples are collected. Phenylbutyrate metabolite analysis samples will be shipped to the central laboratory.
[k] In the event of a hypersensitivity reaction additional tryptase, complement C3, and ADA samples will be taken at 3 and 24 hours after the start of the reaction. Samples will be shipped to the central laboratory.
[l] In the event of a hyperammonemic episode, additional phenylbutyrate metabolite samples will be taken at the same time as ammonia samples are collected. Samples will be shipped to the central laboratory
[m] Individualized Diet Management questionnaire is a record of protein prescribed and consumed. During the LTE period, protein prescribed and consumed will be collected on the 3 days prior to Visits LTE05, LTE09, and then every 12 weeks.
[n] Neuromotor assessments are detailed in Section 8.4. At visits where neuromotor assessments are conducted, the 2MWT will be the first neuromotor assessment performed. Every effort will be made to schedule neuromotor assessments on their assigned weeks; if this is not possible, these assessments may be delayed by up to 2 weeks.
[o] Required neurocognitive assessments by age are detailed in Section 8.8. Required neurocognitive assessments are to be conducted approximately every 6 months during the Follow-Up period beginning from the first blinded dose in this period. Every effort will be made to schedule neurocognitive assessments on their assigned weeks; if this is not possible, these assessments may be delayed by up to 2 weeks.
[p] The final dose telephone follow-up will occur 4 weeks after the subject receives the final dose of pegzilarginase, regardless of when that final dose is administered.

Table 12: Schedule of Assessments – Open-Label LTE105-LTE117

Blinding	Open-Label (142 weeks)												
**Visit ID**	**LTE 105**	**LTE 106**	**LTE 107**	**LTE108**	**LTE109**	**LTE110**	**LTE111**	**LTE112**	**LTE113**	**LTE114**	**LTE115**	**LTE116**	**LTE117**
**LTE Study Day**	**729**	**736**	**743**	**750**	**757**	**764**	**771**	**778**	**785**	**792**	**799**	**806**	**813**
Drug Administration													
Open-label IMP administration	X	X	X	X	X	X	X	X	X	X	X	X	X
Clinical Assessments													
Height and weight	X				X				X				X
Vital signs (prior to dosing on dosing days)[a]	X	X	X	X	X	X	X	X	X	X	X	X	X
ECG	X												X
Physical examination[b]	X												X
Neurological examination[c]	X												X
Laboratory													
Pregnancy Test[d]	X				X				X				X
Prior to dosing arginine and ornithine[e]				X				X				X	
Guanidino compounds				X				X				X	
Plasma amino acids and ammonia[f]	X												X
Hematology, coagulation[g]	X												X
Blood chemistry[g]	X												X
Urinalysis[g]	X												X
Growth hormone and IGF-1	X												X
Tryptase, complement C3[h]									X				
ADAs[i]	X												X

Blinding	Open-Label (142 weeks)												
Visit ID	LTE 105	LTE 106	LTE 107	LTE108	LTE109	LTE110	LTE111	LTE112	LTE113	LTE114	LTE115	LTE116	LTE117
LTE Study Day	729	736	743	750	757	764	771	778	785	792	799	806	813
Phenylbutyrate Metabolite Analysis[j]													
Hypersensitivity reaction – additional tryptase, complement C3, ADA samples[k]													
Hyperammonemic episode – additional phenylbutyrate metabolite samples[l]													
Outcomes													
Individualized Diet Management questionnaire[m]	X												X
Seizure frequency	X	X	X	X	X	X	X	X	X	X	X	X	X
Adaptive behavior, QoL													
Neuromotor function assessments[n]													
Neurocognitive development[o]													
Safety													
Adverse events	X	X	X	X	X	X	X	X	X	X	X	X	X
Concomitant medications	X	X	X	X	X	X	X	X	X	X	X	X	X
4 weeks after final dose telephone follow-up[p]													

ADAs = anti-drug antibodies; ECG = electrocardiogram; EOS = End-of-Study Visit; FU = Follow-Up Visit; GC = guanidino compound; IGF-1 = Insulin-like Growth Factor-1; IMP = investigational medicinal product; PE = physical examination; PK = pharmacokinetic(s); QoL = quality of life.

Subjects may continue treatment in the open-label portion of the study until the study drug becomes available through another means or the Investigator, Sponsor or subject determine it is no longer in the subject's interest to receive the study medication. If any subject is participating in the Blinded Follow-up when the data from the Randomized Period are locked and unblinded, the remaining visits for that subject in the Blinded Follow-up can be unblinded.

All assessments on a dosing day are to be obtained prior to dose administration unless otherwise indicated.

NOTE: Study visits will be conducted every 7 days ±1 day during the study.

[a] Vital signs include systolic and diastolic blood pressure, pulse, temperature, and respirations prior to dosing and as clinically indicated.
[b] Brief physical examination to document clinically significant changes from Baseline.
[c] Neurological examination is described in Section 8.3.
[d] Serum pregnancy test will be collected at screening and end of treatment; all other timepoints can be urine or serum tests (females of childbearing potential only)
[e] After LTE24, prior to dosing arginine and ornithine samples are required every 4 weeks but may be performed more frequently as clinically indicated at the discretion of the Investigator.
[f] Samples for determination of amino acids and ammonia will be collected prior to dosing. All amino acid and ammonia samples will be drawn after a 4-hour fast when clinically and logistically feasible. The time of the last dose of EAAs must be recorded.
[g] See Section 8.11.
[h] Samples for tryptase and complement C3 will be collected at Baseline. Samples will be shipped to the central laboratory.
[i] Samples for ADAs will be collected at Baseline and pre-dose at regular intervals throughout the study according to the Schedules of Assessments. Samples will be shipped to the central laboratory.
[j] Samples for phenylbutyrate metabolite analysis will be collected at LTE01 and upon the occurrence of any hyperammonemic episode at the same time as ammonia samples are collected. Phenylbutyrate metabolite analysis samples will be shipped to the central laboratory.
[k] In the event of a hypersensitivity reaction additional tryptase, complement C3, and ADA samples will be taken at 3 and 24 hours after the start of the reaction. Samples will be shipped to the central laboratory.
[l] In the event of a hyperammonemic episode, additional phenylbutyrate metabolite samples will be taken at the same time as ammonia samples are collected. Samples will be shipped to the central laboratory
[m] Individualized Diet Management questionnaire is a record of protein prescribed and consumed. During the LTE period, protein prescribed and consumed will be collected on the 3 days prior to Visits LTE05, LTE09, and then every 12 weeks.
[n] Neuromotor assessments are detailed in Section 8.4. At visits where neuromotor assessments are conducted, the 2MWT will be the first neuromotor assessment performed. Every effort will be made to schedule neuromotor assessments on their assigned weeks; if this is not possible, these assessments may be delayed by up to 2 weeks.
[o] Required neurocognitive assessments by age are detailed in Section 8.8. Required neurocognitive assessments are to be conducted approximately every 6 months during the Follow-Up period beginning from the first blinded dose in this period. Every effort will be made to schedule neurocognitive assessments on their assigned weeks; if this is not possible, these assessments may be delayed by up to 2 weeks.
[p] The final dose telephone follow-up will occur 4 weeks after the subject receives the final dose of pegzilarginase, regardless of when that final dose is administered.

Table 13: Schedule of Assessments – Open-Label LTE118-LTE130

**Blinding**	**Open-Label (142 weeks)**												
**Visit ID**	**LTE118**	**LTE119**	**LTE120**	**LTE121**	**LTE122**	**LTE123**	**LTE124**	**LTE125**	**LTE126**	**LTE127**	**LTE128**	**LTE 129**	**LTE 130**
**LTE Study Day**	**820**	**827**	**834**	**841**	**848**	**855**	**862**	**869**	**876**	**883**	**890**	**897**	**904**
Drug Administration													
Open-label IMP administration	X	X	X	X	X	X	X	X	X	X	X	X	X
Clinical Assessments													
Height and weight				X				X				X	
Vital signs (prior to dosing on dosing days)[a]	X	X	X	X	X	X	X	X	X	X	X	X	X
ECG												X	
Physical examination[b]												X	
Neurological examination[c]												X	
Laboratory													
Pregnancy Test[d]				X				X				X	
Prior to dosing arginine and ornithine[e]			X				X				X		
Guanidino compounds			X				X				X		
Plasma amino acids and ammonia[f]												X	
Hematology, coagulation[g]												X	
Blood chemistry[g]												X	
Urinalysis[g]												X	
Growth hormone and IGF-1												X	
Tryptase, complement C3[h]													
ADAs[i]												X	

Blinding	Open-Label (142 weeks)												
**Visit ID**	**LTE118**	**LTE119**	**LTE120**	**LTE121**	**LTE122**	**LTE123**	**LTE124**	**LTE125**	**LTE126**	**LTE127**	**LTE128**	**LTE 129**	**LTE 130**
**LTE Study Day**	**820**	**827**	**834**	**841**	**848**	**855**	**862**	**869**	**876**	**883**	**890**	**897**	**904**
Phenylbutyrate Metabolite Analysis[j]													
Hypersensitivity reaction – additional tryptase, complement C3, ADA samples[k]													
Hyperammonemic episode – additional phenylbutyrate metabolite samples[l]													
Outcomes													
Individualized Diet Management questionnaire[m]												X	
Seizure frequency	X	X	X	X	X	X	X	X	X	X	X	X	X
Adaptive behavior, QoL													
Neuromotor function assessments[n]			X										
Neurocognitive development[o]													
Safety													
Adverse events	X	X	X	X	X	X	X	X	X	X	X	X	X
Concomitant medications	X	X	X	X	X	X	X	X	X	X	X	X	X
4 weeks after final dose telephone follow-up[p]													

ADAs = anti-drug antibodies; ECG = electrocardiogram; EOS = End-of-Study Visit; FU = Follow-Up Visit; GC = guanidino compound; IGF-1 = Insulin-like Growth Factor-1; IMP = investigational medicinal product; LTE = long-term extension; PE = physical examination; PK = pharmacokinetic(s); QoL = quality of life.

Subjects may continue treatment in the open-label portion of the study until the study drug becomes available through another means or the Investigator, Sponsor or subject determine it is no longer in the subject’s interest to receive the study medication. If any subject is participating in the Blinded Follow-up when the data from the Randomized Period are locked and unblinded, the remaining visits for that subject in the Blinded Follow-up can be unblinded.

All assessments on a dosing day are to be obtained prior to dose administration unless otherwise indicated.

NOTE: Study visits will be conducted every 7 days ±1 day during the study.

[a] Vital signs include systolic and diastolic blood pressure, pulse, temperature, and respirations prior to dosing and as clinically indicated.
[b] Brief physical examination to document clinically significant changes from Baseline.
[c] Neurological examination is described in Section 8.3.
[d] Serum pregnancy test will be collected at screening and end of treatment; all other timepoints can be urine or serum tests (females of childbearing potential only)
[e] After LTE24, prior to dosing arginine and ornithine samples are required every 4 weeks but may be performed more frequently as clinically indicated at the discretion of the Investigator.
[f] Samples for determination of amino acids and ammonia will be collected prior to dosing. All amino acid and ammonia samples will be drawn after a 4-hour fast when clinically and logistically feasible. The time of the last dose of EAAs must be recorded.
[g] See Section 8.11.
[h] Samples for tryptase and complement C3 will be collected at Baseline. Samples will be shipped to the central laboratory.
[i] Samples for ADAs will be collected at Baseline and pre-dose at regular intervals throughout the study according to the Schedules of Assessments. Samples will be shipped to the central laboratory.
[j] Samples for phenylbutyrate metabolite analysis will be collected at LTE01 and upon the occurrence of any hyperammonemic episode at the same time as ammonia samples are collected. Phenylbutyrate metabolite analysis samples will be shipped to the central laboratory.
[k] In the event of a hypersensitivity reaction additional tryptase, complement C3, and ADA samples will be taken at 3 and 24 hours after the start of the reaction. Samples will be shipped to the central laboratory.
[l] In the event of a hyperammonemic episode, additional phenylbutyrate metabolite samples will be taken at the same time as ammonia samples are collected. Samples will be shipped to the central laboratory
[m] Individualized Diet Management questionnaire is a record of protein prescribed and consumed. During the LTE period, protein prescribed and consumed will be collected on the 3 days prior to Visits LTE05, LTE09, and then every 12 weeks.
[n] Neuromotor assessments are detailed in Section 8.4. At visits where neuromotor assessments are conducted, the 2MWT will be the first neuromotor assessment performed. Every effort will be made to schedule neuromotor assessments on their assigned weeks; if this is not possible, these assessments may be delayed by up to 2 weeks.
[o] Required neurocognitive assessments by age are detailed in Section 8.8. Required neurocognitive assessments are to be conducted approximately every 6 months during the Follow-Up period beginning from the first blinded dose in this period. Every effort will be made to schedule neurocognitive assessments on their assigned weeks; if this is not possible, these assessments may be delayed by up to 2 weeks.
[p] The final dose telephone follow-up will occur 4 weeks after the subject receives the final dose of pegzilarginase, regardless of when that final dose is administered.

Table 14: Schedule of Assessments – Open-Label LTE131-LTE143

**Blinding**	**Open-Label (142 weeks)**												
**Visit ID**	**LTE131**	**LTE132**	**LTE133**	**LTE134**	**LTE135**	**LTE136**	**LTE137**	**LTE138**	**LTE139**	**LTE140**	**LTE141**	**LTE142**	**LTE143**
**LTE Study Day**	**911**	**918**	**925**	**932**	**939**	**946**	**953**	**960**	**967**	**974**	**981**	**988**	**995**
Drug Administration													
Open-label IMP administration	X	X	X	X	X	X	X	X	X	X	X	X	X
Clinical Assessments													
Height and weight			X				X				X		
Vital signs (prior to dosing on dosing days)[a]	X	X	X	X	X	X	X	X	X	X	X	X	X
ECG											X		
Physical examination[b]											X		
Neurological examination[c]											X		
Laboratory													
Pregnancy Test[d]			X				X				X		
Prior to dosing arginine and ornithine[e]		X				X				X			
Guanidino compounds		X				X				X			
Plasma amino acids and ammonia[f]											X		
Hematology, coagulation[g]											X		
Blood chemistry[g]											X		
Urinalysis[g]											X		
Growth hormone and IGF-1											X		

Blinding	Open-Label (142 weeks)												
**Visit ID**	**LTE131**	**LTE132**	**LTE133**	**LTE134**	**LTE135**	**LTE136**	**LTE137**	**LTE138**	**LTE139**	**LTE140**	**LTE141**	**LTE142**	**LTE143**
**LTE Study Day**	**911**	**918**	**925**	**932**	**939**	**946**	**953**	**960**	**967**	**974**	**981**	**988**	**995**
Tryptase, complement C3[h]													
ADAs[i]											X		
Phenylbutyrate Metabolite Analysis[j]													
Hypersensitivity reaction – additional tryptase, complement C3, ADA samples[k]													
Hyperammonemic episode – additional phenylbutyrate metabolite samples[l]													
Outcomes													
Individualized Diet Management questionnaire[m]											X		
Seizure frequency	X	X	X	X	X	X	X	X	X	X	X	X	X
Adaptive behavior, QoL													
Neuromotor function assessments[n]													
Neurocognitive development[o]													
Safety													
Adverse events	X	X	X	X	X	X	X	X	X	X	X	X	X
Concomitant medications	X	X	X	X	X	X	X	X	X	X	X	X	X
4 weeks after final dose telephone follow-up[p]													

ADAs = anti-drug antibodies; ECG = electrocardiogram; EOS = End-of-Study Visit; FU = Follow-Up Visit; GC = guanidino compound; IGF-1 = Insulin-like Growth Factor-1; IMP = investigational medicinal product; PE = physical examination; PK = pharmacokinetic(s); QoL = quality of life.
Subjects may continue treatment in the open-label portion of the study until the study drug becomes available through another means or the Investigator, Sponsor or subject determine it is no longer in the subject's interest to receive the study medication. If any subject is participating in the Blinded Follow-up when the data from the Randomized Period are locked and unblinded, the remaining visits for that subject in the Blinded Follow-up can be unblinded.
All assessments on a dosing day are to be obtained prior to dose administration unless otherwise indicated.
NOTE: Study visits will be conducted every 7 days ±1 day during the study.

[a] Vital signs include systolic and diastolic blood pressure, pulse, temperature, and respirations prior to dosing and as clinically indicated.
[b] Brief physical examination to document clinically significant changes from Baseline.
[c] Neurological examination is described in Section 8.3.
[d] Serum pregnancy test will be collected at screening and end of treatment; all other timepoints can be urine or serum tests (females of childbearing potential only)
[e] After LTE24, prior to dosing arginine and ornithine samples are required every 4 weeks but may be performed more frequently as clinically indicated at the discretion of the Investigator.
[f] Samples for determination of amino acids and ammonia will be collected prior to dosing. All amino acid and ammonia samples will be drawn after a 4-hour fast when clinically and logistically feasible. The time of the last dose of EAAs must be recorded.
[g] See Section 8.11.
[h] Samples for tryptase and complement C3 will be collected at Baseline. Samples will be shipped to the central laboratory.
[i] Samples for ADAs will be collected at Baseline and pre-dose at regular intervals throughout the study according to the Schedules of Assessments. Samples will be shipped to the central laboratory.
[j] Samples for phenylbutyrate metabolite analysis will be collected at LTE01 and upon the occurrence of any hyperammonemic episode at the same time as ammonia samples are collected. Phenylbutyrate metabolite analysis samples will be shipped to the central laboratory.
[k] In the event of a hypersensitivity reaction additional tryptase, complement C3, and ADA samples will be taken at 3 and 24 hours after the start of the reaction. Samples will be shipped to the central laboratory.
[l] In the event of a hyperammonemic episode, additional phenylbutyrate metabolite samples will be taken at the same time as ammonia samples are collected. Samples will be shipped to the central laboratory
[m] Individualized Diet Management questionnaire is a record of protein prescribed and consumed. During the long-term extension period, protein prescribed and consumed will be collected on the 3 days prior to Visits LTE05, LTE09, and then every 12 weeks.
[n] Neuromotor assessments are detailed in Section 8.4. At visits where neuromotor assessments are conducted, the 2MWT will be the first neuromotor assessment performed. Every effort will be made to schedule neuromotor assessments on their assigned weeks; if this is not possible, these assessments may be delayed by up to 2 weeks.
[o] Required neurocognitive assessments by age are detailed in Section 8.8. Required neurocognitive assessments are to be conducted approximately every 6 months during the Follow-Up period beginning from the first blinded dose in this period. Every effort will be made to schedule neurocognitive assessments on their assigned weeks; if this is not possible, these assessments may be delayed by up to 2 weeks.
[p] The final dose telephone follow-up will occur 4 weeks after the subject receives the final dose of pegzilarginase, regardless of when that final dose is administered.

Table 15: Schedule of Assessments – Open-Label LTE144-LTE150/EOS

Blinding	Open-Label (142 weeks)						
Visit ID	LTE144	LTE145	LTE146	LTE147	LTE148	LTE149	EOS LTE150
LTE Study Day	1002	1009	1016	1023	1030	1037	1044
Drug Administration							
Open-label IMP administration	X	X	X	X	X	X	
Clinical Assessments							
Height and weight		X					X
Vital signs (prior to dosing on dosing days)[a]	X	X	X	X	X	X	X
ECG							X
Physical examination[b]							X
Neurological examination[c]							X
Laboratory							
Pregnancy Test[d]		X					X
Prior to dosing arginine and ornithine[e]	X				X		X[f]
Guanidino compounds	X						X
Plasma amino acids and ammonia[g]							X
Hematology, coagulation[h]							X
Blood chemistry[h]							X
Urinalysis[h]							X
Growth hormone and IGF-1							X
Tryptase, complement C3[i]							
ADAs[j]							
Phenylbutyrate Metabolite Analysis[k]							
Hypersensitivity reaction – additional tryptase, complement C3, ADA samples[l]							

**Blinding**	**Open-Label (142 weeks)**						
**Visit ID**	**LTE144**	**LTE145**	**LTE146**	**LTE147**	**LTE148**	**LTE149**	**EOS LTE150**
**LTE Study Day**	**1002**	**1009**	**1016**	**1023**	**1030**	**1037**	**1044**
Hyperammonemic episode – additional phenylbutyrate metabolite samples[m]							
Outcomes							
Individualized Diet Management questionnaire[n]							X
Seizure frequency	X	X	X	X	X	X	X
Adaptive behavior, QoL							X
Neuromotor function assessments[o]							X
Neurocognitive development[p]							X
Safety							
Adverse events	X	X	X	X	X	X	X
Concomitant medications	X	X	X	X	X	X	X
4 weeks after final dose telephone follow-up[q]							X

ADAs = anti-drug antibodies; ECG = electrocardiogram; EOS = End-of-Study Visit; FU = Follow-Up Visit; GC = guanidino compound; IGF-1 = Insulin-like Growth Factor-1; IMP = investigational medicinal product; PE = physical examination; PK = pharmacokinetic(s); QoL = quality of life.
Subjects may continue treatment in the open-label portion of the study until the study drug becomes available through another means or the Investigator, Sponsor or subject determine it is no longer in the subject's interest to receive the study medication. If any subject is participating in the Blinded Follow-up when the data from the Randomized Period are locked and unblinded, the remaining visits for that subject in the Blinded Follow-up can be unblinded.
All assessments on a dosing day are to be obtained prior to dose administration unless otherwise indicated.
NOTE: Study visits will be conducted every 7 days ±1 day during the study.

[a] Vital signs include systolic and diastolic blood pressure, pulse, temperature, and respirations prior to dosing and as clinically indicated.
[b] Brief physical examination to document clinically significant changes from Baseline.
[c] Neurological examination is described in Section 8.3.
[d] Serum pregnancy test will be collected at screening and end of treatment; testing at all other timepoints can be urine or serum tests (females of childbearing potential only)
[e] After LTE24, prior to dosing arginine and ornithine samples are required every 4 weeks but may be performed more frequently as clinically indicated at the discretion of the Investigator.
[f] This sample will be taken to determine arginine and ornithine levels at the EOS visit.
[g] Samples for determination of amino acids and ammonia will be collected prior to dosing. All amino acid and ammonia samples will be drawn after a 4-hour fast when clinically and logistically feasible. The time of the last dose of EAAs must be recorded.
[h] See Section 8.11.

[i] Samples for tryptase and complement C3 will be collected at Baseline. Samples will be shipped to the central laboratory.

[j] Samples for ADAs will be collected at Baseline and pre-dose at regular intervals throughout the study according to the Schedules of Assessments. Samples will be shipped to the central laboratory.

[k] Samples for phenylbutyrate metabolite analysis will be collected at LTE01 and upon the occurrence of any hyperammonemic episode at the same time as ammonia samples are collected. Phenylbutyrate metabolite analysis samples will be shipped to the central laboratory.

[l] In the event of a hypersensitivity reaction additional tryptase, complement C3, and ADA samples will be taken at 3 and 24 hours after the start of the reaction. Samples will be shipped to the central laboratory.

[m] In the event of a hyperammonemic episode, additional phenylbutyrate metabolite samples will be taken at the same time as ammonia samples are collected. Samples will be shipped to the central laboratory

[n] Individualized Diet Management questionnaire is a record of protein prescribed and consumed. During the LTE extension period, protein prescribed and consumed will be collected on the 3 days prior to Visits LTE05, LTE09, and then every 12 weeks.

[o] Neuromotor assessments are detailed in Section 8.4. At visits where neuromotor assessments are conducted, the 2MWT will be the first neuromotor assessment performed. Every effort will be made to schedule neuromotor assessments on their assigned weeks; if this is not possible, these assessments may be delayed by up to 2 weeks.

[p] Required neurocognitive assessments by age are detailed in Section 8.8. Required neurocognitive assessments are to be conducted approximately every 6 months during the Follow-Up period beginning from the first blinded dose in this period. Every effort will be made to schedule neurocognitive assessments on their assigned weeks; if this is not possible, these assessments may be delayed by up to 2 weeks.

[q] The final dose telephone follow-up will occur 4 weeks after the subject receives the final dose of pegzilarginase, regardless of when that final dose is administered.

2. INTRODUCTION

Co-Arg1-PEG (pegzilarginase) is a cobalt substituted, pegylated human recombinant arginase 1 enzyme expressed in *E. coli* that metabolizes arginine. The human arginase 1 enzyme is modified during the manufacturing process with cobalt substitution of the normal manganese metal co-factor, and subsequently pegylated. Pegzilarginase substitutes for the deficient arginase 1 enzyme in subjects with Arginase 1 Deficiency (ARG1-D) by providing an alternate pathway for arginine breakdown in the plasma via the enzymatic conversion of arginine to its natural metabolic product ornithine.

2.1. Study Rationale

Subjects with ARG1-D show persistent hyperargininemia and continued disease progression despite current individualized disease management (IDM) consisting of severe protein restriction and essential amino acid (EAA) supplementation and/or ammonia scavengers (Huemer 2016). The failure to adequately lower plasma arginine levels with IDM into the normal range (40 to 115 µM), or even below the current guideline-recommended level of 200 µM in most cases, is believed to be due to both the practical challenges of adhering to a protein-restricted diet rigorous enough to lower plasma arginine levels and the important contribution of whole-body protein turnover to plasma arginine flux (Häberle 2012, Huemer 2016, Lambert 1991, Oeffinger 2008, Prasad 1997, Wu 1998).

Two lines of evidence support the concept that lowering plasma arginine levels has the potential to slow disease progression in subjects with ARG1-D. First, in subjects exposed to sustained high levels of arginine due to a delayed disease diagnosis, the subsequent lowering of plasma arginine with severe dietary protein restriction has been demonstrated to lead to some modest improvements in disease manifestations (Cederbaum 1979, Cederbaum 1982, Lambert 1991). Second, in a severely affected pediatric case unresponsive to IDM approaches, lowering of plasma arginine using an exchange blood transfusion to enhance extra-hepatic arginase activity resulted in an improvement in clinical status, including a reduction in spasticity (Sakiyama 1984).

Pegzilarginase has been shown in previous studies to produce marked, rapid, and sustained reductions in plasma arginine levels in subjects with ARG1-D, allowing substantially improved arginine control relative to what can be achieved with IDM approaches. The improved control of plasma arginine levels was accompanied by clinical improvements in one or more instruments of neuromotor function or adaptive behavior, consistent with the hypothesis that improved plasma arginine control has the potential to slow disease progression in affected subjects. The goal of the current study is to confirm these preliminary findings in a blinded, placebo-controlled design and to gain further understanding of the impact of pegzilarginase on a broader range of disease manifestations.

2.2. Background

Arginase 1 deficiency (ARG1-D) (Orpha number ORPHA90; ICD-10 code: E72.2; OMIM number 207800) is a rare, progressive, multisystem, autosomal recessive disease (Summar 2013, Diez-Fernandez 2018, Schlune 2015, Waisbren 2018).

This disease typically presents in early childhood and is caused by deficiency in the enzyme arginase 1 (ARG1 [EC 3.5.3.1]), which leads to 2 important harmful metabolic effects:

- Accumulation of high levels of arginine and arginine-derived metabolites.
- Impairment of the urea cycle, which leads to episodic elevation of ammonia levels.

The high plasma arginine level is believed to be the key driver of spasticity, developmental delay, and seizures, which develop in early childhood and progress over time (De Deyn 1997, Waisbren 2018). The lower-limb spasticity in early childhood impairs mobility and balance, leading to difficulties in walking and climbing stairs. School performance and educational achievement is markedly impacted by the developmental delay and the ensuing cognitive decline. The neuromotor and neurocognitive effects, which occur in spite of IDM approaches, result from persistently elevated arginine levels that have a profound impact on daily functioning and quality of life from early in life with progressive worsening over time leading to severe disabilities and early death (De Deyn 1997, Oeffinger 2008, Prasad 1997, Carvalho 2012).

In addition to the severe neuromotor and neurocognitive manifestations, which dominate the clinical picture, subjects with ARG1-D manifest other medically important disease-related abnormalities, including complications due to hyperammonemia, hepatocellular injury, inadequate nutrition, and growth impairment. These abnormalities are a result of the disease or the current disease management with severe protein restriction.

The goals for long-term management of subjects with ARG1-D are to reduce plasma arginine levels without adversely impacting normal growth and development and to prevent hyperammonemia. Recommended disease management involves a common framework of severe protein restriction and EAA supplementation and/or use of ammonia scavengers. In isolation, management of arginine levels is complicated by the impairment of the of urea cycle and the need to ensure an adequate intake of protein for proper growth and development while avoiding hyperammonemia. Disease management is individualized with titration of different components to ensure optimal control of plasma arginine levels, adequate protein intake for proper growth and development, and prevention of hyperammonemia. This common framework is IDM, and consists of severe protein restriction and EAA supplementation and/or the use of ammonia scavengers.

Severe dietary protein restriction has been shown to lower plasma arginine levels in some subjects, with amelioration of some of the disease-related abnormalities, thus providing support for the value of arginine reduction. However, this approach is inadequate in most subjects as demonstrated by the persistence of the marked hyperargininemia. Moreover, the diet is difficult to maintain and manage especially in growing children, and requires supplementation with unpalatable EAA formula to maintain a safe amino acid intake (Häberle 2012, Huemer 2016, Lambert 1991, Burrage 2015). Liver transplantation has been reported to achieve disease normalization in some subjects, but this intervention is available to only a small fraction of subjects and carries substantial additional risks.

The progressive nature of this disease, despite current IDM approaches, highlights the significant unmet need for a therapy that will lower arginine levels beyond those achievable with current standard disease management and thus provide the potential to slow or halt the progression of neuromotor and/or neurocognitive deterioration in these subjects.

Pegzilarginase is being investigated as an enzyme therapy for ARG1-D. Biological activity of pegzilarginase has been demonstrated based on nonclinical studies and clinical data from the CAEB1102-101A study (Study 101A) and CAEB1102-102A study (Study 102A) in subjects with ARG1-D. The current study seeks to confirm the clinical utility of pegzilarginase for the treatment of patients with ARG1-D.

The Investigator's Brochure (IB) provides a detailed description of the chemistry (IB Section 3), nonclinical data (IB Section 4), clinical pharmacokinetics (PK) and pharmacodynamics (PD) (IB Section 5.1), immunogenicity (IB Section 5.2), clinical safety (IB Section 5.3), and activity (IB Section 8.9) of pegzilarginase.

2.3. Benefit-Risk Assessment

As discussed in Section 2.2, ARG1-D is a rare, serious, progressive disease with significant unmet need despite IDM approaches. The disease typically presents in childhood with serious neuromotor and neurocognitive manifestations that progress with increasing age with resultant severe disabilities, significant ill health, and a shortened life expectancy.

Nonclinical studies of pegzilarginase at pharmacological doses, and at doses substantially more than those proposed in this study, revealed no significant risks. Subsequent clinical experience with pegzilarginase in both adult and pediatric subjects with ARG1-D, including 1 completed Phase 1/2 study (Study 101A) and 1 ongoing long-term extension (LTE) study (Study 102A), indicates that pegzilarginase has a tolerability profile that supports further development at intravenously (IV) administered doses ranging from 0.015 mg/kg to 0.2 mg/kg. Treatment-related adverse events (TEAEs) in subjects with ARG1-D have generally been mild.

Data from the completed Phase 1/2 study (Study 101A) and available data from the ongoing Phase 1/2 LTE study (Study 102A) have shown that pegzilarginase produces marked and sustained reduction of plasma arginine levels in subjects with ARG1-D. The improved control of plasma arginine levels was accompanied by clinically relevant treatment effects in neuromotor function and adaptive behavior.

Adverse events (AEs) associated with administration of approved enzyme replacement therapies (ERTs) include hypersensitivity reactions. Reactions requiring intervention are typically managed by temporarily interrupting or discontinuing infusion and administering antihistamines and/or antipyretics. Hypersensitivity reactions are possible with pegzilarginase and have been manageable with standard medical practice.

Given the potential for hypersensitivity reactions with ERT administration, measures have been incorporated into this protocol to minimize risk while monitoring subject safety.

Overall, the benefit-risk assessment for pegzilarginase is considered favorable, given the progressive nature of the disease despite IDM, the manageable risk profile associated with pegzilarginase treatment, the ability of pegzilarginase to significantly lower plasma arginine levels over a short time period, and the clinical improvements in neuromotor and adaptive behavior manifestations observed with pegzilarginase treatment in subjects with ARG1-D.

More detailed information about the known and expected benefits and risks and expected or observed AEs associated with pegzilarginase treatment may be found in the IB.

3. OBJECTIVES AND ENDPOINTS

Objectives of the study, along with the endpoints used to meet those objectives, are described in Table 16. Additional details on endpoint definitions are provided in Section 10.

Table 16: Objectives and Endpoints

Objectives	Endpoints
PRIMARY	
• To demonstrate the efficacy of pegzilarginase relative to placebo based on a statistically significant decrease in plasma arginine concentrations	• Change from Baseline in plasma arginine after 24 weeks of study treatment
KEY SECONDARY	
• To demonstrate the efficacy of pegzilarginase relative to placebo based on key mobility outcome measures	• Mean change from Baseline at Week 24 in the 2-Minute Walk Test (2MWT)<br>• Mean change from Baseline at Week 24 in the Gross Motor Function Measure-88 (GMFM) Part E
SECONDARY	
To further demonstrate the clinical and biochemical efficacy of pegzilarginase relative to placebo	
• To compare pegzilarginase with placebo with respect to the proportion of subjects whose endpoint arginine value falls below target guidance of 200 µM	• A subject is considered a responder if their endpoint arginine value is <200 µM after 24 weeks of study treatment, and a non-responder otherwise
• To compare pegzilarginase with placebo with respect to the proportions of subjects whose endpoint arginine value falls within the normal range of 40 to 115 µM	• A subject is considered a responder if their endpoint arginine value is in the normal range (≥40 µM to ≤115 µM) after 24 weeks of study treatment, and a non-responder otherwise
• To compare pegzilarginase with placebo for changes in ornithine and guanidino compounds (GCs)	• Change from Baseline in ornithine and GCs after 24 weeks of study treatment
• To compare pegzilarginase with placebo with respect to other aspects of mobility	• Mean change from Baseline at Week 24 in the GMFM Part D<br>• Mean change from Baseline at Week 24 in Functional Mobility Scale (FMS) and Gillette Functional Assessment Questionnaire (GFAQ)
• To compare pegzilarginase with placebo with respect to adaptive behavior	• Mean change from Baseline at Week 24 in Vineland Adaptive Behavior Scales (VABS)-II
• To evaluate the safety and immunogenicity of pegzilarginase	• Adverse events (AEs) and anti-drug antibodies (ADAs) will be collected
• To further characterize the pharmacokinetic (PK) profile of pegzilarginase	• PK and PD data will be analyzed

Objectives	Endpoints
TERTIARY	
• To compare the proportions of subjects with response in composite clinical outcome, on pegzilarginase and placebo	• Clinical response is defined as a subject exhibiting a mobility response in the 2MWT or GMFM Part D or GMFM Part E as defined in Section 10.3.1
• To compare response on pegzilarginase with placebo with respect to other aspects of mobility	• Response for FMS and GFAQ is defined as a 1-level change at any distance and a 2-level change at any distance, respectively
• To compare response on pegzilarginase with placebo with respect to adaptive behavior	• Response for VABS-II is defined as an improvement by ≥7.5 points
• To compare pegzilarginase with placebo with respect to improvement of spasticity and objective measures of neurological/neuromotor manifestations	• Change from Baseline for functional and spasticity measurements by Modified Ashworth Scale (MAS)
• To further describe caregiver and clinician global impression of the impact of pegzilarginase on motor function and/or adaptive behavior	• Caregiver/Clinician Global Impression of Change, Global Impression of Severity
• To describe the impact of pegzilarginase on fine motor function	• 9-Hole Pegboard
• To describe the impact of pegzilarginase on quality of life (QoL)	• Pediatric Quality of Life (PedsQL)<br>• 36-Item Short Form Health Survey (SF-36)<br>• Zarit Burden Interview (12-item) (ZBI-12)
• To describe the impact of pegzilarginase on neurocognition and memory	• Neurocognitive endpoints:<br>– Bayley Scales of Infant Development (BSID)-III (as age appropriate)<br>– Wechsler Intelligence Batteries
• To describe the diets maintained by subjects in the study	• 3-day diet records will be collected
• To describe the effect of pegzilarginase on plasma ammonia	• Plasma ammonia data will be collected
• To evaluate the effects of pegzilarginase on growth in pediatric subjects	• Z-scores for height, weight, and body mass index (BMI) will be computed using the Centers for Disease Control and Prevention (CDC) growth curves for subjects younger than 18 years of age
• To characterize the long-term (>24 to approximately 178 weeks) treatment effects of pegzilarginase administration	• Endpoints from open-label extension period data will be used, in particular to evaluate the extent to which efficacy is sustained over the period from Week 24 to Week 48

4. STUDY DESIGN

4.1. Overall Design

CAEB1102-300A is a multi-center, randomized, double-blind, placebo-controlled study to evaluate the safety and efficacy of pegzilarginase in subjects with ARG1-D. This study will consist of:

1. A screening period of 3 to 4 weeks duration to collect all necessary information to ensure the subjects meet study eligibility criteria and to establish Baseline plasma arginine data, collect prescribed diet data, and determine adherence to prescribed diet using a diet diary.
2. A randomized, double-blind treatment period of 24 weeks.
3. An open-label LTE period of up to approximately 150 weeks in which all subjects receive active pegzilarginase. The first 8 weeks of treatment will remain blinded to ensure that study data relating to the randomized period is collected prior to unblinding.

Subjects will be randomized to treatment following completion of all screening assessments and confirmation of study eligibility in a 2:1 ratio to receive weekly IV infusions of pegzilarginase plus IDM or placebo plus IDM during the 24-week double blind treatment period. After completion of the 24-week double-blind treatment period, each subject will enter the LTE period, the first 8 weeks of which are blinded (Section 6.6). After the first 8 weeks of the blinded LTE period, subjects will have the option to receive pegzilarginase by SC administration, with Investigator and Sponsor approval. The first 4 SC doses will be given at the investigational site. The initial mg/kg SC dose may be the same as the IV dose. Subsequent SC doses may be administered outside of the investigational site by appropriately trained home healthcare personnel if considered safe and appropriate in the opinion of the Investigator in consultation with the Sponsor. During the LTE period, all subjects will receive pegzilarginase plus IDM.

Following completion of the assessments in this study, subjects may continue to receive pegzilarginase through other means.

4.2. Impact of Coronavirus Disease (COVID-19)

The COVID-19 pandemic has impacted clinical research. The safety of study participants is paramount and must be at the heart of every decision, regardless of any potential consequences for an ongoing study. Consequently, various regulatory authorities have issued guidance on how to manage the impact of this pandemic on the methodological aspects of ongoing research (FDA September 2020, EMA April 2020, EMA June 2020).

There is an ethical mandate to proceed with a study that has been started so that the efforts taken by study participants and physicians can benefit drug development and inform patient care. Guidance strongly recommends that Sponsors integrate all available knowledge from the ethical, medical, and methodological perspective into decision making about the future conduct of a study while carefully considering advice from regulatory and healthcare authorities. General advice on how the different aspects related to the COVID-19 pandemic should be handled is not yet feasible, as the implications on clinical studies are expected to be manifold. COVID-19's impact on recruitment, data collection, and analysis and interpretation of results for each study needs a thorough case-by-case assessment.

Due to the evolving nature of the COVID-19 pandemic and the variation in guidance and restrictions across countries and sites, an amendment for specific anticipated deviations is not feasible. Investigators as well as study sites and regulatory bodies such as IRBs/ECs are encouraged to adapt study procedures to ensure subject safety and compliance with local and regulatory procedures within the context of this protocol. Any variance from this study's assessments and procedures will be clearly documented and captured as protocol deviations and annotated as a result of the COVID-19 pandemic.

4.3. Scientific Rationale for Study Design

4.3.1. Use of Placebo

A placebo-controlled study design is employed to demonstrate the efficacy of pegzilarginase in subjects with ARG1-D. It is well recognized that improvements in disease parameters can occur in subjects not receiving active treatment due to changes in behavior and other factors in response to clinical study participation. Current treatment for ARG1-D subjects involves severe protein restriction and EAA supplementation and/or the use of ammonia scavengers—collectively referred to as IDM. Given the observation from literature case reports that tightly monitored and controlled IDM can result in modest reduction in arginine levels and modest clinical improvements (Cederbaum 1979, Cederbaum 1982, Lambert 1991, Prasad 1997), the use of a placebo arm assists in the interpretation of any observed effects on plasma arginine and other efficacy endpoints that have the potential to be impacted by improved compliance with any of these IDM measures.

In addition, the use of a placebo arm assists in the interpretation of secondary endpoints and safety endpoints, which may have the potential to be influenced by subject or Investigator knowledge of assigned study drug.

Use of placebo is considered ethical in this study for several reasons: (1) all subjects will continue to receive IDM for ARG1-D while participating in this study (2) available clinical literature and other information suggest that although ARG1-D is a progressive disease, significant progression of the disease is unlikely over the period of 24 weeks; and (3) the study will exclude subjects with other significant comorbidities that may put them at undue risk during the placebo-controlled phase of the study.

4.3.2. Study Population

Although ARG1-D typically presents in childhood, the literature describes heterogeneity in onset and progression across the age continuum (Jain-Ghai 2011, Oeffinger 2008, Prasad 1997, Carvalho 2012, Cowley 1998). Therefore, it is important to assess safety and efficacy of pegzilarginase in both pediatric and adult subjects.

As noted in literature case reports and Aeglea's Phase 1/2 study in adult and pediatric subjects, reduction of plasma arginine levels results in clinical improvements, suggesting that hyperargininemia is neurotoxic (Cederbaum 1982, De Deyn 1997). Early intervention in pediatric patients, prior to the development of significant neuromotor, adaptive behavior and neurocognitive manifestations, offers the potential to halt the progression or facilitate clinical improvement, therefore inclusion of pediatric and adult patients is justified.

It is well recognized that early therapeutic intervention can delay onset and/or slow the progression of many diseases leading to the prevention of irreversible disease-related complications and better clinical outcomes (Desnick 2012). Given the importance of demonstrating clinically relevant treatment effects in addition to reductions in plasma arginine levels in this clinical trial, enrollment for this study will be limited to subjects with a measurable deficit in at least 1 of the ARG1-D manifestation(s) considered for the key secondary/other secondary endpoints: 2MWT or GMFM-D or GMFM-E.

Baseline deficits for the key secondary/other secondary endpoints are described in Table 17.

Table 17: Definition of Baseline Deficits for Key Secondary/Other Secondary Endpoints

Domain	Assessment	Component	Definition of Baseline Deficit		
Mobility	Timed Walk Test	2MWD* (meters)	Definition of Baseline deficit for 2MWT varies by age and sex.		
			Age	Female	Male
			3-5	<112.9	<110.6
			6-8	<155.8	<154.9
			9-11	<172.0	<169.9
			12-15	<168.7	<172.1
			16-17	<167.5	<173.4
			≥18	<142.4	<148.8
	GMFM[†]	Part D	<35		
		Part E	<68		

2MWT = 2-Minute Walk Test; 2MWD = 2-Minute Walk Distance; GMFM = Gross Motor Function Measure
* Definition of Baseline deficit is calculated from the NIH Toolbox motor domain dataset (2-minute Walk Endurance Test).
† Definition of Baseline deficit is from Oeffinger 2008.

In Study 101A, all 16 subjects in the study had Baseline GMFM function at the ceiling of the test for Parts A and B. Nine subjects had Baseline scores at the ceiling of the test for Part C, with 7 other subjects' scores very close to the ceiling. For this reason, Parts D and E were considered the most appropriate measures likely to be sensitive to change in this study population.

The study is designed to assess the effect of pegzilarginase in combination with the subject's IDM, which typically includes a prescribed diet with severe protein restriction and EAA supplementation and/or the use of ammonia scavengers. Subjects will have a stable IDM plan as demonstrated during the screening period prior to study participation, including the amount of prescribed protein and the amount of prescribed EAAs and/or the use of a prescribed dose of ammonia scavenger medication if applicable. Subjects must be willing and able to maintain consistent dietary protein intake during the double-blind portion of the study. Subjects and/or their caregivers will be counseled by the Investigator and dietician not to modify their IDM during the double-blind portion of the study unless there is a clear medical reason to do so. Compliance with IDM will be captured in the electronic case report form (eCRF). Subjects will be required to have a stable, consistent diet for the entire duration of the blinded period, which includes the 24-week randomization period and the first 8 weeks of the open-label period of the study. A consistent diet is defined as one in which the prescribed natural protein medical food EAA supplementation and/or the use of ammonia scavengers, and caloric intake does not change more than 15% from Baseline. Any prescribed changes more than 15% from Baseline will be documented.

Plasma arginine was selected as the primary endpoint because: (1) it is mechanistically related to the primary disorder; (2) it is the single common manifestation in all subjects; (3) it is believed to be the key driver of clinical manifestations; and (4) it can be objectively measured utilizing a validated bioanalytical assay.

The clinical impact of a reduction in arginine will be assessed primarily via the key secondary endpoint of mobility as defined in Section 10.3.1.2.

4.4. Measures to Minimize Bias: Randomization and Blinding

4.4.1. Description of Blinding Levels and Access

Central randomization (2:1 ratio, pegzilarginase:placebo) will be used to minimize bias. The computer-generated randomization schedule will be created and managed by a Sponsor designee. Randomization will be accomplished through IXRS.

Randomization will be stratified by severity of prior history of hyperammonemia as outlined in the IXRS manual. The purpose of this stratification is to minimize potential bias from a treatment-group imbalance in this important factor (see Section 10.3.4).

Specific details regarding personnel and level of data access are provided in Table 18.

All site personnel involved with the study, including subjects, families, caregivers, Investigators, and expert assessors of relevant endpoints, and all Sponsor and contract personnel except as noted in Table 18, will be blinded to the subject's randomized treatment assignment to minimize potential biases in assessment of safety and clinical outcomes.

Each site will have an unblinded pharmacist, and an unblinded physician where required, to manage protocol-defined dose adjustments. An unblinded pharmacist, and where required, physician, will be present at each site to manage any required changes in study drug dosage. The unblinded pharmacist and/or physician will receive instructions to adjust pegzilarginase dose levels based on the dosing algorithm (Section 6.5) from the IXRS. Unblinded site personnel must not disclose treatment allocation, study results, or other study-related information to blinded persons involved with the study.

An unblinded clinical research associate (CRA) will conduct drug accountability at the site. Specific personnel will be designated to manage the electronic randomization system and therefore will have access to subject treatment assignments.

Laboratory results for arginine and ornithine have the potential to allow the Investigator to infer the subject's treatment group. Therefore, results for these laboratory tests will not be provided to the Investigator or other blinded individuals, including subjects, families, Sponsor personnel, or assessors, for visits during blinded treatment (Table 18). Sites will also be instructed not to request arginine and/or ornithine assessments outside the study protocol. Note that although the randomized double-blind treatment period ends at Week 24, these laboratory results will continue to be blinded until all subjects have completed the blinded portion of the study to LTE01 and the database to this timepoint has been frozen and formal unblinding of the 24-week DB period has occurred.

4.4.2. Breaking the Blind

If required for subject safety, an Investigator may break the blind for an individual subject on an emergency basis. The blind for an individual subject will only be broken under exceptional circumstances, in the rare event of a medical emergency in which knowledge of the investigational product administered to that subject is essential for medical management of the subject. If feasible, the Sponsor must be consulted before breaking the blind and will in all cases be informed in a timely manner if the blind is broken. Full details on the method for emergency unblinding are provided in the IXRS manual.

If a subject's treatment assignment is formally unblinded (ie, if the subject's formal treatment assignment is provided to the Investigator or subject) for any reason, that subject must be discontinued from the double-blind treatment period. At the discretion of the Investigator the subject may transition to the LTE period (if eligible).

Table 18: Study Personnel and Levels of Data Access and/or Unblinding

Level of Access		
**Data Access**	**Title/Role**	**Affiliation**
**Study-Level Randomization**	Contract Unblinded Statistician for SRC Outputs	SDC
	Contract Unblinded SAS Programmer for SRC Outputs	SDC
	Contract Randomization Service	Cenduit
	Contract Safety Group	Synteract
**Site-Level Randomization**	Blinded Physician (Investigator)	For each site
	Blinded Study Coordinator	For each site
	Unblinded Pharmacist	For each site
	Unblinded Physician	As required by site
	Unblinded Contract CRA	IQVIA, for drug accountability
**Subject-Level Randomization**	SRC	Independent
**eCRF Subject Data Access**	Contract CRAs	IQVIA
	Contract Data Management	IQVIA
	Contract Blinded Statistical Team	SDC
	Contract Coder	IQVIA
	Contract Study Management	IQVIA
	Contract Exit Interview and Psychometric Analyses	COS
	Sponsor Biostatistician	Aeglea
	Sponsor SAS Programmers	Aeglea
	Sponsor Data Management	Aeglea
	Sponsor Medical Monitor	Aeglea
	Sponsor Backup Medical Monitor	Aeglea
	Sponsor Safety Physician	Aeglea

Level of Access		
**Data Access**	**Title/Role**	**Affiliation**
	Sponsor Clinical Scientist	Aeglea
	Sponsor Quality Assurance Lead	Aeglea
**Laboratory Data: Arginine, ornithine, and GC levels only**	Charles River Laboratory (Contract Laboratory)	Charles River Laboratory, SRC members, Unblinded team at SDC
ADA Data	BioAgilytix (Contract Laboratory)	BioAgilytix
PK Data	NuVentra (Contract Laboratory), Contract Unblinded Programmer, Contract Unblinded Statistician	SDC, SRC
**Blinded**	Subjects and their families, friends, and caregivers<br>Investigators, study coordinators, and other investigative site staff not specifically noted as having access to data<br>Expert assessors of study endpoints<br>Sponsor personnel and others contracted by the Sponsor to perform study-related duties, except as noted in this table.	

4.5. Justification for Dose

For subjects randomized to pegzilarginase, the weekly IV dose of 0.10 mg/kg was selected based on available safety, PK, PD, and clinical results from Study 101A and Study 102A, which indicate that this dose was well tolerated, and is anticipated to provide a desirable level of plasma arginine control in the majority of the target population.

The final dose in Study 101A was between 0.04 and 0.20 µM for the 14 subjects who had completed at the time of database lock (23 May 2019). The median final dose was 0.09 mg/kg; thus, the starting dose of 0.10 mg/kg was selected as being representative of an effective and well-tolerated dose.

Dose modifications are discussed in Section 6.5.

4.6. Definitions of End of Randomized Period and End of Study

4.6.1. End of Randomized Period

The End of Randomized Period (EORP) for an individual subject is defined as the date on which the subject completes his/her last assessment for the EORP Visit. The EORP for the study is defined as the date that the last subject completes his/her last assessments for the EORP Visit.

4.6.2. End of Study

The End of Study (EOS) for an individual subject is defined as the date on which the subject completes his/her last assessment for the study. The EOS overall is defined as the date that the last study assessment is completed. Following completion of the assessments, subjects may continue to receive pegzilarginase through other means.

5. STUDY POPULATION

5.1. Inclusion Criteria

Subjects are eligible to be included in the study only if all the following criteria apply:

1. The subject and/or parent/guardian provides written informed consent/assent, which includes compliance with the requirements and restrictions listed in the informed consent form (ICF) and in this protocol.
2. A current diagnosis of ARG1-D as documented in medical records, which must include 1 of the following: elevated plasma arginine levels, a mutation analysis that results in a pathogenic variant, or reduced RBC arginase activity. For entry into this study, subjects must also fulfill the following plasma arginine criteria:
   a. The average of all measured values of plasma arginine during the screening period prior to the randomization visit (Visit 1, Study Day 1) is ≥ 250 µM.
   b. If a subject is re-screened (Section 5.3), the only values that are considered for eligibility assessment are those in the current screening period.
3. Subjects must be ≥2 years of age on the date of informed consent / assent.
4. The subject must be assessable for clinically meaningful within-subject change (clinical response) on at least 1 component of 1 assessment included in the key secondary/other secondary endpoints. To be considered assessable, the subject must be able to complete the assessment, and must have a Baseline deficit in at least 1 component as defined in Table 17.
5. Have received documented confirmation from the Investigator and/or dietician that the subject can maintain their diet in accordance with dietary information presented in the protocol, ie, can maintain the current level of protein consumption, including natural protein and EAA supplementation.
6. Subjects receiving ammonia scavenger therapy, anti-epileptic drugs, and/or medications for spasticity (eg, baclofen) must be on a stable dose of the medication for at least 4 weeks prior to randomization and be willing to remain on a stable dose during the double-blind portion and blinded follow-up portions of the study.

7. Female and male subjects may participate. Female subjects of childbearing potential must have a negative serum pregnancy test during the screening period before receiving the first dose of study treatment, and a negative urine pregnancy test on the day of the first dose, prior to the first dose. If the subject (male or female) is engaging in sexual activity that could lead to pregnancy, must be surgically sterile, postmenopausal (no menses for 12 months without an alternative medical cause or a high FSH level in the postmenopausal range in women not using hormonal contraception or hormonal replacement therapy), or must agree to use a highly effective method of birth control during the study and for a minimum of 30 days after the last study drug administration. Highly effective methods of contraception include: combined (estrogen and progestogen containing) hormonal contraception associated with inhibition of ovulation; progesterone-only hormonal contraception associated with inhibition of ovulation; intrauterine device (IUD); intrauterine hormone-releasing system (IUS); or abstinence (refraining from heterosexual intercourse during the entire period of risk associated with study treatment).

5.2. Exclusion Criteria

Participants are excluded from the study if any of the following criteria apply:

1. Hyperammonemic episode (defined as an event in which a subject has an ammonia level ≥100 µM with one or more symptoms related to hyperammonemia requiring hospitalization or emergency room management) within the 6 weeks before the first dose of study drug is administered.
2. Active infection requiring anti-infective therapy within 3 weeks prior to first dose.
3. Known active infection with human immunodeficiency virus (HIV), hepatitis B, or hepatitis C.
4. Extreme mobility deficit, defined as either the inability to be assessed on the GFAQ or a score of 1 on the GFAQ.
5. Other medical conditions or comorbidities that, in the opinion of the Investigator would interfere with study compliance or data interpretation (eg, severe intellectual disability precluding required study assessments).
6. Has participated in a previous interventional study with pegzilarginase.
7. Has a history of hypersensitivity to polyethylene glycol (PEG) that, in the judgment of the Investigator, puts the subject at unacceptable risk for adverse events.
8. Subject is being treated with botulinum toxin-containing regimens or plans to initiate such regimens during the double-blind or blinded follow-up portions of the study or received surgical or botulinum-toxin treatment for spasticity-related complications within the 16 weeks prior to the first dose of study treatment in this study.
9. Is currently participating in another therapeutic clinical trial or has received any investigational agent within 30 days (or 5 half-lives whichever is longer) prior to the first dose of study treatment in this study.
10. Previous liver or hematopoietic transplant procedure.

5.3. Screen Failures and Re-Screening

Screen failure is defined as a subject who consented to participate in the clinical study but was not subsequently randomized. Information to be collected about screen-failed subjects is presented in Table 19.

Subjects who do not meet the eligibility criteria (screen failure) may be re-screened a maximum of 2 times.

Table 19: Information to Be Collected for Subjects Who Are Screen Failures

Category	Information to Be Collected
Demography	Date of birth*, sex, race, ethnicity
Reason for Screen Failure	Specific entry criteria that subject does not satisfy (as many as have been assessed)
ARG1-D Diagnostic History	Date* and method (newborn screening, clinical symptoms, family history) of diagnosis, date* of onset of first symptoms, mutation (if available), results of assessment for arginase activity in red blood cells (RBCs) if available
ARG1-D Medical History	History of each of the following symptoms, including date* of onset (if known): Hyperargininemia, spasticity, seizures, hyperammonemia, developmental delay, impaired growth, impaired motor function/gait, elevated ALT and/or AST, protein-restricted diet, EAA supplementation, use of ammonia scavengers.
ARG1-D Family History	Genetic status (presence/absence of mutation data) of parents (if available), number of affected siblings, age at diagnosis for affected siblings.

* Ages may be substituted for dates, or partial dates may be used, to meet privacy requirements in various jurisdictions.

6. STUDY MEDICATION

6.1. Study Medications Administered

Table 20: Study Medications Administered

Intervention Name	Pegzilarginase	Placebo
**Unit Dose Strength(s) IV** (may also apply to SC depending on weight and dose)	1 mg/mL solution	NA
**Unit Dose Strength(s) SC**	5 mg/mL	NA (subcutaneous [SC]dosing not applicable to blinded, randomized study activities)
**Description**	Pegzilarginase is a cobalt-substituted, pegylated, recombinant human Arginase 1. AEB1102 drug product supplied as a liquid formulation in 10 mL single-use glass vials containing 5 mL of formulated drug product at a concentration of 1 mg/mL or 5 mL of formulated drug product at 5 mg/mL. Pegzilarginase is formulated in 50 mM NaCl, 1 mM K2HPO4, 4 mM KH2PO4, and 1.5% w/v glycerol.	The placebo will consist of the vehicle contained in 10 mL glass vials.
**Premedication**	Premedication with a non-sedating antihistamine is recommended 30 minutes prior to the start of dosing.	Premedication with a non-sedating antihistamine is recommended 30 minutes prior to the start of dosing.
**Dose**	Starting dose 0.10 mg/kg; dose adjustment allowed based on pharmacodynamic response (Section 6.5)	Volume-adjusted placebo infusion to match volume of hypothetical pegzilarginase infusion
**Routes of Administration**	IV Infusion<br>(approximately 30 minutes)<br>SC injection for long-term extension study period activities only	IV Infusion<br>(approximately 30 minutes)<br>SC injection for long-term extension period activities only
**Sourcing**	Provided by Sponsor	Provided by Sponsor
**Packaging and Labeling**	Study medication will be provided in a 10 mL single-use glass vial containing 5 mL of drug product. Each carton of vials will be labeled as required per country requirement.	Placebo will be provided in a 10 mL single-use glass vial containing 5 mL of vehicle. Each carton of vials will be labeled as required per country requirement.
**Storage Conditions**	Filled vials of pegzilarginase must be stored at either ≤-60°C for 36 months, or 2-8°C for 24 months. Drug temperature must be maintained in accordance with the drug product investigational labeling.	Filled vials of placebo must be stored at either ≤-60°C for 36 months, or 2-8°C for 24 months. Drug temperature must be maintained in accordance with the drug product investigational labeling.
**Other Names**	AEB1102<br>Co-Arg1-PEG	NA

NA=not applicable

6.2. Preparation/Handling/Storage/Accountability

The storage conditions for pegzilarginase drug product are either ≤-60°C for 36 months, or 2-8°C for 24 months. Drug temperature must be maintained in accordance with the drug product investigational labeling.

Details for pegzilarginase preparation, handling, storage, and accountability are provided in the Pharmacy Manual. Some specific instructions include:

- The unblinded pharmacist or designee must confirm appropriate temperature conditions have been maintained during transit for all study treatment received and any discrepancies must be reported and resolved before use of the study treatment.
- Only subjects consented and randomized in the study may receive study treatment and only authorized site staff may supply or administer study treatment. All study treatment must be stored in a secure, environmentally controlled, and monitored (manual or automated) area in accordance with the labeled storage conditions with access limited to authorized unblinded site staff.
- The unblinded pharmacist or designee is responsible for study treatment accountability, reconciliation, and record maintenance (ie, receipt, reconciliation, and final disposition records). This responsibility also extends to the home healthcare nurse(s) who will complete drug accountability forms for each dose administered at a subject's home and will provide these records to the unblinded pharmacist or designee at the site.
- A vial of pegzilarginase or placebo will be thawed at room temperature for approximately 60 minutes. IMP must be completely thawed (no ice particles remaining) prior to dose preparation. The thaw and dose preparation must be completed within 120 minutes. Refer to the Pharmacy Manual for complete preparation and administration instructions.
- A central venous access port may be considered for subjects with poor IV access, at the Investigator's discretion.
- The initial calculation of total dose in milligrams of study drug will be based upon the subject's weight at Baseline**.** If the weight changes by >25%, the future doses will be calculated from the revised weight. Consult the Pharmacy Manual for additional details.
- Missed doses will not be made up.
- Further guidance and information for the final disposition of unused study treatment is provided in the Pharmacy Manual.

6.3. Study Medication Compliance

As part of the monitoring procedures for this study, an unblinded CRA will perform on-site drug accountability. Details are provided in the study monitoring plan.

If a subject receives an incorrect dose, then the administered dose will be recorded in the IXRS and the pharmacy records. The subject will then return to the prescribed dose per the protocol algorithm at the next visit.

6.4. Concomitant Therapy

Any medical intervention, including over-the-counter or prescription medicines, vitamins, dietary supplements, vaccines, physical/occupational therapy, medical devices, or surgery that the subject is receiving at the time of informed consent/assent or receives during the study must be recorded along with:

- Reason for intervention
- Dates of administration including start and end dates
- Dosage information including dose and frequency

Additional details, such as route of administration and time of dose, will be collected for hypersensitivity reactions.

It is recommended that subjects not undergo surgical procedures (eg, tendon release) for correction of disease-related abnormalities during the double-blind period. If such procedures are deemed medically necessary, they will be captured in the eCRF. Subjects who are already participating in physical therapy during the screening or Baseline periods will continue with the same therapy during the double-blind period without modification. Subjects who are not participating in physical therapy during screening/Baseline will not initiate physical therapy during the double-blind period unless required for subject safety.

The use of botulinum toxin (BT) is prohibited until subjects have completed after the end of the blinded period (LTE09). Should it be necessary for the subject to utilize BT during the LTE period, it should be discussed with the Sponsor prior to administration. Use of BT should remain stable until the end of the study.

6.5. Dose Modification

Dose modifications will be made based on plasma arginine levels. In general, the dose will be modified if the subject's plasma arginine level – assessed 168 hours after a given dose but prior to the next dose – is outside the range of 50 to 150 µM.

Subjects assigned to pegzilarginase begin at Dose Level 2 (Table 21), 0.10 mg/kg. Beginning with Visit 5 and ending with Visit LTE24, dose modifications, if required, based on plasma arginine values will be implemented by the unblinded pharmacist and/or physician, according to the following algorithm that will be implemented in the IXRS:

- If the plasma arginine level is >150 µM, a single 168-hour sample will be used to increase the dose by 2 dose levels (not to exceed 0.20 mg/kg) if the 2 doses prior to this sample were a) the same dose level in mg/kg, and b) consecutive (with no missed doses).
- If the plasma arginine levels from *2* sequential 168-hour samples (regardless of missed doses) are both <50 µM, the dose is decreased by 1 dose level (Table 21), not to decrease below 0.05 mg/kg.

After Visit LTE 24, the IXRS will no longer be used. Any dose modifications required as a result of plasma arginine levels and/or changes in the subjects' clinical presentation will be implemented by the Investigator in discussion with the Aeglea medical team.

Full details are provided in the Pharmacy Manual.

Table 21: Dose Adjustments for Pegzilarginase

Dose Level[a]	Dose
1 (Minimum Possible Dose)	0.05 mg/kg
2 (Starting Dose)	0.10 mg/kg
3	0.15 mg/kg
4 (Maximum Possible Dose)	0.20 mg/kg

[a] Pegzilarginase dosing starts at level 2, 0.10 mg/kg. Dose increases, when required, are by 2 dose levels. Dose decreases are by 1 dose level.

6.6. Treatment Options

Upon completion of the 24-week double-blind treatment period of the study, all eligible subjects will begin an 8-week blinded follow-up treatment period during which all subjects will receive pegzilarginase.

Subjects initially randomized to pegzilarginase will receive the optimized dose they received during the 24-week double-blind treatment period (which may be adjusted as clinically indicated during this period per Section 6.5). Subjects initially randomized to placebo during the 24-week double-blind treatment period will begin the 8-week blinded treatment period at a dose of 0.10 mg/kg that may be adjusted during the LTE period based on arginine levels (Section 6.5). All subjects will have their final double-blind assessments (ie, Week 24 assessments) prior to initiation of the LTE period.

After the first 8 weeks of the blinded LTE period, subjects will have the option to receive pegzilarginase by SC administration, with Investigator and Sponsor approval. The first 4 SC doses will be given at the investigational site. The initial mg/kg SC dose may be the same as the IV dose. Subsequent SC doses may be administered outside of the investigational site by appropriately trained home health care personnel if considered safe and appropriate in the opinion of the Investigator in consultation with the Sponsor. During the LTE, all subjects will receive pegzilarginase plus IDM.

Subjects may continue treatment in the open-label portion of the study until the study drug becomes available through another means or the Investigator, Sponsor, or subject determine it is no longer in the subject's interest in continuing to receive study medication.

7. DISCONTINUATION

7.1. Discontinuation of Study Medication

A subject may withdraw from the study and study treatment at any time at his/her own request or may be withdrawn at any time at the discretion of the Investigator or Sponsor for any reason. Specific reasons for discontinuation may include, but are not restricted to, the following:

- Intercurrent illness
- Pregnancy
- Medically significant AEs, including AEs requiring emergency unblinding
- Significant protocol deviation or non-compliance
- Termination of the study by the Sponsor

Subjects that discontinue any portion of the study for life-threatening AEs assessed as related to pegzilarginase may not enter subsequent dosing periods of the study.

Subjects who discontinue study treatment for any reason during the 24-week double-blind treatment period of the study prior to completing all required study visits will undergo all evaluations and procedures specified at the EORP Visit in the Schedule of Assessments (Table 2). Subjects that discontinue early from the blinded or LTE periods of the study will undergo the procedures and evaluations specified for the End of Open-Label Visit as specified in the Schedule of Assessments (Table 15). All subjects will be contacted approximately 4 weeks after their last visit for follow up of AEs.

If the subject withdraws consent for disclosure of future information, the Sponsor may retain and continue to use any data collected before such withdrawal of consent.

7.2. Lost to Follow Up

A subject will be considered lost to follow up if he or she repeatedly fails to return for scheduled visits and is unable to be contacted by the study site.

The following actions must be taken if a subject fails to return to the clinic for a required study visit:

1. The site must attempt to contact the subject and reschedule the missed visit as soon as possible, counsel the subject on the importance of maintaining the assigned visit schedule, and ascertain if the subject wishes to and/or will continue in the study.
2. Before a subject is deemed lost to follow up, the Investigator or designee must make every effort to regain contact with the subject (where possible, 3 telephone calls and, if necessary, a certified letter to the subject's last known mailing address or local equivalent methods). These contact attempts will be documented in the subject's medical record.
3. Should the subject continue to be unreachable, he/she will be considered to have withdrawn from the study.

8. STUDY ASSESSMENTS AND PROCEDURES

Study procedures will be performed according to the Schedules of Assessments (Table 1 through Table 15). At visits where neuromotor assessments are conducted, the 2MWT will be the first neuromotor assessment performed.

8.1. Informed Consent/Assent

The subject (or the subject's parent or legal guardian) will be given an oral explanation of the study, including information about the study drug and the study procedures and will have all questions adequately addressed. Written informed consent and, where appropriate as required by local regulations, assent, must be obtained from the subject (or the subject's parent or legal guardian) before any study-related procedures are performed and whenever the study procedures change or new safety information becomes available that may affect the subject's willingness to participate.

8.2. Demographics and Medical History

Demographic data will include date of birth, sex, age, race, and ethnicity, and will be collected during the Screening period. If local regulations do not allow a full date of birth to be collected, the birth date will be reported to the extent allowed (eg, month and year or year only).

A complete medical and surgical history will be obtained at screening including, but not limited to the elements described in Table 22.

Table 22: Elements of Complete Medical and Surgical History

Subject Medical/Surgical History

- Disease manifestations and approximate age at presentation
  - Motor function delay/difficulties evaluated at distances corresponding to short distances (eg, at home – 5 meters) moderate distances (eg, at school - 50 meters), or longer sustained distances (eg, shopping at a mall – 500m)
    - Walking ability (normal, difficulty with longer distances; difficulty with mobility around house; requires wheelchair)
    - Ability to climb stairs with or without help (normal, minimal/moderate impairment, severe impairment, not able)
  - Cognitive and language delays/difficulties
- Age at ARG1-D diagnosis and method of confirmation of diagnosis (arginine levels; RBC enzyme activity; DNA testing; other)
  - Newborn screening history
- Evolution of disease progression: age relevant symptoms such as spasticity, seizures, muscle cramps, hyperammonemia first noted
- Previous and current disease management including age of initiation and compliance with severe protein restriction and use of ammonia scavengers if prescribed, use of antispasmodics (eg, baclofen); Botulinum toxin-containing medications use; tendon release or other surgical procedures; physical therapy
- Current status with respect to the following:
  - All currently active and relevant medical and surgical conditions,
  - Seizures
  - Muscle cramps
  - Frequency of hyperammonemia and hyperammonemic episodes requiring hospitalization for the 2 years prior to screening
  - Liver injury (elevated ALT/AST) and or dysfunction
  - Other hospitalizations
    - Reason(s) for hospitalization and date(s) if known
  - Diet, including severe protein restriction and assessments of routine intake of EAA supplementation and/or the use of ammonia scavengers and vitamins

Family Medical History

- Total number of siblings and vital status (alive/dead)
- Health status living siblings (healthy/motor abnormalities and/or developmental delay/other health problems; specify)
- Number of siblings with confirmed diagnosis ARG1-D
  - Disease manifestations and approximate age at presentation OR newborn screening history
  - Age at diagnosis and method of diagnosis (arginine, RBC enzyme activity; DNA testing/other, Newborn Screening)

8.3. Physical Examination (Including Neurological Examination)

A complete, age-appropriate physical examination will be performed by the Investigator or qualified designee at the first screening visit.

The complete physical examination will include:

- Evaluation of the head, eyes, ears, nose, throat, cardiovascular, dermatological, musculoskeletal, respiratory, gastrointestinal, and neurological systems.

Given the characteristics of the disease in subjects with ARG1-D, the neurological examination will be detailed:

- Gait assessment, including balance and absence/presence of tiptoe walking
- Motor function
  - Muscle strength
  - Coordination
  - Muscle tone including presence /absence clonus
  - Reflexes
- Level of consciousness, mental status, speech, vision, and sensory assessments.

Physical examinations performed after the first screening visit (Schedules of Assessments in Table 1 through Table 15) will include the neurologic evaluation described above as well as a brief review of systems (head, eyes, ears, nose, throat, cardiovascular, dermatological, musculoskeletal, respiratory and gastrointestinal systems) and relevant physical examination to document significant physical changes from Baseline. Before administration of the first dose of study drug, any new or worsened abnormalities will be recorded as medical history. After the initiation of study drug administration, changes in neurologic examination will be noted on the appropriate section of the eCRF. Other clinically significant changes noted on the brief physical examination will be recorded as AEs. No rectal or pelvic examinations are required.

Weight will be obtained as described in the Schedules of Assessments (Table 1 through Table 15), and prior to dosing with the subject in socks or bare feet and without braces, walkers, or similar appendages.

For pediatric subjects <18 years of age, height and head circumference will be collected for the clinical growth assessments (Section 8.9) at Screening and Visits 13 and 24.

8.4. Neuromotor Assessments

Several clinical outcomes assessments of neurological and neuromotor function will be conducted (Table 23). The timing of these assessments is specified in the Schedules of Assessments (Table 1 through Table 15). All neuromotor clinical outcomes assessments will be administered by appropriately qualified professionals and performed consistently between visits and clinical sites. Assessments may be conducted over more than 1 day. Where allowed, these assessments may be videotaped at Baseline and at subsequent timepoints thereafter. A subject who completes a Baseline assessment will continue with that assessment during follow up, even if they are out of age range for the test during follow up. Efforts will be made to ensure that the same assessor administers all neuromotor assessments. At visits where neuromotor assessments are conducted, the 2MWT will be the first neuromotor assessment performed.

Table 23: Neuromotor Function Clinical Outcomes Assessments

Domain Assessed	Test Name	Age Range	Time Required
Locomotion / Mobility / Endurance	2-Minute Walk Test	3 to 85	4 minutes
	Functional Mobility Scale and Gillette Functional Assessment Questionnaire	All ages	10 minutes
	Caregiver and Clinician Global Impression of Change<br>Caregiver and Clinician Global Impressions of Severity	All ages	5 minutes
Motor Function	9-Hole Pegboard	3 to 85 years	4 minutes
	Gross Motor Function Measure-88, Parts D and E	≥5 months	up to 60 minutes
	Modified Ashworth Scale	All ages	10 minutes

8.4.1. 2-Minute Walk Test

This is a performance outcomes measure of mobility, with the 2-Minute Walk Test (2MWT) being a shorter modification of the 6-minute version (eg, NIH Toolbox motor domain – 2MWT [Bohannon 2014]). The standardized methodology as defined by the ATS (American Thoracic Society 2002) will be followed. The 2MWT has been validated over a large spectrum of age groups, including children (Bohannon 2018) and adults (Bohannon 2014). Furthermore, both the 6-minute and a shorter modification have been used successfully in both children (Geiger 2007, Maher 2008) and in adults with cerebral palsy (Andersson 2006, Ammann-Reiffer 2018), which is also characterized by spasticity. Results of the 2MWT will be recorded as distance completed in meters and percent change from Baseline. Subjects will be instructed to wear shoes that securely cover their entire foot (eg, sneakers, etc) rather than sandals or slippers, etc when performing the 2MWT. Given the relevance to the key secondary endpoint, this assessment will be performed before any other assessments conducted on a given day.

8.4.2. Functional Mobility Assessment

The Functional Mobility Assessment (FMA) is a combination of 2 assessments, the Functional Mobility Scale (FMS) (Graham 2004), and the Gillette Functional Assessment Questionnaire (GFAQ) (Novacheck 2000). The FMS is a 6-point scale from Level 1 (uses wheelchair, stroller, scooter, shopping cart, wagon, or is carried OR walks for exercise only with highly specialized/supportive walker OR does limited stepping with significant support/assistance from another person) to Level 6 (independent walking and running on all surfaces without assistive devices or help from another person) that assesses the need for assistive devices for walks of 3 different lengths: 5 meters, 50 meters, and 500 meters. The GFAQ is a parent/caregiver assessment consisting of a single question describing a child's ability to walk, using a 10-point scale from Level 1 (cannot take any steps at all) to Level 10 (walks, runs, and climbs on uneven terrain and does stairs without difficulty or assistance; is typically able to keep up with peers). Combined, the 2 assessments enable an understanding of support required and daily function, in multiple contexts, of a subject's mobility.

8.4.3. 9-Hole Pegboard Test

Manual dexterity (hand function) is a fine motor skill that represents an individual's ability to coordinate the fingers and manipulate objects in a timely manner. Such ability greatly impacts a person's performance in daily activities, such as self-care tasks, typing on a computer keyboard, messaging on a cell phone, completing work-related tasks, and engaging in leisure activities. This fine motor skill ability is negatively impacted in subjects with ARG1-D (Waisbren 2018).

The NIH Toolbox contains the 9-Hole Pegboard Dexterity Test, which is a simple test of manual dexterity for assessment of fine motor skills. It records the time required for the participants to accurately place and remove 9 plastic pegs into a plastic pegboard. This test takes approximately 4 minutes to administer and is recommended for ages 3 to 85 years (Wang 2011).

8.4.4. Gross Motor Function Measures

The Gross Motor Function Measure (GMFM-88, also referred to as GMFM) is a clinical measure designed to evaluate gross motor function by observing the subject's ability to initiate and complete certain movements. For this study, only Dimensions D and E will be assessed. Motor function decreases with increasing GMFCS level in this assessment. A scoring system is used by a trained observer for each item of the GMFM covering 2 dimensions: standing (Part D) and walking, running, and jumping (Part E). The GMFM is a clinical measure designed to evaluate changes in different aspects of gross motor function ability in children with cerebral palsy. For this study the GMFM-88 is being used. The scores from each component can be looked at separately or as part of the GMFM Total score. The components being used in this study (Parts D and E) and what they assess are as follows:

- Part D assesses standing
- Part E assesses walking, running, and jumping.

8.4.5. Modified Ashworth Scale

The MAS was developed to assess the spasticity of patients with central nervous system lesions and is used to measure the resistance to passive movement about a joint due to spasticity. The scale utilizes a scoring scale of 0 (no spasticity) to 4 (total rigidity) with 6 scoring choices as shown below:

Score	Modified Ashworth Scale
0	No increase in tone
1	Slight increase in tone manifested by a catch and release or by minimal resistance at the end of the ROM when moved in flexion or extension
1+	Slight increase in tone manifested by a catch, followed by a minimal resistance throughout the remainder (less than half) of the ROM
2	More marked increase in tone through most of the ROM, but affected parts easily moved
3	Considerable increase in tone; passive movement difficult
4	Limb rigid in flexion or extension

ROM = range of motion.

The test is well tolerated and easy to perform. Care should be taken as the scale cannot differentiate between spasticity and soft tissue stiffness. The scale has been analyzed for inter- and intra-rater reliability (Meseguer-Henarejos 2018).

8.5. Adaptive Behavior

The Vineland Adaptive Behavior Scales II (VABS-II) will be administered to the subject (as appropriate) and/or to the parent or caregiver. All evaluations will be administered by an appropriately qualified professional. Table 24 provides details on adaptive behavior assessments.

The VABS-II is a scale designed to measure adaptive behavior of individuals from birth to age 90 years (Sparrow 2005). The VABS-II contains 4 domains: communication, daily living skills, socialization, and motor skills. The domains are made up of 11 subdomains in which the scores are added to form the domain composite scores. The 4 domain composite scores then combine to form the adaptive behavior composite for those individuals aged birth to 6 years 11 months. Three domain composite scores (communication, daily living skills, and socialization) combine to form the adaptive behavior composite for those ages 7 through 90 years.

Table 24: Adaptive Behavior

Domain Assessed	Test Name	Age Range	Time Required
Adaptive Behavior	Vineland Adaptive Behavior Scales II (Caregiver-reported)	All ages	30-60 minutes

8.6. Assessments of Caregiver and Clinician Global Impressions

8.6.1. Caregiver and Clinician Global Impressions of Change

The Caregiver and Clinician Global Impressions of Change are questions independently administered to the subject's caregiver and clinician regarding their impression of change in quality of mobility and in adaptive behavior during the double-blind portion of the study. These questions are asked, as described in Table 2, at each post-Baseline visit in the randomized period during which 2MWT, GMFM-D, GMFM-E, FMA, and/or Vineland II are assessed. Where possible, the same caregiver and same clinician will respond to the questions throughout the study.

8.6.1.1. Questions Regarding Change to Be Independently Administered to Caregivers and Clinicians

For Both Caregivers and Clinicians

1. Considering both the subject's quality and confidence in walking, what is your impression of how the subject's ability to walk has changed during the study?
2. Considering the subject's dependence on assistive devices (walking aids), what is your impression of how the subject's ability to move from one place to another has changed during the study?

For Caregivers Only

3.
   a. What is your impression of how gross motor skills (movements of the whole body, arms and legs: eg, catching a ball, balancing, climbing, jumping on a trampoline, playing tag, running races) have changed in the subject since the start of the study?
   b. What is your impression of how fine motor skills (movements of the hands and fingers: eg, coloring with crayons, cutting with scissors, playing with small objects such as LEGOs or beads, using a knife and fork to eat, tying shoelaces, holding a pencil, drawing or writing) have changed in the subject since the start of the study?
   c. What is your impression of how daily living skills (activities for independent living: eg, feeding themselves, bathing, toileting, dressing, grooming) have changed in the subject since the start of the study?
   d. What is your impression of how communication skills (eg, following instructions, telling stories, commenting on events) have changed in the subject since the start of the study?
   e. What is your impression of the how socialization skills (eg, sharing toys, playing with peers, showing concerns for others) have changed in the subject since the start of the study?
   f. Considering your answers to parts (a) through (e) of this question, what is your impression of how the ability to adapt to their environment has changed in the subject since the start of the study?

8.6.1.2. Possible Responses

The possible responses for each question are:

- much worse
- worse
- no clear change
- better
- much better

8.6.2. Caregiver and Clinician Global Impressions of Severity

The Caregiver and Clinician Global Impressions of Severity are single questions independently administered to the subject's caregiver and clinician regarding their impression of the current (ie, point-in-time) severity of the subject's deficits in mobility and adaptive behavior. These questions are asked, as described in Table 2, at Baseline and at each visit in the randomized period during which 2MWT, GMFM Parts D and E, FMA, and/or Vineland II are assessed. Where possible, the same caregiver and same clinician will respond to the questions throughout the study.

8.6.2.1. Questions Regarding Severity to Be Independently Administered to Caregivers and Clinicians

For Both Caregivers and Clinicians

1. Considering both the subject's quality and confidence in walking, what is your impression of subject's ability to walk relative to other people of a similar age who do not have Arginase 1 Deficiency (eg, other family members, friends, children, school classmates)?
2. Considering the subject's dependence on assistive devices (walking aids), what is your impression of the subject's ability to move from one place to another relative to other people in the same age bracket who do not have Arginase 1 Deficiency (eg, other family members, friends, children, school classmates)?

For Caregivers Only

3.
   a. What is your impression of the subject's gross motor skills (movements of the whole body, arms, and legs: eg, catching a ball, balancing, climbing, jumping on a trampoline, playing tag, running races) relative to other people in the same age bracket who do not have Arginase 1 Deficiency (eg, other family members, friends, children, school classmates)?

b. What is your impression of the subject's fine motor skills (movements of the hands and fingers: eg, coloring with crayons, cutting with scissors, playing with small objects such as LEGO or beads, using a knife and fork to eat, tying shoelaces, holding a pencil, drawing or writing) relative to other people in the same age bracket who do not have Arginase 1 Deficiency (eg, other family members, friends, children, school classmates)?
c. What is your impression of how daily living skills (activities for independent living: eg, feeding themselves, bathing, toileting, dressing, grooming) relative to other people in the same age bracket who do not have Arginase 1 Deficiency? (eg, other family members, friends, children, school classmates)?
d. What is your impression of the subject's communication skills (eg, following instructions, telling stories, commenting on events) relative to other people in the same age bracket who do not have Arginase 1 Deficiency? (eg, other family members, friends, children, school classmates)?
e. What is your impression of the subject's socialization skills (eg, sharing toys, playing with peers, showing concerns for other) relative to other people in the same age bracket who do not have Arginase 1 Deficiency? (eg, other family members, friends, children, school classmates)?
f. Considering your answers to parts (a) through (e) of this question, what is your impression of the subject's ability to adapt to their environment, relative to other people in the same age bracket who do not have Arginase 1 Deficiency? (eg, other family members, friends, children, school classmates)?

8.6.2.2. Possible Responses

The possible responses for each question are:

- very much worse than others
- much worse than others
- worse than others
- a little bit worse than others
- no clear difference from others

8.6.3. Use of Caregiver and Clinician Global Impressions as Anchors

The responses to the questions defined in Section 8.6.1.1 and Section 8.6.2.1 will be used as anchors to support the pre-defined clinically meaningful within-subject change for the 2MWT, GMFM-D, GMFM-E, FMA, and VABS-II.

8.7. Seizure Frequency

The dates of all seizures will be recorded. The date and (approximate) time, along with the type of seizure, will be noted. Collection of seizure frequency information will begin at Baseline and continue throughout the study.

8.8. Neurocognitive and Quality-of-Life Assessments

Neurocognitive, developmental, and quality of life (QoL) tests to evaluate the intelligence, memory, and QoL of subjects are presented in Table 25 and will be assessed at the time points indicated in the Schedules of Assessments (Table 1 through Table 15). The VABS-II, PedsQL Measurement Model for the PedsQL, and the Short Form Zarit Burden Interview (ZBI-12) are assessments that will be administered to the subject (as appropriate) and/or to the parent or caregiver. All other assessments will be administered only to the subject (as is age-appropriate). These assessments may be videotaped at Baseline and at subsequent timepoints thereafter in accordance with local laws and regulations.

All evaluations will be administered by an appropriately qualified professional, and where possible, by the same assessor throughout the study. Assessments may be conducted over more than 1 day. The selection of the appropriate test instrument administered to a subject is normally dependent on the calendar age of the subject. For assessments that may overlap by age in any one domain, the assessor will evaluate the subject's cognitive and functional capabilities to determine which test is most appropriate for the subject. The assessor will attempt to administer the most advanced test to a given subject based on their chronological and/or developmental age. If the assessor determines during administration of the assessment that the subject's functional capabilities are not appropriate for the more advanced test, they will end the assessment with the advanced test and administer a less advanced test instead. These assessments may be videotaped at Baseline and at subsequent timepoints thereafter in accordance with local laws and regulations.

Table 25: Neurocognitive and Quality of Life (QoL) Assessments

Domain Assessed	Test Name	Age Range[a]	Time Required
Intelligence<br>1 assessment per subject depending on age	Bayley Scales of Infant Development III	2 to 3.5 years	40 minutes
	Wechsler Preschool and Primary Scale of Intelligence IV	2.5 to 7.6 years.	45 to 60 minutes
	Wechsler Intelligence Scale for Children V	6 to 16 years	45 to 65 minutes
	Wechsler Adult Intelligence Scale IV (WAIS)	16 years and older	30 to 40 minutes
Quality of Life<br>1 assessment per subject depending on age	PedsQL Measurement Model for the Pediatric Quality of Life Inventory (Subject- or parent-reported)	2 to 18 years	5-10 minutes
	36-Item Short Form Health Survey (SF-36)	19 years or greater	10 minutes
Caregiver QoL	Short Form Zarit Burden Interview (ZBI-12)	Completed by Caregiver, All ages[b]	30 minutes

[a] A subject who completes a Baseline assessment will continue with that assessment during follow up, even if they are out of age range for the test during follow up.
[b] The same caregiver will complete the questionnaire throughout the study wherever possible.

8.8.1. Neurocognitive Assessments

A subject who completes a Baseline assessment will continue with that assessment during follow up, even if they are out of age range for the test during follow up.

8.8.1.1. Wechsler Adult Intelligence Scale IV (WAIS-IV)

The WAIS-IV test has 10 core subtests and 5 supplemental subtests (Wechsler 2008). The test measures abilities in 4 main categories. These include reasoning, retention of information, processing and organization of information, and verbal comprehension. Factors such as creativity, individuality, or judgment are not incorporated into the test. Each category is scored individually, and the composite score is used to obtain the intelligence quotient (IQ).

The General Ability Index (GAI) for this test consists of the Similarities, Vocabulary, and Information subtests from the Verbal Comprehension Index and the Block Design, Matrix Reasoning, and Visual Puzzles subtests from the Perceptual Reasoning Index. The GAI is clinically useful because it can be used as a measure of cognitive abilities that are less vulnerable to impairments of processing and working memory.

8.8.1.2. Wechsler Intelligence Scale for Children V (WISC-V)

The WISC-V generates a Full-Scale IQ (formerly known as an intelligence quotient or IQ score) that represents a child's general intellectual ability (Wechsler 2014). It also provides 5 primary index scores: Verbal Comprehension Index, Visual Spatial Index, Fluid Reasoning Index, Working Memory Index, and Processing Speed Index. These indices represent a person's abilities in discrete cognitive domains. Five ancillary composite scores can be derived from various combinations of primary or primary and secondary subtests.

The GAI for this test provides the practitioner a summary score that is less sensitive to the influence of working memory and processing speed. The GAI is calculated from the General Ability Sum of Scaled Scores, which is the sum of scaled scores for 3 Verbal Comprehension subtests (ie, Vocabulary, Comprehension, and Similarities) and 3 Perceptual Reasoning subtests (ie, Block Design, Matrix Reasoning, and Picture Concepts).

8.8.1.3. Wechsler Preschool and Primary Scale of Intelligence IV (WPPSI-IV)

The WPPSI is an intelligence test designed for children ages 2 years 6 months to 7 years 7 months. WPPSI–IV is a revision of the WPPSI-R (Wechsler 1989, Wechsler 2008) and the WPPSI-III (Wechsler 2002). It provides subtest and composite scores that represent intellectual functioning in verbal and performance cognitive domains, as well as a composite score that represents a child's general intellectual ability (ie, full scale IQ). It consists of 14 subtests. They are designated as 1 of 3 types: core, supplemental, or optional. The core subtests are required for the computation of the verbal, performance, and full-scale IQ.

The WPPSI–IV provides verbal and performance IQ scores as well as a full-scale IQ score. In addition, the processing speed quotient can be derived for children aged 4 years to 7 years 3 months, and a general language composite can be determined for children in both age bands (2 years 6 months to 3 years 11 months and 4 to 7 years 3 months). Children in the 2 years 6 months to 3 years 11 months age band are administered only 5 of the subtests: receptive vocabulary, block design, information, object assembly, and picture naming.

8.8.1.4. Bayley Scales of Infant and Toddler Development III (BSID-III)

The BSID-III has been validated for children <3.5 years of age and takes approximately 25 to 60 minutes to administer (Centers for Disease Control and Prevention [CDC] 2002, Connolly 2012). The test is administered to the subject and was designed to identify young children with development delays. The BSID-III assesses developmental function across 5 domains: cognition; language (expressive and receptive); motor (fine and gross motor functioning); and social, emotional, and adaptive behavior.

8.8.2. Quality-of-Life Assessments

8.8.2.1. Measurement Model for the Pediatric Quality of Life Inventory (PedsQL)

The PedsQL Measurement Model for the PedsQL is a modular approach to measuring health-related quality of life in healthy children and adolescents and those with acute and chronic health conditions (Varni 2009). The PedsQL Measurement Model integrates seamlessly both generic core scales and disease-specific modules into one measurement system.

The 23-item PedsQL Generic Core Scales were designed to measure the core dimensions of health as delineated by the World Health Organization, as well as role (school) functioning. The 4 Multidimensional Scales are Physical Functioning (8 items) Emotional Functioning (5 items) Social Functioning (5 items) School Functioning (5 items). The 3 Summary Scores are: Total Scale Score (23 items), Physical Health Summary Score (8 items), Psychosocial Health Summary Score (15 items).

The tool is practical and can be completed in 5 minutes. It is developmentally appropriate (ages 2-18 years): Child self-report Ages 5 to 7 years, 8 to 12 years, 13 to 18 years; Parent Proxy Report: Ages 2 to 4 years, 5 to 7years, 8 to 12 years, 13 to 18 years. The Investigator/ testing psychologist will determine whether a child is able to complete the self-report at study Baseline. For children where they are not able, or it is not appropriate for them to complete the self-report, eg, children 2 to 4 years old, the Parent Proxy report will be used.

8.8.2.2. 36-Item Short Form Health Survey (SF-36)

The SF-36 is a 36-item scale constructed to survey health status and QoL (Ware 1993). The SF-36 assesses 8 health concepts: limitations in physical activities because of health problems; limitations in social activities because of physical or emotional problems; limitations in usual role activities because of physical health problems); bodily pain; general mental health (psychological distress and well-being); limitations in usual role activities because of emotional problems; vitality (energy and fatigue); and general health perceptions. The standard form of the instruments asks for participants to reply to questions according to how they have felt over the previous week.

8.8.2.3. Short Form Burden Interview (ZBI-12)

The ZBI (Zarit 1985) is a widely used tool to describe caregiver burden and has been well validated in various caregiving samples. The ZBI-12 (Bedard 2001) is a 12-item short version of the ZBI. The scores from the ZBI-12 have previously been assessed to have good correlation with the original version in caregivers of advanced cancer subjects. All versions feature statements that are ranked by informants as occurring never, rarely, sometimes, quite frequently, or nearly always.

8.9. Assessment of Growth

Longitudinal changes in anthropometric data will be assessed using Z-scores and percentiles for weight for age, stature for age, and head circumference for age determined from the publicly available CDC growth curves for subjects ≤18 years of age (CDC 2002). For subjects >18 years of age, age-normalized percentiles for height will be computed at Baseline only, and weight percentiles will not be computed.

8.10. Exit Interview

Given the small sample size and the limitations associated with traditional psychometric testing in rare indications, caregiver exit interviews may be undertaken by independent reviewers to support clinically meaningful change in the clinical outcome assessments administered in the trial. Exit interviews are considered to assist in better understanding the changes observed from Baseline in disease-defining and disease impact concepts. Exit interviews help in understanding the subject's experience in the trial on an individual basis, particularly if a subject chooses to discontinue the clinical trial before completing all study procedures. Where allowed, these assessments may be audiotaped. Exit interviews will be conducted at the end of the randomized, 24-week double-blind period.

8.11. Clinical Laboratory Tests

Laboratory samples will be obtained and forwarded to the central laboratory or processed locally according to the Schedules of Assessments (Table 1 through Table 15). The tests to be obtained are provided in Table 26.

Table 26: Clinical Laboratory Assessments

Lab Panel	Tests
Hematology	RBCs, WBCs, hemoglobin, hematocrit, WBC differential count (neutrophils, lymphocytes, eosinophils and basophils), platelet count, mean corpuscular hemoglobin concentration, mean corpuscular hemoglobin, mean corpuscular volume, mean platelet volume, and RBC distribution width
Serum Chemistry	Sodium, potassium, chloride, bicarbonate, calcium, phosphate, BUN, creatinine, glucose, lactate dehydrogenase, AST, ALT, alkaline phosphatase, total bilirubin, total protein, and albumin
Other Biomarkers	Growth hormone, IGF-1,<br>*Baseline only:*<br>S-transthyretin/prealbumin; S-transferrin/TIBC, S-RBP retinol binding protein
Arginine, Ornithine, GC	Arginine, ornithine, guanidinoacetic acid, α-N-acetylarginine, α-keto-δ-guanidinovaleric acid, argininic acid, creatine, homoarginine, or others.
Amino acids	The amino acid panel excluding ornithine and arginine will be provided to the Investigators. Amino acids to be assayed are: Taurine, Aspartic Acid, Hydroxyproline, Threonine, Serine, Asparagine, Glutamic Acid, Glutamine, Proline, Glycine, Alanine, Citrulline, Valine, Methionine, Cystine, Isoleucine, Leucine, Tyrosine, Phenylalanine, a-Amino-n-butyric acid, Tryptophan, Lysine and Histidine.
Ammonia (Local Lab)	Plasma ammonia will be measured at Baseline and at regular intervals throughout the study. Samples will be analyzed shortly after collection by the local laboratory due to the lability of this sample.
Urinalysis	Appearance, glucose, ketones, blood, protein, nitrate, bilirubin, specific gravity, pH, and leukocyte esterase. If positive for blood, protein trace, or leukocyte esterase then include microscopy.
Coagulation	aPTT, PT/INR
Pregnancy Test	Serum pregnancy tests will be performed by the central laboratory for all female subjects of childbearing potential at the Screening and End of Study visits. Serum or urine pregnancy tests will be performed at the local lab as noted at other visits as described in the Schedules of Assessments. Subjects who have a confirmed positive pregnancy test at any time during the study will be discontinued from further study drug administration and will be followed for pregnancy outcome.
Mutation analysis and arginase activity in RBC	Mutation analysis of the arginase 1 gene and arginase activity in RBCs will be assessed for all subjects at the central laboratory (Section 8.2). Mutation analysis is required for all subjects in accordance with local or country laws or regulations. If subject's medical record contains the results of a previous mutation analysis, that is sufficient. Some countries may require a separate, optional consent/assent; if a subject does not consent/assent, they may still be enrolled in the study.
Phenylbutyrate Metabolite Analysis	Samples for phenylbutyrate metabolite analysis will be collected at Baseline and during any hyperammonemic episode.
Serum tryptase and serum complement C3	Serum tryptase and serum complement C3 will be measured at Baseline and in the event of a hypersensitivity reaction at the central lab. Samples will be obtained at 3 and 24 hours after the start of the reaction.
ADAs	Samples for ADAs will be collected at Baseline and pre-dose at regular intervals throughout the study according to the Schedules of Assessments. In the event of a hypersensitivity reaction, additional ADA samples will be taken at the time of the event and as clinically indicated thereafter.
PK	Samples for the determination of pegzilarginase levels will be collected. The results will remain blinded until the study is completed.

Clinically significant abnormal laboratory test results will be followed until a return to normal or Baseline values. Details for the collection, processing, storage, and shipment of samples can be found in the study laboratory manual.

8.12. DNA Sample

The Sponsor intends to analyze pharmacogenetics in the pegzilarginase program to determine how genetic variations may affect clinical parameters and ARG1-D pathogenesis. DNA sequences, including both the protein coding sequence and sequences that regulate gene transcription, messenger ribonucleic acid (mRNA) stability and the efficiency of protein translation that may be investigated include: (1) Arginase I; (2) genes coding for other proteins involved in biology that may contribute to and/or modify the disease phenotype of ARG1-D; and (3) genes that may modify susceptibility to AEs. Where local regulations permit and subject to discretionary approval from each center's Institutional Review Board (IRB)/Independent Ethics Committee (IEC), and separate subject/caregiver consent/assent, a blood sample for DNA extraction may be collected from each subject during the screening period, or as soon as practical thereafter. The DNA may be used as part of a later pooled analysis, which may include a determination of the spectrum of arginine mutations in subjects with ARG1-D who have provided a separate consent/assent where applicable, and the relationship between gene mutation, PK/PD, safety, efficacy, and susceptibility to development of anti-drug antibodies (ADA). The Sponsor may only analyze DNA sequences within genes relevant to the mode of action and response to pegzilarginase, including variants important in understanding AEs, and candidate genes with a potential role in the etiology, pathogenesis, and progression of ARG1-D. No additional testing will be performed on the samples collected in the study. DNA samples will be stored by the Sponsor, or designee, in a secure, monitored, and controlled environment until analysis, and will be destroyed by the Sponsor after all worldwide obligations have been met, or sooner if required by local regulations. Refer to the Study Operations Manual and/or laboratory manual for further details regarding the collection, processing, and storage of these samples.

8.13. Home Health Care

If appropriate, in the opinion of the Investigator in consultation with the Sponsor, subjects may have laboratory samples taken outside of the investigational site (ie, subject's home or other appropriate location). In such cases, these protocol procedures will be performed by appropriately qualified and trained home health care personnel, under the direction of the Investigator. These personnel will complete all relevant source documents and provide this information (including safety data) to the Investigator in a timely manner. When appropriate, blood samples may be collected and prepared for shipping to the laboratory by home healthcare personnel. In instances where this is not appropriate in the opinion of the Investigator (and in consultation with the Sponsor), subjects will be required to attend visits at the investigational site.

If a subject is dosed outside of the investigational site, appropriately qualified and trained home health care personnel, under the direction of the Investigator, will be responsible for performing the dosing and conducting the safety and efficacy assessments and procedures required by the protocol. These personnel will complete all relevant source documents and provide this information (including safety data) to the Investigator in a timely manner. In instances where this is not appropriate in the opinion of the Investigator (and in consultation with the Sponsor), subjects will be required to attend visits at the investigational site. Refer to the Remote Visit Manuals for more detailed information about study visits conducted by home health care personnel.

8.14. Disease Management Plan and 3-Day Diary Diet Record

The current recommended disease management approach involves a common framework of severe protein restriction and EAA supplementation and/or the use of ammonia scavengers (also referred to herein as IDM). These facets of disease management are individualized for each subject by titration of the different components to reduce plasma arginine levels and ensure an adequate intake of protein for proper growth and development while avoiding hyperammonemia. General guidelines suggest that the diet for subjects with ARG1-D will include approximately 50% of the protein from natural food sources and approximately 50% from amino acid supplements. This recommendation may be adjusted as clinically indicated to achieve the IDM goals appropriate for a specific subject.

Subjects will be maintained on the IDM regimen prescribed prior to enrollment by the treating Investigator throughout the study. The prescribed IDM regimen will not be modified during the study unless clinically indicated in the opinion of the Investigator. The rationale for any change in IDM, and the date and nature of any significant (>±15% of Baseline) prescribed changes, will be recorded in the eCRF.

Subjects will be required to maintain dietary protein intake levels that are consistent with their Baseline levels for the entire duration of the randomized double-blind AND the 8-week blinded period of the LTE period of the study. A consistent diet is defined as one in which the prescribed and consumed natural intact protein, medical food EAA, and calories are <±15% from Baseline.

Subjects and/or their caregivers will be instructed to record all dietary intake by the study subject (natural food, medical food/EAAs, and EAA supplementation) for 3 consecutive days prior to all clinic visits during the screening and Baseline period and before double-blind Visits 6, 12, 18, and 24. At these visits, the prescribed IDM regimen will also be recorded. The date and time of the last dose of EAAs must be recorded for each day of the 3-day diary diet record.

8.15. Arginine, Ornithine, and Guanidino Compounds

Blood samples for analysis of plasma arginine, ornithine, and guanidino compounds will be drawn at time points designated in the Schedules of Assessments (Table 1 through Table 15) and sent to the central laboratory. Blood samples will be collected to evaluate arginine-derived guanidino compounds and sent to the central lab. Potential guanidino compounds to be measured may include guanidinoacetic acid, α-N-acetylarginine, α-keto- δ-guanidinovaleric acid, argininic acid, creatine, or others.

Assessments are specified by the time points listed in the Schedules of Assessments (Table 1 through Table 15) and blood samples will be collected to explore the Baseline and treatment-associated changes to the levels of guanidino compounds.

To maintain study blinding, Investigators will not be provided with arginine, ornithine, or guanidino compound results from the double-blind or blinded follow-up periods until after the study is complete and the blind is formally broken.

8.16. Plasma Amino Acids and Ammonia

Samples for analysis of plasma amino acids (other than arginine) will be drawn at timepoints designated in the Schedules of Assessments (Table 1 through Table 15) and sent to the central lab. Sampling for amino acids will be performed after approximately 4-hours of fasting from food and EAAs, except where operationally and/or clinically impractical. The date and time of each sample will be recorded. Fasting status, including time of last meal and last EAA administration, will be recorded in the eCRF.

Samples for analysis of plasma ammonia will be drawn at time points designated in the Schedules of Assessments (Table 1 through Table 15) and sent to the site's local lab. The results of the ammonia test will be recorded in the appropriate section of the eCRF. Care will be exercised in obtaining samples for ammonia testing. Institution-specific procedures for obtaining and processing samples for ammonia testing **must** be followed carefully to insure an accurate result. A repeat test will be considered to confirm elevation.

The amino acid panel excluding ornithine and arginine will be provided to the Investigators. Ornithine and arginine are blinded for clinical study purposes, and therefore will not be provided to Investigators and will remain blinded until the end of the study.

8.17. Vital Signs and Height and Weight

Vital signs (including blood pressure, pulse, respirations, temperature, height, and weight) will be assessed at the timepoints specified in the Schedules of Assessments (Table 1 through Table 15).

8.18. Electrocardiogram

A 12-lead ECG will be obtained after approximately 5 minutes of rest in the supine position using equipment at the site at the visits noted on the Schedules of Assessments (Table 1 through Table 15). An unscheduled ECG will be obtained as clinically indicated for cardiovascular symptoms. The Investigator or designee will evaluate the ECGs for abnormalities and any abnormalities will be specified as clinically significant (CS) or not clinically significant (NCS).

8.19. Electroencephalogram

In addition to clinically overt seizures, a high frequency of abnormal electroencephalogram (EEG) activity has been observed in subjects with ARG1-D (Carvalho 2012, Huemer 2016, De Deyn 1997). Electroencephalography is a test used to evaluate electrical activity in the brain. The EEG is defined as electrical activity of an alternating type recorded from the scalp surface after being picked up by metal electrodes and conductive media (Teplan 2002). Electroencephalogram results have been shown to be abnormal in a considerable proportion of subjects with ARG1-D, with EEG recordings showing slow diffuse activity, and generalized epileptic activity (Carvalho 2012, De Deyn 1997). All subjects will be consented for Baseline and follow-up routine (ie, not sleep, ambulatory or video telemetry), outpatient EEG recordings. Screening and Week 24 recordings will be performed for all subjects.

8.20. Pharmacokinetics

Blood samples will be collected for measurement of serum concentrations of pegzilarginase as specified in the Schedules of Assessments (Table 1 through Table 15). The timing of the sample collection is described in Table 27.

Table 27: Pharmacokinetic Sampling Times

	Baseline	Visit 12	Visit 24
**Pre-infusion (within 1 hour)**	X	X	X
**After completion of infusion**			
1 hour (±30 minutes)	X	X	X
2 hours (±30 minutes)	X	X	X
4 hours (±1 hour)	X	X	X
24 hours (±4 hours)	X	X	X
96 hours (±8 hours)	X	X	X
168 hours (±24 hours, but prior to next dose)	X	X	X

Efforts will be made to collect the samples within the specified windows where feasible, but samples collected outside the windows will not be considered protocol deviations.

These samples will be shipped to the central PK lab and may be batched. Instructions for the collection and handling of biological samples will be provided by the Sponsor. The actual date and time (24-hour clock time) of each sample will be recorded.

Drug concentration information that would unblind the study will not be reported to investigative sites or blinded personnel until the study has been unblinded.

8.21. Anti-Drug Antibody Testing

Blood samples for ADA testing will be drawn as described in the Schedules of Assessments (Table 1 through Table 15) and sent to the central lab. Samples will be analyzed both for antibodies against pegzilarginase and against PEG. When samples are positive and confirmed for anti-pegzilarginase ADA, specificity will be tested against the intermediate (Co-Arg1) as well as the endogenous arginase 1. When positive and confirmed for PEG isotype specificity may be determined. An unscheduled sample for ADA assessment will be obtained in the event of a moderate or severe hypersensitivity reaction at the time of the event or if clinically indicated (as determined by the Investigator). Subjects who test positive for ADAs will be followed and re-tested approximately every 2 to 3 months until the antibody levels return to pre-exposure levels.

8.22. Follow-Up

All subjects will return to the study site or be contacted by phone for a follow-up assessment approximately 2 weeks following the last dose of study drug. See Section 7.1 for required follow-up for subjects that discontinue the study early.

If a subject fails to return for scheduled visits, a documented effort must be made to determine the reason. If the subject cannot be reached by telephone after 2 attempts, a certified letter will be sent to the subject (or the subject's legally authorized representative, if appropriate) requesting contact with the Investigator. This information will be recorded in the study records.

9. SAFETY ASSESSMENTS

9.1. Adverse Events, Serious Adverse Events, and Laboratory Test Abnormalities

Adverse event (AE) means any untoward medical occurrence associated with the use of a drug in humans, whether or not considered drug related.

An AE may include intercurrent illnesses or injuries that represent an exacerbation (increase in frequency, severity, or specificity) of pre-existing conditions (eg, worsening of asthma).

Whenever possible, it is preferable to record a diagnosis as the AE term rather than a series of terms relating to a diagnosis.

The reporting period for nonserious and serious AEs (SAEs) is the period from the signing of informed consent continuing through the last study follow-up visit. All AEs will be followed to resolution or until they become insignificant to further follow up. Resolution is defined as the return to Baseline status or stabilization of the condition, with the expectation that it will remain chronic.

The Investigator will assess AEs for severity, for relationship to IMP, and whether the event meets one or more of the definitions of an SAE (Section 9.1.1).

The severity of each AE will be assessed by the Investigator or qualified designee using the categories defined as follows:

- **Mild** – Event usually transient, requires minimal or no treatment and does not generally interfere with the participant's daily activities.
- **Moderate** – Event usually alleviated with additional specific therapeutic intervention. The event interferes with usual activities of daily living, causing discomfort, but poses no significant or permanent risk of harm to the research participant result in a low level of inconvenience or concern with the therapeutic measures. Moderate events may cause some interference with functioning but poses no significant or permanent risk of harm to the participant.
- **Severe** – Event interferes a participant's usual daily activity and may require intensive therapeutic intervention. Of note, the term "severe" does not necessarily equate to "serious".

The Investigator or their qualified designee will determine the relationship of an AE to the study treatment using the categories defined in Table 28.

Table 28: Adverse Event Relationship to Study Medication

Relationship	Description
Not Related	No relationship between the experience and the administration of study drug; related to other etiologies such as concomitant medications or subject's clinical state.<br>• Exposure to the IMP has not occurred; **or**<br>• The administration of the IMP and the occurrence of the AE are not reasonably related in time; **or**<br>• The AE is considered likely to be related to an etiology other than the use of the IMP; that is, there are no facts [evidence] or arguments to suggest a causal relationship to the IMP.
Unlikely Related	The current state of knowledge indicates that a relationship is unlikely. Poor timing, other causes more likely:<br>• The administration of the IMP and the occurrence of the AE are related in time but the relationship is improbable based on the current state of knowledge; **and**<br>• The AE could be explained equally well by factors or causes other than exposure to the IMP.<br>To be used when the exclusion of drug causality of a clinical event seems most plausible
Possibly Related	A reaction that follows a plausible temporal sequence from administration of the study drug and follows a known response pattern to the suspected study drug. The reaction might have been produced by the subject's clinical state or other modes of therapy administered to the subject, but this is not known for sure.<br>Good timing but other causes possible:<br>• The administration of the IMP and the occurrence of the AE are reasonably related in time; **and**<br>• The AE could not be explained equally well by factors or causes other than exposure to the IMP.<br>To be used when drug causality is one of the other possible causes
Probably Related	A reaction that follows a plausible temporal sequence from administration of the study drug and follows a known response pattern to the suspected study drug. The reaction cannot be reasonably explained by the known characteristics of the subject's clinical state or other modes of therapy administered to the subject.<br>Good timing, other causes unlikely, withdrawal challenge.<br>• The administration of IMP and the occurrence of the AE are reasonably related in time; **and**<br>• The AE is more likely explained by exposure to the IMP than by other factors or causes; **and**<br>• The AE follows a clinically reasonable response on withdrawal (dechallenge).
Definitely Related	A reaction that follows a plausible temporal sequence from administration of the study drug and follows a known response pattern to the suspected study drug and can be confirmed with a positive re- challenge test or supporting laboratory data.

AE = adverse event; IMP = investigational medicinal product.

To classify AEs, preferred terms will be assigned by the Sponsor or contract research organization (CRO) to the original terms entered on the eCRF, using the Medical Dictionary for Regulatory Activities (MedDRA) terminology.

9.1.1. Serious Adverse Events

An SAE is defined as any AE that:

- Results in death
- Is life threatening, that is, places the subject at immediate risk of death from the event as it occurred. This definition does not include a reaction that, had it occurred in a more severe form, might have caused death.
- Requires in-subject hospitalization (with a minimum 24-hour stay) or prolongation of an existing in-subject hospitalization.
- Results in persistent or significant disability or incapacity. An event qualifies as resulting in a persistent or significant disability or incapacity if it involves a substantial disruption of the subject's ability to carry out usual life functions. This definition is not intended to include experiences of relatively minor or temporary medical significance.
- Is a congenital anomaly or birth defect, ie, an AE that occurs in the child or fetus of a subject or the partner of a subject exposed to IMP prior to conception or during pregnancy.
- Is an important medical event or serious medical condition that does not meet any of the above criteria but may jeopardize the subject or require medical or surgical intervention to prevent 1 of the outcomes listed above. Examples of such events are intensive treatment in the emergency room, allergic bronchospasm, blood dyscrasias or convulsions that do not result in hospitalization, or development of drug dependency or drug abuse.

More than one of the above criteria may apply to any specific event.

The reporting period for SAEs is the period from the signing of informed consent continuing through the last study follow-up visit. SAEs of which the Investigator becomes aware within 30 days after the subject completes the study will be reported to the Sponsor. SAEs must be followed by the Investigator until resolution or until the event becomes insignificant to further follow up. Resolution of an SAE is defined as the return to Baseline status or stabilization of the condition, with the expectation that it will remain chronic.

Any SAE, whether or not considered related to study drug, will be reported in a timely manner, within 24 hours, to the Sponsor or its safety designee by fax or scanned and emailed using the study-specific SAE Report Form. In addition to the outcome of the SAE, any medication or other therapeutic measures used to treat the event will be recorded on the appropriate eCRF page(s). Investigators will not wait to collect additional information that fully documents the event before notifying the Sponsor or its safety designee of an SAE. The Sponsor may be required to report certain SAEs to regulatory authorities within 7 calendar days of being notified about the event; therefore, it is important that Investigators submit additional information requested by the Sponsor, the Medical Monitor, and/or their safety designee in a timely manner once they become aware of it.

Reporting of SAEs to the IRB/EC will be completed in compliance with the standard operating procedures and policies of the IRB/EC and with applicable regulatory requirements.

A completed initial SAE Report Form signed by the Investigator must be transmitted to the Sponsor's safety representative (Synteract) at SafetyFax@Synteract.com. Synteract will forward the SAE Report Form to the Sponsor.

Additional follow-up information, if required or available, must be transmitted to the Sponsor's safety representative (Synteract) at SafetyFax@Synteract.com within 24 hours of receipt. This follow-up information will be completed on an SAE Report Form and placed with the original SAE information and kept with the appropriate section of the eCRF and/or study file.

The Investigator is encouraged to discuss with the Medical Monitor any AEs for which the issue of seriousness is unclear or questioned. Contact information for the Medical Monitor is provided in the Study Operations Manual.

9.2. Treatment of Overdose

Not applicable.

9.3. Adverse Events of Special Interest

Hypersensitivity reactions (Section 9.3.1), injection site reactions (ISRs) (Section 9.3.2), and hyperammonemic episodes (Section 9.3.3) are defined as AESIs for this clinical study. Hypersensitivity and ISRs are considered an AESI due to their potential relationship to study drug administration. Hyperammonemia is a known disease manifestation of ARG1-D. Because of the potential impact of hyperammonemic episodes on subject safety and the validity of clinical assessments, hyperammonemia is considered an AESI to enable tracking and more careful monitoring of contributing factors and the frequency/severity of these episodes.

9.3.1. Hypersensitivity Reactions

A hypersensitivity reaction will be considered an AESI. If, at any time during the study, the Investigator observes symptoms that he/she considers to be consistent with a hypersensitivity reaction related to administration of study drug, the symptoms will be recorded as an AE(s) and designated as a hypersensitivity reaction. Examples of hypersensitivity reactions include, but are not limited to, flushing, fever and/or chills, pruritus, urticaria, facial and/or tongue edema, chest pain, dyspnea, wheezing, stridor, hypotension or hypertension, bradycardia, or tachycardia.

In the case of a hypersensitivity reaction, additional tryptase, complement C3, and ADA samples will be taken at 3 and 24 hours after the start of the reaction. Any subject who experiences a hypersensitivity reaction must safely receive at least 4 subsequent doses at the study site, after which dosing by home health care may be considered if deemed safe by the Investigator and after consultation with the Sponsor.

As with any enzyme replacement therapy, medications and equipment for the treatment of hypersensitivity reactions must be available for immediate use in case of unexpected severe hypersensitivity reactions. These supplies include, but are not limited to, oxygen, acetaminophen, antihistamines (eg, diphenhydramine, parenteral and oral), and epinephrine.

Infusion or dosing of study medication will be interrupted in any subject developing signs or symptoms of a hypersensitivity reaction. Hypersensitivity reactions will be treated according to standard of care at the clinical site. The infusion or dosing may be resumed if, in the opinion of the Investigator, and after consultation with the Sponsor, the symptoms can be safely managed. Hypersensitivity reactions observed in Study 101A were typically managed with antihistamines and a slower infusion rate. The benefits and risks of corticosteroid treatment will be carefully considered given their potential to cause hyperammonemia in this patient population. Additional information is available in the IB and Pharmacy Manual.

General guidelines for classifying the severity of a reaction are provided below:

- Mild reactions are defined as self-limiting, spontaneously-resolving reactions that may be managed with a temporary cessation of dosing or a reduction in the infusion rate.
- Moderate reactions are defined as reactions that do not resolve with simple measures; require extended observation and therapy interruption.
- Severe reactions are defined as reactions that require intervention to prevent a serious outcome.

9.3.2. Injection Site Reactions

Injection site reactions (ISRs) are common effects of biologic treatments given via SC injection. They are a local phenomenon defined as a constellation of symptoms, including swelling, erythema, pruritus, and pain around the site of the injection. ISRs should be managed according to standard of care at the clinical site.

9.3.3. Hyperammonemic Episodes

Hyperammonemia occurs in some patients with ARG1-D as part of the disease manifestations. Therefore, plasma ammonia will be monitored throughout the study according to the Schedules of Assessments (Table 1 through Table 15). The effects of hyperammonemia are not always symptomatic. Cases of elevated ammonia in the absence of related symptoms will be recorded as an AE of “elevated ammonia.” A hyperammonemic episode will be defined as a subject with a confirmed ammonia level ≥100 µM and 1 or more symptoms related to hyperammonemia requiring hospitalization or emergency room management, with or without admission to the hospital.

Upon the occurrence of any hyperammonemic episode, 1 or more repeat plasma ammonia samples will be taken to confirm the plasma ammonia level and document resolution (local lab). A phenylbutyrate metabolite sample will be taken at the same time as 1 of the ammonia samples is collected, preferably the first, and will be shipped to the central laboratory. Hyperammonemia will be managed according to standard of care at the clinical site.

In the event of a hyperammonemic episode the following additional information will be captured in the eCRF:

- Precipitating factors such as infection, menstruation, concomitant illness, noncompliance with ammonia scavengers, changes in ammonia scavenger prescription and changes in diet will be recorded in the CRF.
- Clinical symptoms and signs such as nausea, vomiting, headache, lethargy, altered mental state, confusion, mood change, neurological abnormalities, and seizures will be recorded in the CRF.

9.3.4. Reporting an Adverse Event of Special Interest

Any AESI will be reported in a timely manner, within 24 hours, to the Sponsor or its safety designee by fax or scanned and emailed using the study-specific AESI Report Form. In addition to the outcome of the AESI, any medication or other therapeutic measures used to treat the event will be recorded on the appropriate eCRF page(s). Investigators will not wait to collect additional information that fully documents the event before notifying the Sponsor or its safety designee of an AESI.

A completed initial AESI Report Form signed by the Investigator must be transmitted to the Sponsor's safety representative (Synteract) at SafetyFax@Synteract.com. Synteract will forward the AESI Report Form to the Sponsor.

Additional follow-up information, if required or available, must be transmitted to the Sponsor's safety representative (Synteract) at SafetyFax@Synteract.com within 24 hours of receipt. This follow-up information should be completed on an AESI Report Form and placed with the original AESI information and kept with the appropriate section of the eCRF and/or study file.

9.4. Laboratory Test Abnormality

The Investigator will routinely review all safety laboratory results. Out-of-range laboratory test values that the Investigator considers to be a clinically significant change from the subject's Baseline value or previous values will be reported on the AE page of the eCRF.

9.5. Independent Safety Review Committee

Primary oversight for subject safety will be the responsibility of the Investigators and Sponsor. Additional advisory oversight of subject safety will be provided by an independent Safety Review Committee (SRC) composed of individuals with pertinent medical expertise, who will serve in advisory capacity to the Sponsor to provide an additional level of oversight to minimize the chance that clinical study subjects are exposed to unreasonable or unnecessary risks. To enable the assessment of the benefit:risk of pegzilarginase during the study, members of the independent SRC will have access to unblinded individual subject-level safety data as needed. If the SRC has any safety concerns, they may obtain full access to subjects' treatment assignment.

Collectively, the SRC members will have methodological and clinical expertise relevant to the study design and population. SRC membership will begin before the start of the clinical trial and is expected to last for its duration. Core members of the SRC will not participate in the trial as Investigators or sub-Investigators, as members of any team otherwise participating in the trial, or in any other capacity that may compromise their privileged activities on the SRC. SRC members may not have financial or other conflicts of interests, as outlined in the SRC charter.

The SRC will perform periodic reviews of individual and/or aggregated safety data for this study as deemed appropriate based on accumulating data. During these meetings, the SRC will review available cumulative study safety data, including all SAEs, AEs, hypersensitivity reactions, study discontinuations, and the full set of safety laboratory results. The SRC will also perform ad-hoc reviews of safety data on an as-needed basis in the event of emerging safety signals of clinical concern in one or more subjects. Following each regular and ad- hoc review of safety data, the SRC will indicate whether dosing of study medication may continue (or be resumed) for all subjects or a subset of subjects in the study. The composition and activities of the SRC will be outlined in the SRC Charter, which will be ratified during the initial meeting of the SRC and will be in place prior to commencement of dosing in this study.

10. STATISTICAL CONSIDERATIONS

10.1. Sample Size Determination

Based on preliminary data from Study 101A and Study 102A, the mean decrease from Baseline in plasma arginine after 24 weekly doses of pegzilarginase is estimated to be 95% compared to the placebo group. On the log scale this corresponds to a change of 3 units [log(0.05)=-3], the estimate for the common standard deviation of the change from Baseline on the log scale is 1.4 units. Under these assumptions, a total sample size of 30 subjects, with 20 subjects assigned to pegzilarginase and 10 assigned to placebo, provides over 99% power to detect a statistically significant difference between the 2 treatment arms.

Based on assumed effect sizes from preliminary data from Study 101A and Study 102A, and a sample size of 30 subjects, simulations have shown that the power to show statistical significance in 1 or both analyses of the key secondary endpoints of mobility (change from Baseline in 2MWT and change from Baseline in GMFM-E) using a Hochberg procedure to control the Type 1 error is at least 90%. Further details relating to the simulations can be found in the SAP.

Although an exact screen failure rate cannot be accurately predicted based on available data, a sufficient number of subjects will be screened so that at least 30 subjects in the Full Analysis Set (FAS) have at least 1 follow-up measurement on which clinical response can be assessed (2MWT and GMFM-E). Subjects who discontinue after their first dose but before completing the double-blind portion ("dropouts") will not be replaced; Section 10.3 describes how all subjects, including dropouts, are included in the analyses of efficacy and safety.

10.2. Populations for Analyses/Analysis Sets

For purposes of analysis, the following populations (analysis sets) are defined in Table 29.

Table 29: Analysis Sets

Analysis Set	Description
Consented Set	All subjects who sign an ICF. This population is used to create the CONSORT diagram.
Randomized Set	All subjects in the Consented population who are randomized to a blinded study treatment.
Full Analysis Set	All subjects who are randomized and who receive a least 1 dose of blinded study treatment. All safety and efficacy analyses are performed on this set.
Per Protocol Set	All subjects in the Full Analysis Set who have completed their planned 21st, 22nd, 23rd, and 24th doses and who have assessable key secondary endpoints and no major/important protocol deviations related to the assessment of efficacy.
PK Set	All subjects with sufficient PK data to permit meaningful analysis.

10.3. Statistical Analyses

The statistical analysis plan (SAP) will be developed and finalized before any unblinding of the double-blinded period, and will describe the subject populations to be included in the analyses, and procedures for accounting for missing, unused, and spurious data. This section is a summary of the planned statistical analyses of the primary and secondary endpoints.

For efficacy analyses of pegzilarginase vs placebo, subjects will be categorized according to the treatment to which they were randomized. For safety analyses, subjects will be categorized according to the treatment that they actually received. Subjects receiving a single dose of pegzilarginase will be categorized in the pegzilarginase treatment arm for all safety outputs.

When all subjects have completed the blinded portion of the study to LTE01 and its assessments, and the database has been frozen, the data will be formally unblinded and analyzed.

10.3.1. Efficacy Analyses

An overview of efficacy analyses is provided in Table 30. Baseline deficits for other secondary clinical endpoints are defined in Table 31.

Table 30: Detailed Overview of Statistical Methodology for Efficacy Endpoints

Endpoint	Statistical Analysis Methods
Primary	Change from Baseline after 24 weeks of study treatment in logged plasma arginine will be compared between groups using an ANCOVA model at α=0.05, 2-sided. Details of the covariates and the models will be included in the SAP.<br>If the primary analysis is not statistically significant, formal hypothesis testing will stop. If it is statistically significant, then formal hypothesis testing will proceed with the key secondary mobility endpoints.<br>Sensitivity analyses of the primary analysis will be performed using Wilcoxon rank sum tests. Details will be provided in the SAP.
Key Secondary Endpoints (Mobility)	Change from Baseline at Week 24 in 2MWT and GMFM-E will be compared between groups using separate ANCOVA models. Details of the covariates and the models will be included in the SAP.<br>The global Type 1 error level of the key secondary endpoints will be controlled using a Hochberg procedure with the primary endpoint acting as a gatekeeper.<br>The method used to formally control the global Type 1 error rate for the later subsequent secondary endpoints will be detailed in the SAP. However, p-values for the secondary analyses will be shown for descriptive purposes regardless of whether the formal hypothesis testing has stopped.
Secondary	Details of multiplicity adjustments within the secondary endpoints will be provided in the SAP.<br>• Proportions of subjects with final arginine <200 µM will be compared between randomized treatments using Fisher's Exact Test.<br>• Proportions of subjects with final arginine 40 to 115 µM will be compared between randomized treatments using Fisher's Exact Test.<br>• Change from Baseline at Week 24 in GMFM-D will be compared between randomized treatments using an ANCOVA model. Details of the covariates and the models will be included in the SAP.<br>• Change from Baseline at Week 24 in the overall VABS-II Adaptive Behavior Composite and in each of the 4 individual components (Communication Domain, Daily Living Skills Domain, Socialization Domain, Motor Skills Domain) will be compared between randomized treatments using an ANCOVA model. Details of the covariates and the models will be included in the SAP.<br>• Change from Baseline at Week 24 in the FMA assessments (FMS 5, FMS 50, FMS 500, and GFAQ) will be compared between randomized treatments using an ANCOVA model. Details of the covariates and the models will be included in the SAP.<br>• Ornithine and GCs will be assessed using the same statistical methods as for the primary endpoint.<br>• PK will be assessed using standard Non-Compartmental Analysis techniques.
Tertiary	Analyses for tertiary endpoints will be described in the SAP.

Table 31: Definition of Baseline Deficits for Other Secondary Clinical Endpoints*

Domain	Assessment	Component	Definition of Baseline Deficit
Mobility**	FMA	FMS 5 meters<br>FMS 50 meters<br>FMS 500 meters<br>GFAQ	Level 1 – Level 5<br>Level 1 – Level 5<br>Level 1 – Level 5<br>Level 2 – Level 9
Adaptive Behavior	VABS-II	Adaptive Behavior Composite<br>Communication Domain<br>Daily Living Skills Domain<br>Socialization Domain<br>Motor Skills Domain	Standard Score ≤ 85<br>Standard Score ≤ 85<br>Standard Score ≤ 85<br>Standard Score ≤ 85<br>Standard Score ≤ 85

FMA = Functional Mobility Assessment; FMS = Functional Mobility Scale; VABS-II = Vineland Adaptive Behavior Scale, Version 2.
* Definition of Baseline deficit is calculated from data in (Bohannon 2018) for subjects 3 to 17 years of age and (Bohannon 2015) in subjects ≥18 years of age. Definition of clinical response is from Bohannon 2015.
** Baseline deficit is not applicable for MAS.

10.3.1.1. Details for Analysis of the Primary Endpoint

For the primary endpoint, change from Baseline in plasma arginine, the plasma arginine values will be log transformed prior to analysis.

The Baseline value will be defined as the mean of all logged plasma arginine values (analyzed at the designated central laboratory) obtained during the screening/Baseline period and prior to the first dose of blinded study treatment. If a subject was re-screened, only those values taken during the final screening period are used in the computation of Baseline arginine.

The final follow-up arginine level will be defined as the mean of the last 4 prior-to-dosing logged values obtained during the double-blind period that meet the following criteria: (1) the sample date occurred after the scheduled date for the 20th dose, and (2) the prior 2 doses were administered as planned, ie, at approximately 7 days and 14 days prior to collection of the arginine sample. If at least 1, but fewer than 4, values meet the criteria, then only those values will be included in the mean. If none of the values meet the criteria, then the last single post-Baseline arginine logged value will be included.

If no post-Baseline arginine values were obtained, then the change from Baseline will be imputed as 0. Otherwise, change from Baseline will be equal to (Final follow-up arginine level logged value – Baseline plasma arginine logged value).

Change from Baseline to final follow-up arginine level as defined in this section will be compared between randomized groups using an ANCOVA model using 2-sided α=0.05. The Baseline arginine value will be included as a covariate in the model. Details of any other covariates and class effects to be included in the ANCOVA will be detailed in the SAP.

Once exponentiated, the change from baseline in logged values will represent a ratio. The difference in change from baseline values will represent a relative ratio in plasma arginine for subjects treated with pegzilarginase vs subjects treated with placebo.

As a sensitivity analysis, change from Baseline to final follow-up arginine level as defined in this section will be compared between pegzilarginase and placebo with a Wilcoxon rank sum test.

To assess the impact of dropouts, a second sensitivity analysis will be performed using a Wilcoxon rank sum test where those subjects who drop out for reasons deemed related to IMP will receive lower rank than those who complete all 24 doses or who drop out for reasons clearly not related to IMP. Details will be provided in the SAP.

Supportive subgroup analyses may be performed if there are ≥4 subjects in each subgroup. The process for determining subgroups of interest will be defined in the SAP.

10.3.1.2. Details for Analysis of the Key Secondary Mobility Endpoints

If the primary analysis is not statistically significant, formal hypothesis testing will stop and p-values for key secondary endpoints will be descriptive only. If the primary analysis is statistically significant, then formal hypothesis testing will proceed with the key secondary mobility endpoints.

The global Type 1 error level with respect to the testing of the 2 key secondary endpoints will be controlled using the Hochberg adjustment for multiplicity.

Change from Baseline at Week 24 in 2MWT and GMFM-E will be compared between randomized groups using separate ANCOVA models. The respective Baseline values will be included as a covariate in each model. Details of any other covariates and class effects to be included in the ANCOVA will be detailed in the SAP.

10.3.1.3. Details for Analysis of the Secondary Efficacy Endpoints

The Hochberg procedure will be used to formally control the global Type 1 error rate across the 2 key secondary endpoints. Type 1 error control for further secondary endpoints will be detailed in the SAP. However, p-values for the secondary analyses will be shown for descriptive purposes regardless of whether the formal hypothesis testing has stopped. Details of multiplicity adjustments within the secondary endpoints will be provided in the SAP.

10.3.1.3.1. Proportion of Subjects Achieving Arginine Recommended-Guidance Level of <200 µM

The proportions of subjects whose endpoint arginine value, as defined in Section 10.3.1.1, is below the recommended-guidance level of 200 µM after 24 weeks of study treatment will be compared between pegzilarginase and placebo using a Fisher’s exact test.

10.3.1.3.2. Proportion of Subjects Achieving Arginine Normalization (40 to 115 µM)

The proportions of subjects whose endpoint arginine value, as defined in Section 10.3.1.1, is within normal range (defined as 40 to 115 µM) after 24 weeks of study treatment will be compared between pegzilarginase and placebo using a Fisher’s exact test.

10.3.1.3.3. Additional Secondary Endpoints

Change from Baseline at Week 24 in GMFM-D will be assessed with the same methods used for 2MWT and GMFM-E.

Change from Baseline at Week 24 in the overall VABS-II Adaptive Behavior Composite and in each of the 4 individual components (Communication Domain, Daily Living Skills Domain, Socialization Domain, Motor Skills Domain) will be assessed with the same methods used for 2MWT and GMFM-E.

Change from Baseline at Week 24 in the FMA assessments (FMS 5, FMS 50, FMS 500, and GFAQ) will be assessed with the same methods used for 2MWT and GMFM-E.

These secondary endpoints will also be analyzed as tertiary endpoints using responder analyses incorporating the response definitions for 2MWT, GMFM-D, GMFM-E, FMA, and VABS-II detailed in Table 32, which includes the definition of clinically meaningful change for each endpoint.

Table 32: Additional Clinical Response Definitions

Domain	Assessment	Component	Definition of Response		
Mobility	2MWT	• Distance walked	Improvement by ≥9%		
	GMFM		GMFCS I	GMFCS II	GMFCS III
		• Part D	2.4	3.3	1.5
		• Part E	4.0	2.8	1.8
	FMA	• FMS 5 meters	Improvement by 1 level		
		• FMS 50 meters	Improvement by 1 level		
		• FMS 500 meters	Improvement by 1 level		
		• GFAQ	Improvement by 2 levels		
Adaptive Behavior	VABS-II	• Adaptive Behavior Composite	Improvement by ≥7.5 points		
		• Communication Domain	Improvement by ≥7.5 points		
		• Daily Living Skills Domain	Improvement by ≥7.5 points		
		• Socialization Domain	Improvement by ≥7.5 points		
		• Motor Skills Domain	Improvement by ≥7.5 points		

Note: Response definitions based on Ammann-Reiffer 2018 and Norman 2003.

A subject is considered a responder for the given secondary endpoint if the subject meets the definition of response for that endpoint.

An independent, blinded, anchor-based analysis of all double-blind assessments of 2MWT, GMFM Parts D and E, FMA, and VABS-II, performed prior to formal unblinding of the double-blind data, will be conducted in support of the predefined clinically meaningful changes.

10.3.1.3.4. Ornithine and Guanidino Compounds

Ornithine and GCs will be assessed with the same methodology as for arginine, although the p-values are considered descriptive.

10.3.2. Safety Analyses

Safety analyses will be performed on the FAS. Subjects will be presented as treatment received; subjects receiving a single dose of pegzilarginase will be categorized in the pegzilarginase treatment group for all safety outputs. A brief overview of statistical analysis methods for safety endpoints is provided in Table 33. Further details on these analyses and analyses of other safety endpoints will be described in the SAP.

Table 33: Brief Overview of Statistical Methodology for Safety Endpoints

Endpoint	Statistical Analysis Methods
Adverse Events	Summaries of proportion of subjects with specific AEs will be presented, including AEs by severity, AEs by relationship to study treatment, SAEs, and AEs leading to discontinuation. Note that reported relationship to study treatment will be dichotomized: AEs categorized as either not related or unlikely related to study treatment will be analyzed as "not related" to study treatment while AEs categorized as possibly, probably, or definitely related to study treatment will be analyzed as "related" to study treatment.
Laboratory Assessments	These will be summarized longitudinally using changes and percent changes from Baseline. Shift tables may also be created.
Vital Signs	Vital signs will be listed but will be summarized through vital-sign-related adverse events.

10.3.3. Other Analyses

Pharmacokinetic, PD, tertiary endpoints, and biomarker exploratory analyses will be described in the SAP, which will be finalized before any unblinding of the double-blind period. The PK and PD analyses will be presented separately from the main clinical study report (CSR).

10.3.4. Stratification of Randomization

Randomization in this study is stratified by severity of prior history of hyperammonemia. The purpose of this stratification is to avoid potential imbalance in this factor across randomized treatment groups. The main analyses will not be stratified for this factor, but its influence will be explored in supportive analyses described in the SAP.

The randomization strata, based on hyperammonemic episodes as defined in Section 9.3.2, are:

- ≥1 hyperammonemic episode within 90 days of consent or ≥2 hyperammonemic episodes within 365 days of consent
- No hyperammonemic episodes within 365 days of consent, or 1 hyperammonemic episode occurring >90 days prior to consent

10.4. Interim Analyses

No interim analyses are planned during the blinded portion of the study. When all subjects have completed the blinded portion of the study to LTE01 and its assessments, and the database has been frozen, the data will be formally unblinded and analyzed.

11. SUPPORTING DOCUMENTATION AND OPERATIONAL CONSIDERATIONS

11.1. Regulatory and Ethical Considerations

This study will be conducted in accordance with the protocol and with the following:

- Applicable International Council for Harmonisation (ICH) Good Clinical Practice (GCP) Guidelines
- Applicable laws and regulations

The protocol, protocol amendments, ICF, IB, and other relevant documents (eg, advertisements) must be submitted to an IRB/IEC by the Investigator and reviewed and approved by the IRB/IEC before the study is initiated. Amendments to the protocol require IRB/IEC approval before implementation of changes made to the study design, except for changes necessary to eliminate an immediate hazard to study subjects.

The Investigator will be responsible for the following:

- Providing written summaries of the status of the study to the IRB/IEC annually or more frequently in accordance with the requirements, policies, and procedures established by the IRB/IEC
- Notifying the IRB/IEC of SAEs or other significant safety findings as required by IRB/IEC procedures
- Providing oversight of the conduct of the study at the site and adherence to requirements of 21 CFR (Code of Federal Regulations), ICH guidelines, the IRB/IEC, European regulation 536/2014 for clinical studies (if applicable), and all other applicable local regulations

11.2. Privacy and Confidentiality

The confidentiality of subject records will be protected in accordance with applicable laws, regulations, and guidelines in all regions where the study is conducted.

After subjects have consented to take part in the study, the Sponsor and/or its representatives may review and copy their medical records and data collected during the study. These records and data may, in addition, be reviewed and copied by others including the following: independent auditors who validate the data on behalf of the Sponsor; third parties with whom the Sponsor may develop, register, or market pegzilarginase; national or local regulatory authorities; and the IRB(s)/EC(s) that gave approval for the study to proceed. The Sponsor and/or its representatives accessing the records and data will take all reasonable precautions in accordance with applicable laws, regulations, and guidelines to maintain the confidentiality of subjects' identities.

Subjects are assigned a unique identifying number; however, their initials and date of birth (or age where date of birth may not be collected) may also be collected and used to assist the Sponsor to verify the accuracy of the data (eg, to confirm that laboratory results have been assigned to the correct subject).

The results of studies containing subjects' unique identifying number, relevant medical records, and possibly initials and dates of birth, will be recorded. They may be transferred to, and used in, other countries which may not afford the same level of protection that applies within the countries where this study is conducted. The purpose of any such transfer would include supporting regulatory submissions, conducting new data analyses to publish or present the study results, or answering questions asked by regulatory or health authorities.

11.3. Data Quality Assurance

All subject data relating to the study will be recorded on the eCRF unless transmitted to the Sponsor or designee electronically (eg, laboratory data). The Investigator is responsible for verifying that data entries are accurate and correct by physically or electronically signing the eCRF.

The Investigator must maintain accurate documentation (source data) that supports the information entered in the eCRF.

The Investigator and institution must permit study-related monitoring, audits, IRB/IEC review, and regulatory agency inspections, and must provide direct access to source data documents.

11.4. Management of Deviations

Procedures for management of deviations are provided in the Study Operations Manual.

11.5. Electronic Case Report Forms and Source Documents

Electronic data capture (EDC) will be used. Therefore, subject data from source documents will be entered directly into the clinical database at the Investigator sites using eCRFs. The Investigator is responsible for assuring that source documentation is appropriately maintained, and that data entered into the eCRF is complete and accurate, and that entries and updates are performed in a timely manner.

The Sponsor or designees will review the data entered by investigational staff for completeness and accuracy. Electronic data queries stating the nature of the problem and requesting clarification will be created for discrepancies and missing values and sent to the investigational site via the EDC system. Designated Investigator site staff are required to respond promptly to queries and to make any necessary changes to the data.

For individual instances in which efficacy endpoints have not been captured due to COVID-19, the reasons for failing to obtain the efficacy assessment should be documented (eg, identifying the specific limitation imposed by COVID-19 leading to the inability to perform the protocol-specified assessment).

11.5.1. Study and Site Closure

The Sponsor reserves the right to terminate the study, and the Investigator reserves the right to terminate their involvement in the study, according to the study contract. The Investigator will notify the IRB/EC in writing of the study's completion or early termination and send a copy of the notification to the Sponsor.

11.5.2. Use of Information and Publication Policy

The Sponsor recognizes the importance of communicating medical study data and therefore encourages publication in reputable scientific journals and at seminars or conferences. The details of the processes of producing and reviewing reports, manuscripts, and presentations based on the data from this study will be described in the clinical study agreement between the Sponsor, CROs, and the Investigator.

Due to the confidential nature of this development program, the results of the study may not be published or publicly presented without the prior approval of the Sponsor. Any Investigator wishing to publish or present any study finding must present a manuscript or abstract to the Sponsor 120 days prior to submission for publication or presentation to provide the Sponsor an opportunity for review and comment.

11.5.3. Retention of Records

The Investigator must retain all study records required by the Sponsor and by the applicable regulations in a secure and safe facility. The Investigator must consult a Sponsor representative before disposal of any study records and must notify the Sponsor of any change in the location, disposition, or custody of the study files. The Investigator/institution must take measures to prevent accidental or premature destruction of essential documents (ie, documents that individually and collectively permit evaluation of the conduct of a study and the quality of the data produced), including paper copies of study records (eg, subject charts), as well as any original source documents that are electronic as required by applicable regulatory requirements.

All study records must be retained for at least 2 years after the last approval of a marketing application in the US or an ICH region and until (1) there are no pending or contemplated marketing applications in the US or an ICH region or (2) ≥2 years have elapsed since the formal discontinuation of clinical development of the IMP. The Investigator/institution will retain subject identifiers for ≥15 years after the completion or discontinuation of the study. Subject files and other source data must be kept for the maximum period of time permitted by the hospital, institution, or private practice, but not less than 15 years. These documents will be retained for a longer period, however, if required by the applicable regulatory requirements or by a Sponsor agreement. The Sponsor must be notified and will assist with retention will Investigator/institution be unable to continue maintenance of subject files for the full 15 years. It is the responsibility of the Sponsor to inform the Investigator/institution as to when these documents no longer need to be retained.

11.5.4. Study Monitoring, Auditing, and Inspections

Qualified individuals designated by the Sponsor will monitor all aspects of the study according to ICH/GCP and standard operating procedures for compliance with applicable government regulations. The Investigator agrees to allow these monitors direct access to the clinical supplies, dispensing, and storage areas and to the clinical files, including original medical records of the study subjects, and, if requested, agrees to assist the monitors. The Investigator and staff are responsible for being present or available for consultation during routinely scheduled site visits conducted by the Sponsor or its designees.

The review of the subjects' medical records will be performed in a manner to ensure that subject confidentiality is adequately maintained. Further details of the study monitoring will be outlined in a Monitoring Plan.

Members of the Sponsor's Quality Assurance Department or designees may conduct an audit of a clinical site or any vendor at any time during or after completion of the study. The Investigator or vendor will be informed if an audit is to take place and advised as to the scope of the audit.

Representatives of the FDA or other regulatory agencies may also conduct an inspection of the study site and corresponding documentation. If informed of such an inspection, the Investigator will notify the Sponsor immediately. The Investigator will ensure that the auditors have access to the clinical supplies, study site facilities, original source documentation, and all study files. All inspections and audits will be carried out giving consideration to data protection as well as subject confidentiality to the extent that local, state, and federal laws apply.

If planned on-site monitoring visits are not possible, use of central and remote monitoring programs should be optimized to maintain oversight of clinical sites.

12. ABBREVIATIONS

Abbreviation	Definition
ADA(s)	anti-drug antibodies (anti-AEB1102, anti-PEG antibodies)
AE	adverse event
AESI	adverse event of special interest
ALT	alanine aminotransferase
aPTT	activated partial thromboplastin time
ARG1-D	Arginase 1 Deficiency
AST	aspartate aminotransferase
ATS	American Thoracic Society
BL	Baseline
BMI	body mass index
BSID-III	Bayley Scales of Infant and Toddler Development-III
BUN	blood urea nitrogen
CDC	Centers for Disease Control And Prevention (United States)
CFR	Code of Federal Regulations
COS	Clinical Outcomes Solutions
CRA	clinical research associate
CRO	contract research organization
CS	clinically significant
CSR	clinical study report
EAA	essential amino acid
EC	Ethics Committee
ECG	electrocardiogram
eCRF	electronic case report form
EDC	electronic data capture
EEG	electroencephalogram
EODB	End of Double-blind Period
EORP	End of Randomized Period Visit
EOS	End of Study
ERT	enzyme replacement therapy
FDA	Food and Drug Administration
FMA	Functional Mobility Assessment
FMS	Functional Mobility Scale
FU	Follow-Up Visit

Abbreviation	Definition
GAI	General Ability Index
GC	guanidino compound
GCP	Good Clinical Practice
GFAQ	Gillette Functional Assessment Questionnaire
GMFCS	Gross Motor Function Classification System
GMFM	Gross Motor Function Measure-88
HIV	human immunodeficiency virus
IB	Investigator Brochure
ICF	informed consent form
ICH	International Council for Harmonisation
IDM	Individualized disease management: Severe protein restriction and essential amino acid supplementation (EAA) and/or the use of ammonia scavengers
IEC	Independent Ethics Committee
IGF-1	insulin-like growth factor 1
INR	international normalized ratio
IMP	investigational medicinal product: The terms “IMP” and “study drug” may be used interchangeably in the protocol.
IQ	intelligence quotient
IRB	Institutional Review Board
ISR	injection site reaction
IUD	intrauterine device
IUS	intrauterine hormone-releasing system
IV	intravenous(ly)
IXRS	interactive web/voice response system
LTE	long-term extension
MAS	Modified Ashworth Scale
MedDRA	Medical Dictionary for Regulatory Activities
mRNA	messenger ribonucleic acid
NCS	not clinically significant
NIH	National Institutes of Health (United States)
PD	pharmacodynamic(s)
PE	physical examination
PEACE	Pegzilarginase Effect on Arginase 1 Deficiency Clinical Endpoints
PedsQL	Pediatric Quality of Life
PEG	polyethylene glycol

Abbreviation	Definition
PK	pharmacokinetic(s)
PMA	phenylbutyrate metabolite analysis
PT	prothrombin time
QoL	quality of life
RBC	red blood cell
SAE	serious adverse event
SAP	statistical analysis plan
SC	Subcutaneous
Scr	Screening
SDC	Statistics and Data Corporation
SF-36	36-Item Short Form Health Survey
S-RBP	serum retinol binding protein
SRC	Safety Review Committee
2MWT	2-Minute Walk Test
TIBC	total iron-binding capacity
US	United States
VABS-II	Vineland Adaptive Behavior Scale, Second edition
WAIS-IV	Wechsler Adult Intelligence Scale IV
WBC	white blood cell
WISC-V	Wechsler Intelligence Scale for Children V
WPPSI-IV	Wechsler Preschool and Primary Scale of Intelligence IV
ZBI-12	Short Form Zarit Burden Interview

13. REFERENCES

Ammann-Reiffer, C.; Bastianen, C.; Van Hedel, H. Measuring change in gait performance of children with motor disorders: assessing the Functional Mobility Scale and the Gillette Functional Assessment Questionnaire walking scale. Developmental Medicine & Child Neurology: John Wiley & Sons Ltd on behalf of Mac Keith Press. Open Access Article 2018.

Andersson C, Asztalos L, Mattsson E. Six-minute walk test in adults with cerebral palsy. A study of reliability, Clin Rehabi. 2006;20(6):488-95.

American Thoracic Society 2002. ATS Statement: Guidelines for the six-minute walk test. Am J Respir Crit Care Med. 2002;166:111–117.

Bédard M, Molloy DW, Squire L, et al. The Zarit Burden Interview: a new short version and screening version. Gerontologist. 2001;41(5):652-7.

Bohannon RW, Bubela D, Magasi S, et al. Comparison of walking performance over the first 2 minutes and the full 6 minutes of the Six-Minute Walk Test. 2014 BMC Res Notes. 2014;7:269. doi: 10.1186/1756-0500-7-269.

Bohannon RW, Wang Y-C, Gershon RC. Two-minute walk test performance by adults 18 to 85 years: normative values, reliability, and responsiveness. Arch Phys Med Rehabil. 2015;96(3):472-7. doi: 10.1016/j.apmr.2014.10.006.

Bohannon RW, Wang Y-C, Bubela D, Gershon RC. Normative two-minute walk test distances for boys and girls 3 to 17 years of age. Phys Occup Ther Pediatr. 2018;38(1):39-45. doi: 10.1080/01942638.2016.1261981.

Burrage LC, Sun Q, Elsea SH, et al. Human recombinant arginase enzyme reduces plasma arginine in mouse models of arginase deficiency. Hum Mol Genet. 2015;24(22):6417-6427.

Carvalho DR, Brand GD, Brum JM, Takata RI, Speck-Martins CE, Pratesi R. Analysis of novel ARG1 mutations causing hyperargininemia and correlation with arginase I activity in erythrocytes. Gene. 2012;509:124–130. doi: 10.1016/j.gene.2012.08.003.

Cederbaum SD, Shaw KN, Spector EB, Verity MA, Snodgrass PJ, Sugarman GI. Hyperargininemia with arginase deficiency. Pediatr Res. 1979;13:827–833.

Cederbaum SD, Moedjono SJ, Shaw KN, Carter M, Naylor E, Walzer M. Treatment of hyperargininaemia due to arginase deficiency with a chemically defined diet. J Inherit Metab Dis. 1982;5:95–99.

Centers for Disease Control and Prevention (United States). CDC Growth Charts: United States (2002). Accessed from https://www.cdc.gov/growthcharts/cdc_charts.htm.

Connolly BH, McClune NO, Gatlin R. Concurrent Validity of the Bayley-III and the Peabody Developmental Motor Scale–2. Pediatr Phys Ther. 2012;24:345–352.

Cowley, D. M., et al. 1998. "Adult-onset arginase deficiency." J Inherit Metab Dis 21(6): 677-678.

De Deyn P, Marescau B, Qureshi I, Mori A. Hyperargininemia: a treatable inborn error of metabolism? The Fourth International Symposium on Guanidino Compounds in Biology and Medicine: Guanidino Compounds in Biology & Medicine II. 1997.

Desnick, R. J. and E. H. Schuchman 2012. "Enzyme replacement therapy for lysosomal diseases: lessons from 20 years of experience and remaining challenges." Annu Rev Genomics Hum Genet 13: 307-335.

Diez-Fernandez C, Rüfenacht V, Gemperle C, et al. Mutations and common variants in the human arginase (ARG1) gene: impact on patients, diagnostic, and protein structure considerations. Hum Mutat. 2018 May 3, doi: 10.1002/humu.23545.

European Medicines Agency Guidance on the Management of Clinical Trials During the Covid-19 (Coronavirus) Pandemic, Version 3, 28/04/2020, https://ec.europa.eu/health/sites/health/files/files/eudralex/vol-10/guidanceclinicaltrials_covid19_en.pdf.

European Medicines Agency Points to consider on implications of Coronavirus disease (COVID-19) on methodological aspects of ongoing clinical trials 26 June 2020, EMA/158330/2020 Rev. 1, Committee for Human Medicinal Products (CHMP), https://www.ema.europa.eu/en/documents/scientific-guideline/points-consider-implications-coronavirus-disease-covid-19-methodological-aspects-ongoing-clinical_en-0.pdf.

FDA Guidance on Conduct of Clinical Trials of Medical Products during COVID-19 Pandemic Guidance for Industry, Investigators, and Institutional Review Boards, March 2020, https://www.fda.gov/emergency-preparedness-and-response/coronavirus-disease-2019-covid-19/covid-19-related-guidance-documents-industry-fda-staff-and-other-stakeholders.

Geiger, R., et al. 2007. "Six-minute walk test in children and adolescents." J Pediatr 150(4): 395-399, 399.e391-392.

Graham, H. K., et al. 2004. "The Functional Mobility Scale (FMS)." J Pediatr Orthop 24(5): 514-520.

Häberle J, Boddaert N, Burlina A, Chakrapani A, et al. Suggested guidelines for the diagnosis and management of urea cycle disorders. Orphanet J Rare Dis. 2012;7:32. doi: 10.1186/1750-1172-7-32.

Huemer M, Carvalho DR, Brum JM, et al. Clinical phenotype, biochemical profile, and treatment in 19 patients with arginase 1 deficiency. J Inherit Metab Dis. 2016;39(3):331-340.

Investigator's Brochure, current version

Jain-Ghai, S., et al. 2011. "Arginase I deficiency: severe infantile presentation with hyperammonemia: more common than reported?" Mol Genet Metab 104(1-2): 107-111.

Lambert MA, Marescau B, Desjardins M, et al. Hyperargininemia: intellectual and motor improvement related to changes in biochemical data. J Pediatr. 1991;118(3):420-424.

Maher CA, Williams MT, Olds TS. The six-minute walk test for children with cerebral palsy. Int J Rehabil Res. 2008;31(2):185-8. doi: 10.1097/MRR.0b013e32830150f9.

Meseguer-Henarejos A-B, Sanchez-Meda J, Lopez-Pina J-A, et al. Inter-and intra-rater reliability of the Modified Ashworth Scale: a systematic review and meta-analysis. Eur J Phys and Rehab Medicine. 2018;54(4):576-590.

NIH 2016 Toolbox^{R} Scoring and Interpretation Guide for the iPad.

Norman GR, Sloan JA, Wyrwich KW. Point/Counterpoint: Interpretation of Changes in Health-related Quality of Life, The Remarkable Universality of Half a Standard Deviation. Medical Care, Volume 41, Number 5, pp 582-592, ©Lippincott Williams & Wilkins, Inc.

Novacheck TF, Stout JL, Tervo R. Reliability and validity of the Gillette Functional Assessment Questionnaire as an outcome measure in children with walking disabilities. J Pediatr Ortho. 2000;20:75-81.

Oeffinger, D. et al. Outcome tools used for ambulatory children with cerebral palsy: responsiveness and minimum clinically important differences. Dev Med Child Neurol**,** v. 50, n. 12, p. 918-25, Dec 2008. ISSN 1469-8749.

Prasad AN, Breen JC, Ampola MG, Rosman NP. Argininemia: a treatable genetic cause of progressive spastic diplegia simulating cerebral palsy: Case reports and literature review. J Child Neurol 1997;12(5):301-309.

Sakiyama, T., et al. 1984. "A successful trial of enzyme replacement therapy in a case of argininemia." Tohoku J Exp Med 142(3): 239-248.

Schlune A, vom Dahl S, Häussinger D, Ensenauer R, Mayatepek E. Hyperargininemia due to arginase I deficiency: the original patients and their natural history, and a review of the literature. Amino Acids. 2015;47:1751–1762.

Sparrow SS, Cicchetti VD, Balla AD. Vineland adaptive behavior scales. 2nd edition. American Guidance Service; Circle Pines, MN; 2005.

Summar ML, Koelker S, Freedenberg D, et al., The incidence of urea cycle disorders. Mol Genet Metab. 2013;110(1-2):179-80.

Teplan M. Fundamentals of EEG Measurement. Measurement Science Review, Volume 2, Section 2, 2002.

Varni JW, Limbers CA. The Pediatric Quality of Life Inventory: Measuring pediatric health-related quality of life from the perspective of children and their parents. Pediatr Clin N Am. 2009;56:843-863.

Waisbren SE, Cuthbertson D, Burgard P, et al. (2018). Biochemical markers and neuropsychological functioning in distal urea cycle disorders. J Inherit Metab Dis. 2018 Feb 8. doi: 10.1007/s10545-017-0132-5.

Wang YC, Magasi SR, Bohannon RW et al. Assessing dexterity function: a comparison of two alternatives for the NIH Toolbox. J Hand Ther. 2011 Oct-Dec;24(4):313-20.

Ware JE, Snow KK, Kosinski M, Gandek B. SF-36® Health Survey Manual and Interpretation Guide. Boston, MA: New England Medical Center, The Health Institute; 1993.

Wechsler D. (1989). WPPSI-R: Wechsler Preschool and Primary Scale of Intelligence. Psychological Corporation Harcourt Brace Jovanovich.

Wechsler D. (2002). Wechsler preschool and primary scale of intelligence (3rd ed.) San Antonio: The Psychological Corporation.

Wechsler D. (2008). WAIS-IV technical and interpretive manual. San Antonio, TX: Pearson, 2008.

Wechsler 2014 D. (2014). Wechsler intelligence scale for children (4th edition). Bloomington, MN: Pearson.

Wu, G. and S. M. Morris 1998. "Arginine metabolism: nitric oxide and beyond." Biochem J 336 (Pt 1): 1-17.

Zarit 1985 SH, Orr NK, Zarit JM. The hidden victims of Alzheimer's disease: Families under stress. New York: New York University Press, 1985.
