# Supplementary material for: Efficacy and safety of pegzilarginase in arginase 1 deficiency (PEACE): a phase 3, randomized, double-blind, placebo-controlled, multi-centre trial
Source: eClinicalMedicine. 2024 Jan 12;68:102405. doi: 10.1016/j.eclinm.2023.102405 (PMC10825663; doi:10.1016/j.eclinm.2023.102405)
Supplement: Supplementary Tables S1 and S2 [file mmc4.docx]

**Supplementary Table 1. Correlation of Plasma Arginine Levels With Guanidino Compounds and Ornithine at Week 24 (Full Analysis Set, post-hoc analysis)^a^**

| **Relationship Between Plasma Arginine And:** | **Pearson Correlation Coefficient** | **P Value** |
| --- | --- | --- |
| ARGA | 0.772 | <0.0001 |
| GAA | 0.712 | <0.0001 |
| GVA | 0.774 | <0.0001 |
| NAARG | 0.602 | 0.0003 |
| ORN | –0.508 | 0.0096 |

ARGA, argininic acid; GAA, guanidinoacetic acid; GVA, α-keto-δ-guanidinovaleric acid; NAARG, α-N-acetylarginine; ORN, ornithine.

^a^Correlation analysis included data for all patients in the full analysis set (N=31), with both treatment arms combined.

**Supplementary Table 2. Clinical Response Definitions**

| Domain | Assessment | Component | Definition of Response |
| --- | --- | --- | --- |
| Mobility | 2MWT | - Distance walked | Improvement by ≥9% |
|  | GMFM | - Part D - Part E | GMFCS I GMFCS II GMFCS III  2.4 3.3 1.5  4.0 2.8 1.8 |
|  |  |  |  |

Note: Response definitions based on Oeffinger et al. Outcome tools used for ambulatory children with cerebral palsy: responsiveness and minimum clinically important differences. Dev Med Child Neurol, v. 50, n. 12, p. 918-25, Dec 2008. ISSN 1469-8749; Bohannon et al. Comparison of walking performance over the first 2 minutes and the full 6 minutes of the Six-Minute Walk Test. BMC Res Notes. 2014;7:269.
